# Fractional flow reserve derived from computed tomography coronary angiography in the assessment and management of stable chest pain: the FORECAST randomized trial

**DOI:** 10.1093/eurheartj/ehab444

**Published:** 2021-07-16

**Authors:** Nick Curzen, Zoe Nicholas, Beth Stuart, Sam Wilding, Kayleigh Hill, James Shambrook, Zina Eminton, Darran Ball, Camilla Barrett, Lucy Johnson, Jacqui Nuttall, Kim Fox, Derek Connolly, Peter O’Kane, Alex Hobson, Anoop Chauhan, Neal Uren, Gerry Mccann, Colin Berry, Justin Carter, Carl Roobottom, Mamas Mamas, Ronak Rajani, Ian Ford, Pamela Douglas, Mark Hlatky

**Affiliations:** Faculty of Medicine, University of Southampton; Coronary Research Group, University Hospital Southampton; Coronary Research Group, University Hospital Southampton; Clinical Trials Unit, University of Southampton; Clinical Trials Unit, University of Southampton; Clinical Trials Unit, University of Southampton; Cardiothoracic Radiology, University Hospital Southampton; Clinical Trials Unit, University of Southampton; Clinical Trials Unit, University of Southampton; Clinical Trials Unit, University of Southampton; Clinical Trials Unit, University of Southampton; Clinical Trials Unit, University of Southampton; Imperial College, London, UK; Sandwell Hospital, Birmingham, UK; Dorset Heart Centre, University Hospitals Dorset, Bournemouth; Queen Alexandra Hospital, Portsmouth; Royal Victoria Hospital, Blackpool; Royal Infirmary, Edinburgh; Department of Cardiovascular Sciences, University of Leicester & NIHR Biomedical Research Centre, Glenfield Hospital, Leicester, UK; British Heart Foundation Glasgow Cardiovascular Research Centre, University of Glasgow; University Hospital of North Tees, Stockton on Tees; Derriford Hospital, Plymouth; Royal Stoke University Hospital, Stoke-on-Trent; Guy’s & St Thomas’ Hospital, London; Robertson Centre for Biostatistics, University of Glasgow, Glasgow; Duke University, Durham, NC, USA; Stanford University, Stanford, CA, USA

**Keywords:** Computed tomography coronary angiography, Cost analysis, Fractional flow reserve (FFR_CT_), Myocardial, Randomized controlled trial, Stable angina, Quality of life

## Abstract

**Aims:**

Fractional flow reserve (FFR_CT_) using computed tomography coronary angiography (CTCA) determines both the presence of coronary artery disease and vessel-specific ischaemia. We tested whether an evaluation strategy based on FFR_CT_ would improve economic and clinical outcomes compared with standard care.

**Methods and results:**

Overall, 1400 patients with stable chest pain in 11 centres were randomized to initial testing with CTCA with selective FFR_CT_ (experimental group) or standard clinical care pathways (standard group). The primary endpoint was total cardiac costs at 9 months. Secondary endpoints were angina status, quality of life, major adverse cardiac and cerebrovascular events, and use of invasive coronary angiography. Randomized groups were similar at baseline. Most patients had an initial CTCA: 439 (63%) in the standard group vs. 674 (96%) in the experimental group, 254 of whom (38%) underwent FFR_CT_. Mean total cardiac costs were higher by £114 (+8%) in the experimental group, with a 95% confidence interval from −£112 (−8%) to +£337 (+23%), though the difference was not significant (*P* = 0.10). Major adverse cardiac and cerebrovascular events did not differ significantly (10.2% in the experimental group vs. 10.6% in the standard group) and angina and quality of life improved to a similar degree over follow-up in both randomized groups. Invasive angiography was reduced significantly in the experimental group (19% vs. 25%, *P* = 0.01).

**Conclusion:**

A strategy of CTCA with selective FFR_CT_ in patients with stable angina did not differ significantly from standard clinical care pathways in cost or clinical outcomes, but did reduce the use of invasive coronary angiography.

## Graphical Abstract

**Figure:**
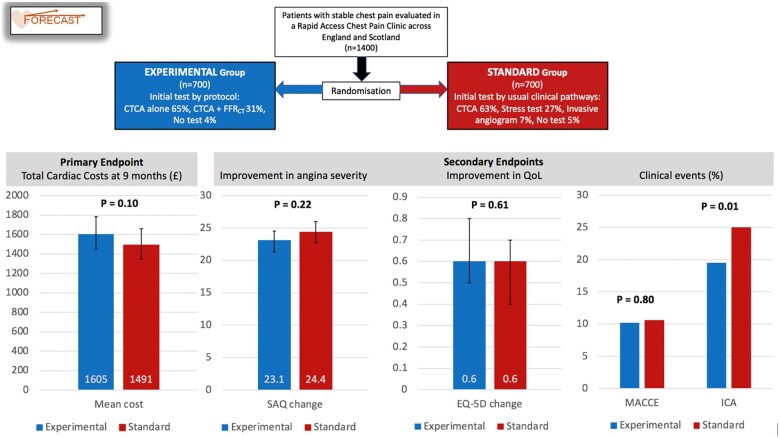
Summarising trial design and main results.

**See page 3853 for the editorial comment on this article (doi:10.1093/eurheartj/ehab538)**

## Introduction

The optimal approach to investigating patients who present with stable chest pain remains controversial. The majority of such patients in the UK are referred to a Rapid Access Chest Pain Clinic, which offers clinical assessment in a secondary care setting within 2 weeks of referral. Options for further testing have traditionally either assessed the coronary arteries for evidence of atheroma or used stress techniques to reveal reversible myocardial ischaemia.^1^ The role of invasive assessment and treatment in patients with stable chest pain is controversial, especially after the recent ISCHEMIA trial^2^ found that coronary revascularization did not improve prognosis when added to optimal medical therapy, despite its effectiveness in alleviating anginal symptoms. The addition of intracoronary pressure wire data, such as fractional flow reserve (FFR), to angiographic assessment has improved the management of patients with stable chest pain in both observational^3–8^ and randomized^9–12^ studies by identifying coronary lesions that are physiologically significant, which is poorly predicted by their angiographic appearance.^13^

The ideal test to assess patients with new onset chest pain might therefore simultaneously provide information about the extent of both coronary atheroma and myocardial ischaemia. Fractional flow reserve derived from computed tomography coronary angiography (FFR_CT_) is a well validated test^14–18^ that provides information about both the coronary atheroma burden, from a computed tomography coronary angiogram (CTCA), and assesses their functional importance using a computerized model of fluid dynamics based on the CTCA dataset.^14^ FFR_CT_ alters decision-making and patient management compared with CTCA data alone,^19^ and observational clinical studies, such as PLATFORM^20^ and ADVANCE,^21^ have demonstrated that the use of FFR_CT_ can reduce the requirement for invasive coronary angiography, without increasing ischaemic clinical events. Furthermore, the PLATFORM study suggested that the use of CTCA with FFR_CT_ reduces costs in the patients who would have undergone invasive coronary angiography, and is cost neutral in patients who would have had a non-invasive test.^22^ The UK National Institute for Health and Care Excellence (NICE) in 2017^23^ recommended that CTCA with FFR_CT_ be considered as a frontline test in patients with chest pain with the expectation of large cost savings.

The FORECAST trial was designed to test the hypothesis that, in a population of patients presenting to a Rapid Access Chest Pain Clinic, a strategy of using CTCA with selective FFR_CT_ would reduce total cardiac resource utilization and costs at 9 months, when compared with the standard clinical pathways based on NICE guidance.^24^ The secondary aims were to assess the effect of the experimental strategy on quality of life, angina status, subsequent clinical events, and the rate of invasive coronary angiography.

## Methods

### Trial design and oversight

FORECAST was an open-label, multicentre, randomized, controlled clinical trial. The rationale and design have previously been described,^25^ and the trial protocol is available in Supplementary material online, *Appendix* *SA*. The trial complies with the Declaration of Helsinki, was approved by the South Central Berkshire B Research Ethics Service Committee (REC Reference 18/SC/0490, IRAS Project ID: 231037) and is registered at ClinicalTrials.gov (NCT03187639). The trial was investigator-initiated and funded by an unrestricted research grant from HeartFlow^®^. The company had no role in the design or conduct of the trial, or in the data collection, analysis, or reporting. The trial steering committee oversaw the conduct of the trial, ensuring that: (i) it was conducted in a manner consistent with the protocol, (ii) the data were complete, and (iii) the analyses were performed according to the Statistical Analysis Plan.

### Patient population

All screened patients were at least 18 years old and were attending a Rapid Access Chest Pain Clinic for assessment of stable chest pain. A full list of exclusion criteria is available in the trial protocol (Supplementary material online, *Appendix* *SA*). In brief, patients were excluded if they had a history consistent with acute coronary syndrome, were deemed not to require a test to investigate their symptoms, were ineligible to undergo a CTCA, had a history of previous coronary revascularization, or had a life expectancy of <12 months.

### Randomization groups

Patients were randomized, using an independent computerized system with block sizes of two and four, to either the usual care strategy based on clinical pathways (standard group) or a strategy of CTCA with selective FFR_CT_ (experimental group). In the standard group, patients were assessed according to usual clinical care pathways at the Rapid Access Chest Pain Clinic, based upon the local implementation of the NICE CG95 Guidance for Chest Pain of Recent Onset.^24^ In these pathways, patients with a high pre-test likelihood of having important coronary disease could be referred for invasive coronary angiography, while patients with intermediate pre-test likelihood were referred for non-invasive evaluation, which could include stress testing (i.e. stress echocardiography, stress cardiac magnetic resonance, nuclear medicine perfusion imaging, and exercise electrocardiography), and CTCA (without FFR_CT_). In the experimental group, all patients were referred for CTCA as the initial test and selectively referred for FFR_CT_ if the CTCA demonstrated a stenosis of ≥40% in a coronary artery segment of diameter suitable for revascularization by either a coronary stent or coronary artery bypass graft surgery. Prior to randomization, the clinical team declared which initial test would be used in the event the patient was randomized to standard care. Subsequent clinical management was determined by the supervising physician based on the results of initial testing and clinical judgement.

### Trial endpoints

The primary endpoint was cardiovascular costs over 9 months of follow-up, calculated from the use of all cardiac-related invasive and non-invasive tests, revascularization procedures, hospital admissions and outpatient attendances due to a cardiovascular cause [including myocardial infarction (MI), arrhythmia, heart failure, revascularization], and cardiac medications. Data were collected using direct patient contact by research staff at each centre, as well as from local healthcare records. The total costs were calculated for each patient as the sum, over all specified resources, of the numbers of each resource used multiplied by a standardized cost weight (the UK tariffs, listed in the Supplementary material online, *Appendix* *SB*).

The two principal secondary endpoints were the changes in (i) quality of life, as assessed using the EQ-5D-5L questionnaire^26^ and (ii) angina status, as assessed using the Seattle Angina Questionnaire,^27^ which were completed at baseline and 9 months of follow-up. The other pre-specified secondary endpoints at 9 months of follow-up included major adverse cardiac and cerebrovascular events (MACCE), a composite of all-cause death, non-fatal MI, stroke, and cardiovascular hospitalization; the rate of invasive coronary angiography; and the rate of invasive angiography showing unobstructed coronaries (no stenosis of ≥50%).

### Statistical analysis

The sample size calculation and statistical analysis plan have been described in detail previously.^25^ Cost differences of 20% between the randomized groups were taken to be plausible and of importance for policy setting, since the PLATFORM economic substudy reported a 32% change in per-patient costs within the invasive stratum and 25% change within the non-invasive stratum.^22^ Based on the cost distributions in PLATFORM, we calculated that a sample size of 700 patients per group would provide 90% power to detect a 20% difference in costs between groups if there was no loss to follow-up, and 85% power with a loss to follow-up of up to 12%.

The Statistical Analysis Plan for the trial data was determined in advance (Supplementary material online, *Appendix* *SC*), conforms to the International Conference on Harmonization E9 guidelines, and is reported using the Consolidated Standards of Reporting Trials (CONSORT) guidelines. Categorical data are presented as counts and percentages, and continuous variables are presented as means and standard deviations, and medians and interquartile ranges. The analysis of the binary clinical outcomes was based on the frequency of the events and conducted using χ^2^ tests. The primary endpoint was compared using a two-sample *t*-test after a log transformation due to skew in the cost data. Confidence limits on mean costs were calculated by bootstrapping. A two-sided *P*-value of 0.05 or less was considered to constitute statistical significance for all analyses. All analyses of outcome data were conducted using an intention-to-treat framework.

## Results

**Figure 1:**
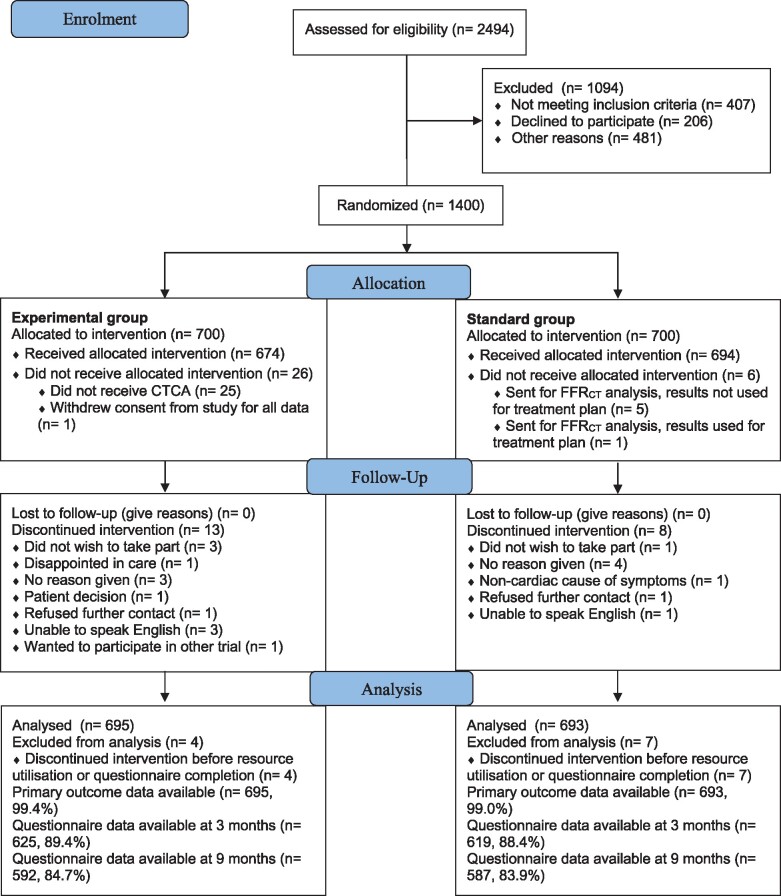
CONSORT diagram: flow of patients in the study, from screening to randomization and follow-up.

Between December 2017 and July 2019, 2494 patients with stable chest pain attending one of the 11 participating Rapid Access Chest Pain Clinics were screened for study entry, and 1400 patients were randomized (*Figure 1*). Baseline characteristics were well balanced between the arms (*Table 1*).

**Table 1:** Baseline characteristics

Baseline characteristics	Standard group	Experimental group
	(*n* = 700)	(*n* = 699)
Age, years, mean (SD)	59.6 (10.8)	60.0 (10.9)
Sex		
Male	364 (52.0)	359 (51.4)
Female	336 (48.0)	340 (48.6)
Ethnicity		
White	641 (91.6)	635 (90.8)
Black or Black British	11 (1.6)	10 (1.4)
Mixed	5 (0.7)	1 (0.1)
Asian or Asian British	32 (4.6)	47 (6.7)
Chinese or other ethnic group	11 (1.6)	4 (0.6)
Prefer not to answer	0 (0.0)	2 (0.3)
Smoking		
Never	319 (45.6)	348 (49.8)
Former	276 (39.4)	259 (37.1)
Current	104 (14.9)	92 (13.2)
Diabetes	86 (12.4)	91 (13.0)
Hypertension	234 (33.4)	266 (38.1)
Treated hyperlipidaemia	198 (28.3)	231 (33.1)
Family history of IHD	426 (60.9)	416 (59.5)
Previous MI	3 (0.4)	5 (0.7)

Values are *n* (%) unless otherwise specified.

IHD, ischaemic heart disease; MI, myocardial infarction; SD, standard deviation.

### Initial tests

Among the 700 patients randomized to the standard group, 439 (63%) had CTCA as the initial test, 187 (27%) had an initial stress test, and 47 (7%) had direct invasive coronary angiography (*Table 2*). Nine patients in the standard group were erroneously referred for FFR_CT_ analysis, but the test results were not used in clinical management.

**Table 2:** Initial tests undertaken

**Initial tests requested**	Standard group	Experimental group
	(*n* = 700)	(*n* = 699)
Non-invasive tests		
CTCA alone	430 (61.4)	454 (64.9)
FFR_CT_	9 (1.2)	220 (31.5)
Stress echo	103 (14.7)	0 (0.0)
Perfusion scan	13 (1.8)	0 (0.0)
Stress MRI	1 (0.1)	0 (0.0)
Exercise ECG	70 (10.0)	0 (0.0)
Invasive tests		
Coronary angiogram	47 (6.7)	0 (0.0)
No initial test done	36 (5.1)	25 (3.6)

Values are *n* (%).

CTCA, computed tomography coronary angiography; ECG, electrocardiogram; FFR_CT_, fractional flow reserve derived from computed tomography coronary angiography; MRI, magnetic resonance imaging.

In the experimental group, 674 (96%) patients underwent CTCA and 254 (38%) were selected for FFR_CT_ analysis by protocol because a lesion of ≥40% was seen in an epicardial coronary artery; five additional patients were also referred for FFR_CT_ who did not meet protocol criteria. Of the 259 patients referred for FFR_CT_, 39 (15%) scans could not be analysed due to technical issues. In the 220 patients who had FFR_CT_ performed, 126 patients (59%) had at least one epicardial vessel with an FFR_CT_ ≤ 0.8, which led to requests for invasive angiography in 98 patients, a non-invasive stress test in 16 patients, and no further testing in 12 patients. Invasive angiography was performed more often in patients with lower levels of FFR_CT_: in 26 of 29 patients (90%) with an FFR_CT_ between 0.50 and 0.60, in 23 of 23 patients (100%) with an FFR_CT_ between 0.61 and 0.70, in 39 of 56 patients (70%) with an FFR_CT_ between 0.71 and 0.80, and in 4 of 94 patients (4%) with an FFR_CT_ >0.80. The FFR_CT_ value was not recorded in 18 patients (although it is known that the value was <0.80), invasive angiography was performed in 14 (78%) of these patients.

### Tests and revascularization procedure at 9 months

Over 9 months of follow-up, fewer stress tests were performed at the discretion of the supervising physician in the experimental group than in the standard group (60 vs. 95, *Table 3*). The use of invasive coronary angiography was 22% lower in the experimental group (*Table 3*): 136 patients vs. 175 patients in standard care strategy (*P* = 0.01). The number of invasive angiograms showing no obstructive epicardial lesion was 52% lower in the experimental group: 30 patients vs. 62 patients in the standard care strategy. The use of invasive pressure wire assessment was also lower in the experimental group: 18 patients vs. 28 patients (*P* = 0.18).

**Table 3:** Components of the primary outcome: total cardiac costs at 9 months

Resource	Standard group	Experimental group	*P*-valuea
	(*n* = 700)	(*n* = 699)	
Non-invasive tests			
CTCA	462 (460)	690 (674)	<0.001
FFR_CT_	9 (9)	220 (220)	<0.001
Stress echo	124 (124)	13 (13)	<0.001
Perfusion scan	34 (34)	4 (4)	<0.001
Stress MRI	20 (20)	15 (15)	0.494
Exercise ECG	104 (99)	28 (27)	<0.001
Invasive procedures			
Coronary angiogram	182 (175)	156 (136)	0.014
FFR/iFR (invasive)^b^	28 (28)	18 (18)	0.177
PCI	75 (69)	88 (74)	0.660
CABG	28 (28)	28 (28)	1.000
Hospitalizations			
Myocardial infarction	3 (3)	10 (9)	0.091
Stroke	1 (1)	1 (1)	1.000
Transient ischaemic attack	2 (2)	2 (2)	1.000
Other	50 (46)	43 (35)	0.252
Emergency department visits	30 (27)	21 (20)	0.374
Cardiac outpatient visits	241 (156)	239 (139)	0.294
Total hospitalizations	327 (182)	316 (165)	0.322
Medications— months of prescription (patients)			
Statin	3279 (405)	3312 (410)	0.622
Aspirin	2379 (315)	2532 (331)	0.331
Antiplatelet^c^	771 (104)	744 (103)	1.000
Beta-blocker	1740 (238)	1728 (233)	0.910
Calcium blocker	1080 (152)	1422 (190)	0.015
Oral nitrate	360 (64)	486 (76)	0.285
ACE inhibitor	1245 (160)	1275 (169)	0.488
ARB	549 (75)	492 (73)	1.000
Alpha-blocker	120 (18)	192 (28)	0.135

Values are numbers of tests or events (numbers of patients) unless otherwise specified.

ACE, angiotensin-converting enzyme; ARB, angiotensin receptor blocker; CABG, coronary artery bypass graft; CTCA, computed tomography coronary angiography; ECG, electrocardiogram; FFR, fractional flow reserve; FFR_CT_, fractional flow reserve derived from computed tomography coronary angiography; iFR, instantaneous wave-free ratio; MRI, magnetic resonance imaging; PCI, percutaneous coronary intervention.

a Fisher’s exact test for the number of patients with one or more tests by group.

b Of the 28 invasive FFRs in the reference group, 16 were conducted as part of invasive coronary angiography and 12 were conducted as part of PCI. Of the 18 invasive FFRs in the test group, 12 were conducted as part of invasive coronary angiography and 6 were conducted as part of PCI. The counts of invasive coronary angiography and PCI are inclusive of these procedures, extra care was taken to ensure related costs were not double-counted.

c Clopidogrel, Ticagrelor, or Prasugrel.

The overall rate of coronary revascularization did not differ significantly between the groups: 15% in the experimental group vs. 14% in the standard group (*P* = 0.69). A total of 88 percutaneous coronary intervention (PCI) procedures were undertaken in 74 patients (11%) in the experimental group, compared with 75 PCIs in 69 patients (10%) in the standard group, with 28 patients in each group undergoing CABG surgery (*Table 3*). In the experimental group, 90 of the 102 patients who underwent coronary revascularization had a functional study (a stress test or FFR), compared with 49 of the 97 patients who underwent coronary revascularization in the standard group (*P* < 0.001).

### Primary endpoint: total cardiac costs at 9 months

**Figure 2:**
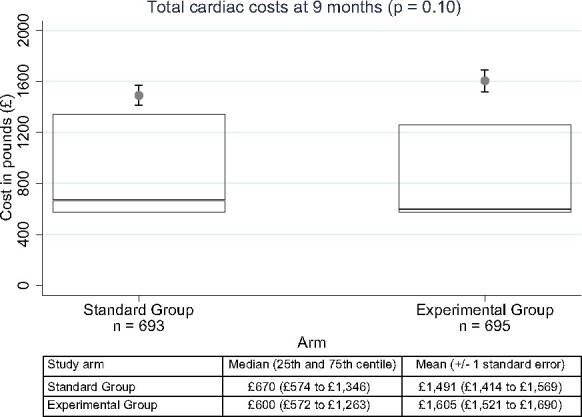
Primary endpoint: total cardiovascular costs at 9 months. Distribution of 9-month costs in UK pounds by randomized assignment. The top line of each box is the 75th percentile, the bottom line is the 25th percentile, and the line inside the box is the median (50th percentile). The mean cost is indicated by the filled circle, and one standard error of the mean is indicated by the error bars around the mean. The *P*-value (0.10) represents the result of the two-sample *t*-test applied to a log transformation of costs.

The mean total cardiac costs at 9 months were slightly higher in the experimental group (£1605) than in the standard group (£1491) [mean difference £114 (8%), 95% confidence interval of −£112 (−8%) to +£337 (+23%)], though the difference in mean costs was not significant (*P* = 0.10). The distribution of costs (*Figure 2*) was skewed upward by a minority of patients with high costs, such that the median costs were £70 lower in the experimental group than the standard group (£600 vs. £670).

The pattern of non-invasive test use varied significantly (by design) between the two randomized groups (*Table 3*), and while there was significantly lower use of invasive coronary angiography in the experimental group, the number of hospitalizations, visits to outpatient clinics and emergency departments, and medication use did not differ significantly (*Table 3*).

### Major adverse cardiac and cerebrovascular events at 9 months

The overall rate of MACCE (including death, non-fatal MI, non-fatal stroke, cardiovascular hospitalization) was 71 (10.2%) in the experimental group vs. 74 (10.6%) in the standard group (*P* = 0.80). Individual components of MACCE did not differ significantly between groups (*Table 4*). There were two deaths in the experimental group due to non-cardiac causes (metastatic cancer and progressive lung fibrosis).

**Table 4:** Major adverse cardiac and cerebrovascular events

Major adverse cardiac events	Standard group	Experimental group	***P*-value** ^a^
	(*n* = 700)	(*n* = 699)	
At least one major adverse cardiac event	74 (10.6)	71 (10.2)	0.799
Died from any cause	0 (0.0)	2 (0.3)	0.157
At least one hospitalization	74 (10.6)	69 (9.9)	0.666
At least one non-fatal MI	3 (0.4)	9 (1.3)	0.082
At least one non-fatal CVA	1 (0.1)	0 (0.0)	0.317

Values are *n* (%).

CVA, cerebrovascular accident; MI, myocardial infarction.

a The χ^2^ test for the number of patients with one or more tests by group.

### Quality of life and angina status

**Figure 3:**
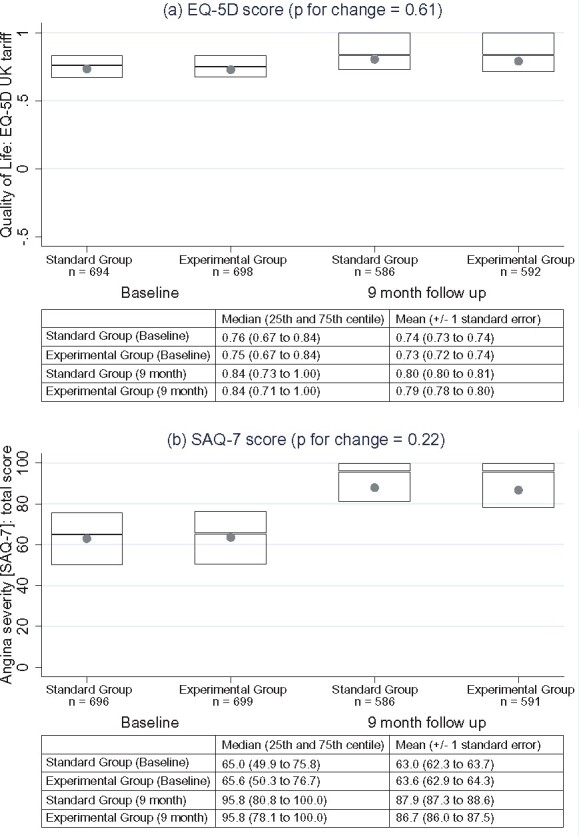
Principal secondary endpoints. Distribution of quality of life (*A*) and Seattle Angina Questionnaire scores (*B*) at baseline and 9 months. The boxes indicate the 75th percentile (top line), 25th percentile (bottom line), and 50th percentile (line within the box). The *P*-values, for changes in scores from baseline to 90 days, are based on the *t*-test. Note: higher Seattle Angina Questionnaire scores indicate lower angina severity.

Seattle Angina Questionnaire scores showed impairment at baseline (median score of 65 on a scale from 0 to 100 in both randomized groups) that improved significantly over 9 months of follow-up (to a median of 95.8 in both randomized groups). Scores improved to a similar degree in the experimental group (mean change 23.1, median change 23.3) and the standard group (mean change 25.0, median change 22.8), with no significant difference in the change in scores from baseline to 9 months (*P* = 0.22, *Figure 3*). The same pattern was evident in the EQ-5D scores over follow-up: both groups showed the reduced quality of life at baseline (median score 0.7 on a scale from 0 to 1 in both groups) that improved over follow-up (to a median score of 0.8 at 9 months in both groups), with no significant difference in the change in scores (0.1 in both groups, *P* = 0.61).

### Strata of planned initial test

Prior to randomization, the supervising clinician identified the test that would be performed in the event the patient was randomized to the standard care strategy. The pattern of costs varied depending on whether the planned test was an invasive angiogram, a stress test, or a CTCA (*Table 5*). The experimental group had 6.5% lower costs in the 94 patients with planned invasive angiography, 6.8% lower costs in the 393 patients with planned stress testing, but 20% higher costs in the 912 patients with planned CTCA (*Table 5*). The rates of MACCE did not differ between groups in any stratum, and the changes in Seattle Angina Questionnaire and EQ-5D scores were similar in all three strata. The effect of the experimental strategy on the use of invasive angiography was significantly greater (interaction *P* = 0.042) in the planned invasive angiography stratum than in the strata of planned CTCA and planned stress testing (*Table 5*).

**Table 5:** Trial endpoints by group and planned initial testing route

	**Invasive angiography**		**Stress test planned**		**CTCA planned**		
	Experimental group	Standard group	Experimental group	Standard group	Experimental group	Standard group	Interaction
	(*n* = 46)	(*n* = 48)	(*n* = 200)	(*n* = 193)	(*n* = 453)	(*n* = 459)	***P*-value** ^a^
Cost (UK pounds), mean (SD)	3702 (3246)	3958 (3313)	1297 (1592)	1392 (1812)	1527 (2220)	1272 (1777)	0.087
MACCE	15 (31)	15 (33)	15 (8)	12 (6)	44 (10)	44 (10)	0.663
Invasive angiography	29 (63)	46 (96)	29 (15)	43 (22)	78 (17)	86 (19)	0.042
SAQ change, mean (SD)	23 (21)	22 (20)	25 (19)	24 (19)	22 (20)	25 (21)	0.157
EQ-5D change, mean (SD)	0.03 (0.18)	0.08 (0.18)	0.04 (0.20)	0.07 (0.18)	0.07 (0.19)	0.06 (0.21)	0.064

Values are numbers of tests or events (numbers of patients) unless otherwise specified.

CTCA, computed tomography coronary angiography; EQ-5D, EuroQol five dimensions questionnaire; MACCE, major adverse cardiac and cerebrovascular event; SAQ, Seattle Angina Questionnaire; SD, standard deviation.

a Comparing between groups within patients in the CTCA planned strata vs. all other strata.

## Discussion

FORECAST is the first randomized trial to assess the strategy of CTCA with selective FFR_CT_ for the initial evaluation of patients presenting with stable chest pain. The main finding of the trial was that, in a low-risk population attending a Rapid Access Chest Pain Clinic, there was no significant difference in cost over 9 months between the experimental strategy and the standard strategy. Furthermore, there were no significant differences in symptoms, quality of life, MACCE, or use of coronary revascularization between the randomized groups. However, the experimental strategy led to a significant, 22% reduction in invasive coronary angiography, with 52% fewer patients having no significant obstructive coronary artery disease on invasive angiography .

FORECAST was designed using cost as the primary endpoint because we anticipated, based upon previous observational studies, that clinical outcomes would be similar in a well-managed population of stable patients with chest pain, irrespective of the initial testing strategy. We hypothesized that a strategy based on initial CTCA with selective FFR_CT_ would be more efficient, with lower resource use and cost. In 2017, the UK Medical Technologies Guidance on FFR_CT_ ^23^ predicted substantial cost savings for the National Health Service with the adoption of CTCA with FFR_CT_. Economic analysis of the observational PLATFORM study had shown cost savings from the use of FFR_CT_ when an invasive approach was planned, and cost neutrality when a non-invasive approach was planned.^22^ In FORECAST, we formally tested the hypothesis that there would be a meaningful cost saving from the experimental strategy based on FFR_CT_, but found no significant difference overall in costs compared with the standard care strategy. This negative result for the primary outcome might be due to the low prevalence of planned initial invasive angiography (7% of the trial population), and the high prevalence of CTCA as the planned initial test (65% of the trial population). This shift in standard practice in the UK towards routine CTCA, which was stimualted by the NICE CG95 Guidance on Chest Pain of Recent Onset, may have limited the cost savings potential of the experimental strategy based on initial CTCA and selective FFR_CT_.

Previous studies have consistently shown that the major benefit of FFR_CT_ has been to reduce the use of invasive coronary angiography, particularly angiograms showing no obstructed coronary arteries. In the observational PLATFORM study, invasive angiography was reduced by 61% in the FFR_CT_ cohort, and the clinical event rates at 1 year were equally low in both groups.^28^ The ADVANCE Registry of patients having CTCA and FFR_CT_ in routine clinical practice found unobstructed coronaries at invasive angiography in 14% of patients with FFR_CT_ ≤0.8, compared with 44% of patients with FFR_CT_ >0.8.^21^ In addition, there were no deaths or MIs within 90 days in the 1529 patients with FFR_CT_ >0.80, vs. 14 (0.3%) in subjects with an FFR_CT_ ≤0.80 (*P* = 0.039). We therefore anticipated that the experimental strategy in FORECAST would result in less invasive angiography, and no difference in clinical event rates, compared with standard clinical pathways. The results of FORECAST have confirmed these expectations, with equivalent rates of clinical events, 22% fewer invasive angiograms, and half the rate of unobstructed coronary arteries at invasive angiography in the experimental group, and are consistent with the previous observational studies.

In FORECAST, quality of life and angina status improved to a similar degree in both groups by 9 months of follow-up. This result is consistent with the 1-year data from PLATFORM, in which the five-item EuroQOL score did not differ significantly between the groups overall.^28^ The improvements seen in both groups are likely due to clinicians actively treating all subjects to relieve anginal symptoms, resulting in the similar use of anti-anginal medications and similar rates of coronary revascularization (*Table 3*). From a patient’s perspective, achieving similar quality of life and angina outcomes with fewer invasive procedures represents a potential advantage for the experimental strategy based on FFR_CT_.

There are some limitations of FORECAST. First, and most important, is that we could not anticipate the precise rate of use of CTCA in the standard group. The national guidelines were revised during planning of the trial, and while they recommended that CTCA become the default test for most patients attending Rapid Access Chest Pain Clinics, the infrastructure in many areas of the National Health Service at that time could not provide the test. The subsequent major expansion in CT facilities greatly improved access to CTCA in the last few years. The FORECAST trial, however, was based upon a pragmatic design: the experimental strategy (CTCA with selective FFR_CT_) vs. standard clinical care pathways, whatever tests that should include. With almost two-thirds of patients in the standard group having planned initial CTCA, the contrast between the randomized groups in FORECAST was diminished, along with the potential for cost savings with the experimental strategy based on the use of CTCA with selective FFR_CT_. A recent individual-based Markov microsimulation model for patients with low-risk stable chest pain, based upon the PROMISE population, suggested that an anatomical approach using CTCA was cost-effective compared with functional testing.^29^

A second limitation of the trial is that the costs in this study were based on UK National Health Service cost tariffs, and may not be generalizable to other countries with different cost structures in their health delivery systems. In an attempt to address this, one pre-specified sensitivity analysis for this trial is to apply US-specific cost tariffs to the FORECAST data, and this is the subject of ongoing analysis. Third, we used cardiac costs, rather than total medical costs, as the primary endpoint. Cardiac costs are more likely to be affected by the alternative strategies and were simpler for the local research teams to document. While it seems unlikely that non-cardiac costs would be affected by the management strategies tested, we cannot exclude the possibility that total medical costs differed, even though the cardiac costs did not.

The significant reduction in death from coronary heart disease and non-fatal MI seen at 5 years in the SCOT-HEART trial^30^ in the cohort undergoing CTCA, compared with routine care alone, indicates that there is considerable prognostic benefit from identifying coronary atheroma and initiating optimal medical therapy based on CTCA findings. Indeed, the results of FORECAST raise an important question that the trial cannot answer: namely, what is the optimal use of FFR_CT_ in routine clinical practice when CTCA is the default approach? In light of the findings from SCOT-HEART^30^ and ISCHEMIA,^2^ one could speculate that, rather than using FFR_CT_ based on the burden of atheroma found on CTCA, FFR_CT_ analysis could be reserved only for patients with insufficient symptomatic response to optimal medical therapy in whom revascularization is therefore being considered. This approach would be consistent with a sub-analysis of the PROMISE trial^31^ that demonstrated the value of describing degrees of coronary atheroma by CTCA in patients presenting with suspected angina, even in the absence of any functional testing for ischaemia, for predicting the primary endpoint of death, MI, and hospitalization for unstable angina. This suggests that the optimal application of FFR_CT_ in the setting of stable symptoms may be after optimal medical therapy fails to control angina adequately, at which time FFR_CT_ could be performed using the previously collected CTCA dataset, and thereby assess the need for revascularization as part of a shared decision-making process with the patient.

In conclusion, the experimental strategy of initial CTCA with selective FFR_CT_ in patients presenting with stable angina did not significantly reduce costs compared with standard clinical evaluation pathways, and led to similar clinical outcomes, including major adverse cardiovascular events, anginal symptoms, and quality of life. The experimental strategy based on FFR_CT_ did, however, reduce the use of invasive coronary angiography, without reducing the use of coronary revascularization.

## Supplementary material

Supplementary material is available at *European Heart Journal* online. 

## Supplementary Material

ehab444_Supplementary_Data
