# Supplementary material for: Fractional flow reserve derived from computed tomography coronary angiography in the assessment and management of stable chest pain: the FORECAST randomized trial
Source: Eur Heart J. 2021 Jul 16;42(37):3844–52. doi: 10.1093/eurheartj/ehab444 (PMC8648068; doi:10.1093/eurheartj/ehab444)
Supplement: ehab444_Supplementary_Data [file ehab444_supplementary_data.zip › ehab444-suppl_data/FORECAST APPENDIX C.docx]

**APPENDIX C**

Fractional Flow Reserve Derived from Computed Tomography Coronary Angiography in the Assessment and Management of Stable Chest Pain

Statistical Analysis Plan

| **Trial name:** | FORECAST |
| --- | --- |
| **Trial registration number:** | NCT03187639 |
| **Protocol title:** | Fractional Flow Reserve Derived from Computed Tomography Coronary Angiography in the Assessment and Management of Stable Chest Pain |
| **SAP version number:** | v1 |
| **SAP date:** | 27-August-2019 |

**To be approved and reviewed by:**

|  | **Name** | **Signature** | **Date** |
| --- | --- | --- | --- |
| **Chief Investigator** | Prof Nick Curzen |  |  |
| **Trial Statistician (author)** | Kayleigh Hill |  |  |
| **Senior Clinical Trial Statistician** | Tom Maishman |  |  |
| **Health Economist** | Mark Hlatky |  |  |

**Table of Contents**

[List of Abbreviations 4](#_Toc17811469)

[1 Introduction 5](#_Toc17811470)

[1.1 Purpose of SAP 5](#_Toc17811471)

[1.2 Trial background and rationale (short synopsis) 5](#_Toc17811472)

[1.3 Objectives 5](#_Toc17811473)

[1.4 Definition of endpoints 5](#_Toc17811474)

[1.5 Analysis principles 6](#_Toc17811475)

[2 Design considerations 7](#_Toc17811476)

[2.1 Description of trial design 7](#_Toc17811477)

[2.2 Trial power and sample size 7](#_Toc17811478)

[2.3 Randomisation details 7](#_Toc17811479)

[2.4 Timing of planned analyses 7](#_Toc17811480)

[3 Statistical considerations 8](#_Toc17811481)

[3.1 Definition of analysis populations 8](#_Toc17811482)

[3.2 Analysis software 8](#_Toc17811483)

[3.3 Methods for handling data 8](#_Toc17811484)

[3.4 Definition of key derived variables 9](#_Toc17811485)

[3.5 General principles for reporting and analysis 10](#_Toc17811486)

[4 Planned analyses and reporting 11](#_Toc17811487)

[4.1 Disposition of the study population (all patients) 11](#_Toc17811488)

[4.2 Protocol deviations (all patients) 11](#_Toc17811489)

[4.3 Baseline and demographic characteristics (ITT population, and upon request of CI, PP population) 11](#_Toc17811490)

[4.4 Treatment information (ITT population, and upon request of CI, PP population) 11](#_Toc17811491)

[4.5 Primary outcome (ITT population, and upon request of CI, PP population) 11](#_Toc17811492)

[4.6 Secondary outcome (ITT population, and upon request of CI, PP population) 12](#_Toc17811493)

[4.7 Safety reporting (ITT population, and upon request of CI, PP population) 13](#_Toc17811494)

[4.8 Other analyses (ITT population, and upon request of CI, PP population) 13](#_Toc17811495)

[5 Tables, listings and figures templates 14](#_Toc17811496)

[Disposition of the study population 16](#_Toc17811497)

[Follow-Up 16](file:///J:\Medicine\CTU\CTU%20STATISTICS%20AREA\01Trials\FORECAST\8%20-%20SAP\FORECAST%20SAP%20v1.docx#_Toc17811498)

[Analysis 16](file:///J:\Medicine\CTU\CTU%20STATISTICS%20AREA\01Trials\FORECAST\8%20-%20SAP\FORECAST%20SAP%20v1.docx#_Toc17811499)

[Enrollment 16](file:///J:\Medicine\CTU\CTU%20STATISTICS%20AREA\01Trials\FORECAST\8%20-%20SAP\FORECAST%20SAP%20v1.docx#_Toc17811500)

[Allocation 16](file:///J:\Medicine\CTU\CTU%20STATISTICS%20AREA\01Trials\FORECAST\8%20-%20SAP\FORECAST%20SAP%20v1.docx#_Toc17811501)

[Protocol deviations 18](#_Toc17811502)

[Baseline and demographic characteristics 19](#_Toc17811503)

[Treatment information 27](#_Toc17811504)

[Primary outcome analyses 37](#_Toc17811505)

[Secondary outcome analyses – secondary outcome 1 (clinical outcomes) 40](#_Toc17811506)

[Secondary outcome analyses – secondary outcome 2 (general wellbeing) 43](#_Toc17811507)

[Safety reporting 48](#_Toc17811508)

[6 References 49](#_Toc17811509)

[7 SAP revision history 49](#_Toc17811510)

## List of Abbreviations

| AE | Adverse Event |
| --- | --- |
| CRF | Case Report Form |
| CTCAE | Common Terminology Criteria for Adverse Events |
| DMEC | Data Monitoring and Ethics Committee |
| GCP | Good Clinical Practice |
| IDMC | Independent Data Monitoring Committee |
| ISF | Investigator Site File |
| MHRA | Medicines and Healthcare products Regulatory Agency |
| REC | Research Ethics Committee |
| SAE | Serious Adverse Event |
| SCTU | Southampton Clinical Trials Unit |
| TMF | Trial Master File |
| TMG | Trial Management Group |
| TSC | Trial Steering Committee |
| CP | Chest Pain |
| CAD | Coronary Artery Disease |
| RMI | Reversible Myocardial Ischaemia |
| MI | Myocardial Infarction |
| OMT | Optimal Medical Therapy |
| IC | Invasive Coronary Angiography |
| CTCA | CT Coronary Angiography |
| FFR | Fractional Flow Reserve |
| FFR_CT_ | Non-invasive technique using CT to determine Fractional Flow Reserve |
| QOL | Quality of Life |
| RACPC | Rapid Access Chest Pain Clinics |
| SAQ-7 | Seattle Angina Questionnaire – Short Form |
| EQ-5D-5L | Quality of life questionnaire |
| PCI | Percutaneous Coronary Inervention |
| CABG | Coronary Artery Bypass Graft |
| MRI | Magnetic Resonance Imaging |

**KEYWORDS**

Stable chest pain, CT coronary angiography, fractional flow reserve, coronary artery disease, reversible myocardial ischaemia, myocardial infarction

# Introduction

## Purpose of SAP

This statistical analysis plan (SAP) describes in detail the methods that will be used to analyse the data collected as part of the FORECAST trial. This will form the basis of the final trial publication. The final analysis will follow the SAP to ensure that the analyses are conducted in a scientifically valid manner and to avoid post hoc decisions which may affect the interpretation of the statistical analysis. Any deviations from the SAP will be detailed in the final report.

## Trial background and rationale (short synopsis)

This trial aims to test the hypothesis that FFRCT, used as the default screening tool for patients presenting with recent onset stable chest pain, would be associated with (i) shorter rapid time period between initial consultation and definitive management plan; (ii) better patient experience; (iii) lower overall use of resources.

The UK is well suited to test this hypothesis because of its well established system of Rapid Access Chest Pain Clinics (RACPC). The majority of patients presenting with stable CP that is of suspected cardiac origin are referred to such clinics, with a mandated access time within 2 weeks. The majority of such clinics work to the algorithm recommended in the NICE guidelines for Chest Pain of Recent Onset (March 2010). Within this guideline, patients are stratified according to their risk profile and pre-test likelihood of CAD to outcomes that include discharge, stress test (echo, MRI, nuclear), CT Coronary Angiography (CTCA), Ct coronary calcium score and invasive coronary angiography.

## Objectives

**Primary Objective:** To determine whether, in a population of patients presenting to RACPC, routine FFRCT as a default test is superior, in terms of resource utilisation at 9 months, when compared to routine clinical pathway algorithms recommended by NICE CG95.

**Secondary Objectives:**

1. To compare clinical outcomes between the two groups at 9 months

2. To compare the effect on general wellbeing between the two groups at 9 months

## Definition of endpoints

### Definition of primary endpoint

The primary endpoint is resource utilisation at 9 months, which is derived from:

- Requirement for non-invasive cardiac investigations
- Invasive angiography
- Revascularisation (including PCI & CABG)
- Hospitalisation for cardiac event (including MI/unstable angina/revascularisation/heart failure/arrhythmia)
- Cardiac medications

### Definition of secondary endpoints

The secondary endpoints are:

1. To compare clinical outcomes between the two groups at 9 months. Derived from:

- Major adverse cardiac events including: all cause mortality, hospitalization for cardiac event, non-fatal MI, and non-fatal CVA.
- Requirement for non-invasive cardiac investigations
- Invasive coronary angiography
- Revascularisation
- Procedural complications

1. To compare the effect on general wellbeing between the two groups at 9 months. Derived from:

- QoL questionnaire
- Patient satisfaction questionnaire
- Angina status
- Time to definitive management plan
- Time to completion of initial management
- Number of hospital attendances
- Working days lost

## Analysis principles

All analyses will be reported according to CONSORT 2010 and Southampton Clinical Trials Unit (SCTU) standard operating procedure (SOP) on planning, implementing and reporting statistical analyses (CTU/SOP/5058).

# Design considerations

## Description of trial design

FORECAST is a randomised controlled trial comparing 1400 patients with new onset chest pain who are prospectively assigned (using a 1:1 allocation) to either routine assessment (reference group) or FFRCT assessment (test group). Test interpretation and care decisions are to be made by the clinical care team.

Routine Assessment (Reference Group)

All patients in the ‘routine assessment’ group will be assessed according to the clinical algorithm used by the local Trust, which will be based on NICE guidelines for Chest Pain of Recent Onset (either the 2010 or 2016 version of CG 95, depending upon which version the local practice is following at the time of patient recruitment).

FFRCT (Test Group)

In the FFRCT group, all patients who are eligible for CTCA will undergo CTCA as their default test. In all cases the CTCA will be performed within the time frame that represents the standard waiting time for this test in that Trust. The results of the CTCA will determine whether the FFRCT will be performed.

## Trial power and sample size

A total of 1400 patients (700 per arm) will be recruited. See section 8.2 in the protocol for full details.

## Randomisation details

Patients will be randomised to either Routine Assessment group (Reference Group) or FFRCT group (Test Group) on a 1:1 ratio using varying block size randomisation, via an independent, web-based system (TENALEA). This online system allows for instant assignment to either the routine assessment or FFR_CT_ assessment, 24 hours per day. No stratification factors are incorporated in the randomisation.

## Timing of planned analyses

### Interim analyses and early stopping

N/A - no interim analyses are planned.

### Outline any stopping rules for the trial e.g. “The trial will be stopped if 50% or more of patients die prior to 6 months after randomisation”.

N/A - no stopping rules are in place.

### Final analysis

End of study is defined as when the last patient has had their last data collected, cleaned and verified.

# Statistical considerations

## Definition of analysis populations

### Intention-to-treat (ITT) analysis population

This population includes all patients that were randomised regardless of treatment compliance. All summaries and analysis will be on the ITT population unless otherwise specified.

### Per-protocol analysis population

This population is formed of the ITT population, but excludes patients who have ‘crossed’ randomisation, (i.e. patients in the FFRCT group who did not have a FFRCT; or patients in the routine group who had a FFRCT) and patients who have experienced any major protocol violations, including but not limited to:

- FFRCT arm patient has CAD ≥40% but not referred for FFRCT;
- FFRCT negative patient undergoes ICA;
- FFRCT positive patient undergoes invasive pressure wire;
- Patient being managed without FFRCT data taken into account.

## Analysis software

SAS v9.4 or higher, or Stata v15.1 or higher will be used for all analyses.

## Methods for handling data

### Withdrawal from trial

All data up until the point of patient withdrawal from the trial will be used in analyses unless the patient withdrew consent and does not wish for the data already collected prior to withdrawal to be used for the trial.

### Missing data

Missing data for this trial is not anticipated to be high. However, if the amount of missing data at 9 months is deemed too high, the reasons for the missing data will be explored, and methods such as multiple imputation, adjusting for both baseline and 3 month data, will be carried out to handle the missing data if/where appropriate. If multiple imputation is incorporated, this will act as a sensitivity analysis.

### Outliers

No methods will be used to handle outliers in the data.

### Assumption checking and alternative methods

This analysis uses regression modelling, of which one of the assumptions is normally distributed residuals. If this assumption isn’t met for a particular model then an appropriate transformation will be considered. For example:

- Log - $\ln\left( y+c \right)$

If a suitable transformation cannot be found then non-parametric analysis, i.e. Mann-Whitney tests, or negative binomial regression if the data is zero-inflated, will be performed.

For Cox proportional hazards models, if the proportional hazards assumption does not hold, other appropriate regression methods will be explored e.g. stratified Cox model, time-varying Cox model, flexible parametric model, etc. Proportionality will be assessed by e.g. plotting the Schoenfeld residuals, etc.

### Data transformations

Any data transformations which will be considered during the analysis of the data have been described in detail in section 3.3.4 above as part of the statistical models assumption checking and alternative methods. No further transformations will be performed.

### HES Data

Clinical trial data will be supplemented with the use of HES data. This is in order to help capture events which otherwise might have been missed by the clinical trial data collection. Any event captured by either the clinical trial data or the HES data will be incorporated in the analyses.

## Definition of key derived variables

Detailed information on how the cost is derived can be found in Section 4.5. The following variables below are provided in order to outline how these key variables should be derived for the analyses.

*Key time to management plan variables*

- Time to definitive management plan = time from randomisation until the time at which a definitive management has been established.  Definitive management is defined as choice to revascularise (i.e. CABG or PCI) or to OMT/discharge.  It should be noted that if the patient needs another test after initial test (e.g. stress MRI or ICA) then this is not the definitive management plan.
- Time to completion of definitive management plan = time from randomisation until the time of completion of definitive management (i.e. the patient has received revascularisation or been discharged on OMT).

*Key time to event variables*

- Time to first major adverse cardiac event = Time from date of randomisation to date of first major adverse cardiac event. Patients who do not experience an event will be censored at the date of the last follow-up. Otherwise, all other non-major adverse cardiac events will be censored at the date of the ‘non-event’.
- Time to first requirement for non-invasive cardiac investigations = Time from date of randomisation to date of first non-invasive cardiac investigations event. Patients who do not experience an event will be censored at the date of the last follow-up. Otherwise, all other events will be censored at the date of the ‘non-event’.
- Time to first invasive coronary angiography = Time from date of randomisation to date of first invasive coronary angiography event. Patients who do not experience an event will be censored at the date of the last follow-up. Otherwise, all other events will be censored at the date of the ‘non-event’.
- Time to first revascularisation = Time from date of randomisation to date of first revascularisation event. Patients who do not experience an event will be censored at the date of the last follow-up. Otherwise, all other events will be censored at the date of the ‘non-event’.
- Time to first procedural complication = Time from date of randomisation to date of first procedural complication event. Patients who do not experience an event will be censored at the date of the last follow-up. Otherwise, all other events will be censored at the date of the ‘non-event’.

## General principles for reporting and analysis

The following general principles for reporting and analysis will be used:

- - 5% two-sided level of statistical significance, with corresponding 95% confidence intervals presented where applicable.
  - No adjustments for multiplicity are planned.
  - Summary statistics will include either mean, standard deviation, and range and/or median, interquartile range, and range.
  - Treatment groups will be labelled in the tables as Reference Group and Test Group accordingly, and a total column will be included in tables were applicable.

# Planned analyses and reporting

## Disposition of the study population (all patients)

A CONSORT diagram will be produced showing a clear account of all patients who entered the trial (an example is shown at this link http://www.consort-statement.org/consort-statement/flow-diagram0/) - see Section 5, Figure 1 for an example figure. Withdrawal information including the primary reasons of discontinuation will be summarised and presented by group – see Section 5, Table 1 and Table 2 for example tables.

## Protocol deviations (all patients)

A listing of all Major or Potential/Serious Breach (Major protocol deviations with potential to affect patient safety/data) and Potential/Serious Breach (with actual affect to patient safety/data, Major/Potential/Serious Breach of GCP guidelines or consistent non-compliance by site) will be produced (by patient and site where applicable) – see Section 5, Table 3 for an example table.

## Baseline and demographic characteristics (ITT population, and upon request of CI, PP population)

Summary statistics will be produced and presented by group for demographic and baseline characteristics but no comparisons will be undertaken, rather the clinical importance of any imbalance will be noted – see Section 5, Tables 4 to 11 for example tables.

## Treatment information (ITT population, and upon request of CI, PP population)

Summary statistics will be produced and presented by group for treatment information but no comparisons will be undertaken, rather the clinical importance of any imbalance will be noted – see Section 5, Tables 12 to 15 for example tables.

## Primary outcome (ITT population, and upon request of CI, PP population)

The primary endpoint is resource utilisation at 9 months. Total medical costs over nine months of follow-up will be measured for each patient as the weighted sum of the numbers of key medical resources used between study entry and the nine month follow-up point.

C_i_ = Σ_ϳ_ ω_ϳ_ * N_iϳ_

where C_i_ = the cost for patient “i”, ω_ϳ =_ the cost weight for resource “j”, and N_iϳ_ = the number of units of resource ”j” used by patient ”I”.

In describing the results, we will first enumerate the use of key resources in each of the “j” categories, or at least the categories with either large cost-weights ω_ϳ_ or large category costs N_ϳ_ = Σ_í_ N_iϳ_. See Section 5, Table 16 for an example table of these descriptive statistics, which will show the readers the underlying determinants of the summary cost measures.

The total medical costs over follow-up will be compared between randomly assigned groups in an intention-to-treat fashion (See Section 5, Table 17 for an example table). The mean and median cost should be presented using a figure with a bar-and-whisker or cumulative frequency distribution to contrast visually the patterns of costs in the two randomly assigned groups (See Section 5, Figure 2 for an example figure). The distribution of C_i_ will be right-skewed, so non-parametric statistical tests, i.e. Mann-Whitney tests, or negative binomial regression, will be needed to test the hypotheses that nine month costs differ between groups. The initial costs will differ by design (i.e., FFR_CT_ vs. usual care tests) so statistical tests of different initial costs are not of particular interest. The primary hypothesis is the total costs differ between groups, but we will also compare follow-up costs differ between groups to test the hypothesis that assignment to the FFR_CT_ group will reduce the use of follow-up tests and events, and hence lower subsequent costs of care. This will allow us to estimate the cost savings from FFR_CT_, if any.

Finally, we can use of regression model to assess the effect of baseline factors, along with assigned testing strategy, upon total nine month costs. The model will use log (cost) as the dependent variables to minimize the effect of skewed costs (we may need to add a small constant to all Ci avoid distortions due to log (0)). We can control for the test that was identified a priori to be used if the patient were assigned to usual care. We can particularly test for an interaction between invasive coronary angiography as the identified first test and random assignment to the FFR_CT_ strategy (suggested by PLATFORM). See Section 5, Table 18 for an example table.

## Secondary outcome (ITT population, and upon request of CI, PP population)

The secondary outcomes are:

1. To compare clinical outcomes between the two groups at 9 months. Derived from:

- Major adverse cardiac events including: all cause mortality, hospitalization for cardiac event, non-fatal MI, and non-fatal CVA.
- Requirement for non-invasive cardiac investigations
- Invasive coronary angiography
- Revascularisation
- Procedural complications

1. To compare the effect on general wellbeing between the two groups at 9 months. Derived from:

- QoL questionnaire
- Patient satisfaction questionnaire
- Angina status
- Time to definitive management plan
- Time to completion of initial management
- Number of hospital attendances
- Working days lost

### Secondary outcome 1 (clinical outcomes) (ITT population, and upon request of CI, PP population)

For the comparison of clinical outcomes between the two groups at 9 months. The number and proportion of experiencing at least one event (Major adverse cardiac events; Requirement for non-invasive cardiac investigations; Requirement for invasive coronary angiography; Revascularisation; and Procedural complications) will be reported and presented by group (and formally compared by group using Pearson Chi-squared tests), along with the number experiencing more than one event by group, and the mean or median number of events by group.

Time to the first clinical event (Major adverse cardiac events; Requirement for non-invasive cardiac investigations; etc.) will be described using Kaplan Meier plots, presented by arm, and analysed using Cox regression modelling, both unadjusted and adjusted for site provided sufficient numbers in the each stratum. See Section 3.4 for definition of the time to event variables. Upon request of the CI, time to first individual major adverse cardiac event (death from any cause, hospitalisation for a cardiac event, non-fatal MI, and non-fatal CVA) will be individually analysed in a similar manner (with ‘non-events’ including both other clinical outcomes as well as the other associated major adverse cardiac events which are not the focus of the analysis). See Section 5, Tables 19 to 28, Figures 3 to 7 for example tables and figures.

### Secondary outcome 2 (general wellbeing) (ITT population, and upon request of CI, PP population)

For the questionnaire patient reported endpoints (EQ-5D QoL questionnaire, Patient Satisfaction questionnaire and Seattle Angina questionnaire) at 9 months, change scores from baseline to 9 months will be presented and mean (or median) change from baseline to 9 months will be compared between groups using t-tests (or the Wilcoxon rank-sum test), where appropriate. See Section 5, Tables 29 to 31 for example tables. A summary of these changes may be presented by way of a box and whisker plot (see Section 5, Figure 8 for an example figure).

For time to definitive management plan and time to completion of initial management endpoints summary statistics will be used to describe the data, with box and whisker plots, along with a formal comparison by group using a Mann-Whiney U test, or negative binomial regression if the data is zero-inflated. See Section 5, Figure 9, Figure 10, Table 32 and Table 33 for example tables and figures.

For number of hospital attendances and the number of working days lost, means and standard deviations will be presented and analysed by group using ANCOVA (if data is skewed, medians and ranges will be presented and analysis will be by Mann Whitney tests, or negative binomial regression if the data is zero-inflated). These will be adjusted for site provided sufficient numbers in the each stratum. See Section 5, Table 34, Table 35, Table 36, and Table 37 for example tables.

## Safety reporting (ITT population, and upon request of CI, PP population)

CTCA related serious adverse events will be summarised for the Test group only (see Section 5, Table 38). Mortality information will be summarised by group (see Section 5, Table 39). A listing of serious adverse events (SAEs) will be provided for all related/unrelated SAEs (see Section 5, Table 40). If required, a summary table will also be presented.

## Other analyses (ITT population, and upon request of CI, PP population)

If differences between costs are observed between groups, exploratory analyses into costs to determine what the drivers are for the costs per group will be carried out via adjusted regression models. Example tables and figures not shown.

# Tables, listings and figures templates

Figure Contents

[Figure 1 – CONSORT Diagram 16](#_Toc17809895)

[Figure 2 – Box and whisker plot for total medical costs over nine months, by Group 39](#_Toc17809896)

[Figure 3 – Kaplan Meier Plot for time to first major adverse cardiac event 41](#_Toc17809897)

[Figure 4 – Kaplan Meier Plot for time to first requirement for non-invasive cardiac investigations event 42](#_Toc17809898)

[Figure 5 – Kaplan Meier Plot for time to first invasive coronary angiography event 42](#_Toc17809899)

[Figure 6 – Kaplan Meier Plot for time to first revascularisation event 42](#_Toc17809900)

[Figure 7 – Kaplan Meier Plot for time to first procedural complication event 42](#_Toc17809901)

[Figure 8 - Change in QoL scores from baseline to 9 months summary 45](#_Toc17809902)

[Figure 9 – Box and whisker plot for time to definitive management plan, by group 46](#_Toc17809903)

[Figure 10 – Box and whisker plot for time to completion of initial management, by group 46](#_Toc17809904)

Table Contents

[Table 1 – Withdrawal from CTCA information 17](#_Toc17809905)

[Table 2 – Withdrawal from trial information 18](#_Toc17809906)

[Table 3 – List of Major protocol deviations 18](#_Toc17809907)

[Table 4 – Subject Characteristics 19](#_Toc17809908)

[Table 5 – Demographics and Medical History 20](#_Toc17809909)

[Table 6 – EQ-5D-5L questionnaire 22](#_Toc17809910)

[Table 7 – Health today questionnaire^1^ 23](#_Toc17809911)

[Table 8 – Seattle Angina – 7 24](#_Toc17809912)

[Table 9 – Diamond Forrester Risk Score 25](#_Toc17809913)

[Table 10 – First Choice Test 26](#_Toc17809914)

[Table 11 – Routine Investigations Referral (Reference Group only) 26](#_Toc17809915)

[Table 12 – CTCA Data (Test group only) 27](#_Toc17809916)

[Table 13 – CTCA Results (Test group only) 29](#_Toc17809917)

[Table 14 – FFR_CT_ Results (Test group only) 35](#_Toc17809918)

[Table 15 – Final Treatment Plan 37](#_Toc17809919)

[Table 16 – Resource use over nine months, by group 37](#_Toc17809920)

[Table 17 – Total medical costs over nine months, by group 38](#_Toc17809921)

[Table 18 – Factors affecting total costs, in a log linear model 40](#_Toc17809922)

[Table 19 – Major adverse cardiac events at 9 months summary 40](#_Toc17809923)

[Table 20 – Cox regression model results for time to first major adverse cardiac events 41](#_Toc17809924)

[Table 21 – Requirement for non-invasive cardiac investigations summary 42](#_Toc17809925)

[Table 22 – Cox regression model results for time to first requirement for non-invasive cardiac investigations 42](#_Toc17809926)

[Table 23 – Invasive coronary angiography summary 42](#_Toc17809927)

[Table 24 – Cox regression model results for time to first invasive coronary angiography 42](#_Toc17809928)

[Table 25 – Revascularisation summary 42](#_Toc17809929)

[Table 26 – Cox regression model results for time to first revascularisations 42](#_Toc17809930)

[Table 27 – Procedural complications summary 42](#_Toc17809931)

[Table 28 – Cox regression model results for time to first procedural complications 43](#_Toc17809932)

[Table 29 – Change in EQ-5D-5L scores from baseline to 9 months 43](#_Toc17809933)

[Table 30 – Change in patient satisfaction from baseline to 9 months 44](#_Toc17809934)

[Table 31 – Change in Seattle Angina from baseline to 9 months 44](#_Toc17809935)

[Table 32 –Time to definitive management plan summary 46](#_Toc17809936)

[Table 33 – Time to completion of initial management summary 46](#_Toc17809937)

[Table 34 – Number of hospital attendances summary 47](#_Toc17809938)

[Table 35 – Number of hospital attendances ANCOVA 47](#_Toc17809939)

[Table 36 – Number of working days lost summary (for those who are employed at baseline only) 47](#_Toc17809940)

[Table 37 – Number of working days lost ANCOVA (for those who are employed at baseline only) 47](#_Toc17809941)

[Table 38 – CTCA Related Serious Adverse Events (Test group only) 48](#_Toc17809942)

[Table 39 – Mortality information 48](#_Toc17809943)

[Table 40 – SAE Listings 48](#_Toc17809944)

## Disposition of the study population

Figure 1 – CONSORT Diagram

## Follow-Up

Analysed (n= )
♦ Excluded from analysis (give reasons) (n= )

## Analysis

Analysed (n= )
♦ Excluded from analysis (give reasons) (n= )

Lost to follow-up (give reasons) (n= )

Discontinued intervention (give reasons) (n= )

Lost to follow-up (give reasons) (n= )

Discontinued intervention (give reasons) (n= )

## Enrollment

Allocated to intervention (n= )

♦ Received allocated intervention (n= )

♦ Did not receive allocated intervention (give reasons) (n= )

## Allocation

Allocated to intervention (n= )

♦ Received allocated intervention (n= )

♦ Did not receive allocated intervention (give reasons) (n= )

Randomized (n= )

Excluded (n= )

♦  Not meeting inclusion criteria (n= )

♦  Declined to participate (n= )

♦  Other reasons (n= )

Assessed for eligibility (n= )

Table 1 – Withdrawal from CTCA information

| **Characteristic** | **Reference Group**  **(n=XXX)** | **Test Group**  **(n=XXX)** | **Total**  **(n=XXX)** |
| --- | --- | --- | --- |
|  |  |  |  |
| **Withdrawal from CTCA test – n (%)^1^** |  |  |  |
| Yes | N/A | xx (xx.x%) | N/A |
| No | N/A | xx (xx.x%) | N/A |
| *Missing from eCRF – n (%)^2^* | *N/A* | *xx (xx.x%)* | *N/A* |
|  |  |  |  |
| **Received FFR_CT_ – n (%)^1^** |  |  |  |
| Yes | xx (xx.x%) | xx (xx.x%) | xx (xx.x%) |
| Angiogram performed: |  |  |  |
| Yes | xx (xx.x%) | xx (xx.x%) | xx (xx.x%) |
| No | xx (xx.x%) | xx (xx.x%) | xx (xx.x%) |
| No | xx (xx.x%) | xx (xx.x%) | xx (xx.x%) |
| Angiogram performed: |  |  |  |
| Yes | xx (xx.x%) | xx (xx.x%) | xx (xx.x%) |
| No | xx (xx.x%) | xx (xx.x%) | xx (xx.x%) |
| *Missing from eCRF – n (%)^2^* | *xx (xx.x%)* | *xx (xx.x%)* | *xx (xx.x%)* |
|  |  |  |  |

^1^ Denominator is the number of patients with non-missing information available.

^2^ Denominator is the number of patients

Table 2 – Withdrawal from trial information

| **Characteristic** | **Reference Group**  **(n=XXX)** | **Test Group**  **(n=XXX)** | **Total**  **(n=XXX)** |
| --- | --- | --- | --- |
|  |  |  |  |
| **Completed study – n (%)^1^** | xx (xx.x%) | xx (xx.x%) | xx (xx.x%) |
| **Prematurely withdrew from study** **– n (%)^1^** | xx (xx.x%) | xx (xx.x%) | xx (xx.x%) |
| Reason: |  |  |  |
| Death | xx (xx.x%) | xx (xx.x%) | xx (xx.x%) |
| XXXX | xx (xx.x%) | xx (xx.x%) | xx (xx.x%) |
| XXXX | xx (xx.x%) | xx (xx.x%) | xx (xx.x%) |
| … | … | … | … |
| Lost to follow Up | xx (xx.x%) | xx (xx.x%) | xx (xx.x%) |
| Other: | xx (xx.x%) | xx (xx.x%) | xx (xx.x%) |
| XXXX | xx (xx.x%) | xx (xx.x%) | xx (xx.x%) |
| XXXX | xx (xx.x%) | xx (xx.x%) | xx (xx.x%) |
| … | … | … | … |
| Physician decision: | xx (xx.x%) | xx (xx.x%) | xx (xx.x%) |
| XXXX | xx (xx.x%) | xx (xx.x%) | xx (xx.x%) |
| XXXX | xx (xx.x%) | xx (xx.x%) | xx (xx.x%) |
| … | … | … | … |
| Protocol violation | xx (xx.x%) | xx (xx.x%) | xx (xx.x%) |
| Screen failure | xx (xx.x%) | xx (xx.x%) | xx (xx.x%) |
| Study terminated by sponsor | xx (xx.x%) | xx (xx.x%) | xx (xx.x%) |
| Withdrawal by subject: | xx (xx.x%) | xx (xx.x%) | xx (xx.x%) |
| XXXX | xx (xx.x%) | xx (xx.x%) | xx (xx.x%) |
| XXXX | xx (xx.x%) | xx (xx.x%) | xx (xx.x%) |
| … | … | … | … |
| *Missing from eCRF – n (%)^2^* | *xx (xx.x%)* | *xx (xx.x%)* | *xx (xx.x%)* |
|  |  |  |  |

^1^ Denominator is the number of patients with non-missing information available.

^2^ Denominator is the number of patients

## Protocol deviations

Table 3 – List of Major protocol deviations

*[Example table not shown]*

## Baseline and demographic characteristics

Table 4 – Subject Characteristics

| **Characteristic** | **Reference Group**  **(n=XXX)** | **Test Group**  **(n=XXX)** | **Total**  **(n=XXX)** |
| --- | --- | --- | --- |
|  |  |  |  |
| **Age at Baseline – years^1^** |  |  |  |
| Mean | xx.x | xx.x | xx.x |
| Standard deviation | xx.xx | xx.xx | xx.xx |
| Median | xx | xx | xx |
| IQR | xx to xx | xx to xx | xx to xx |
| Range | xx to xx | xx to xx | xx to xx |
| *Missing from eCRF – n (%)^2^* | *xx (xx.x%)* | *xx (xx.x%)* | *xx (xx.x%)* |
|  |  |  |  |
|  |  |  |  |
| **Height – cm^1^** |  |  |  |
| Mean | xx.x | xx.x | xx.x |
| Standard deviation | xx.xx | xx.xx | xx.xx |
| Median | xx | xx | xx |
| IQR | xx to xx | xx to xx | xx to xx |
| Range | xx to xx | xx to xx | xx to xx |
| *Missing from eCRF – n (%)^2^* | *xx (xx.x%)* | *xx (xx.x%)* | *xx (xx.x%)* |
|  |  |  |  |
|  |  |  |  |
| **Weight – kg^1^** |  |  |  |
| Mean | xx.x | xx.x | xx.x |
| Standard deviation | xx.xx | xx.xx | xx.xx |
| Median | xx | xx | xx |
| IQR | xx to xx | xx to xx | xx to xx |
| Range | xx to xx | xx to xx | xx to xx |
| *Missing from eCRF – n (%)^2^* | *xx (xx.x%)* | *xx (xx.x%)* | *xx (xx.x%)* |
|  |  |  |  |
|  |  |  |  |
| **BMI – kg/m^2 1^** |  |  |  |
| Mean | xx.x | xx.x | xx.x |
| Standard deviation | xx.xx | xx.xx | xx.xx |
| Median | xx | xx | xx |
| IQR | xx to xx | xx to xx | xx to xx |
| Range | xx to xx | xx to xx | xx to xx |
| *Missing from eCRF – n (%)^2^* | *xx (xx.x%)* | *xx (xx.x%)* | *xx (xx.x%)* |
|  |  |  |  |
|  |  |  |  |
| **Gender – n (%)^1^** |  |  |  |
| Male | xx (xx.x%) | xx (xx.x%) | xx (xx.x%) |
| Female | xx (xx.x%) | xx (xx.x%) | xx (xx.x%) |
| *Missing from eCRF – n (%)^2^* | *xx (xx.x%)* | *xx (xx.x%)* | *xx (xx.x%)* |
|  |  |  |  |

^1^ Denominator is the number of patients with non-missing information available.

^2^ Denominator is the number of patients

Table 5 – Demographics and Medical History

| **Characteristic** | **Reference Group**  **(n=XXX)** | **Test Group**  **(n=XXX)** | **Total**  **(n=XXX)** |
| --- | --- | --- | --- |
|  |  |  |  |
| **Ethnicity – n (%)^1^** |  |  |  |
| White | xx (xx.x%) | xx (xx.x%) | xx (xx.x%) |
| Black or Black British | xx (xx.x%) | xx (xx.x%) | xx (xx.x%) |
| Mixed | xx (xx.x%) | xx (xx.x%) | xx (xx.x%) |
| Asian or Asian British | xx (xx.x%) | xx (xx.x%) | xx (xx.x%) |
| Chinese or Other Ethnic Group | xx (xx.x%) | xx (xx.x%) | xx (xx.x%) |
| Prefer not to answer | xx (xx.x%) | xx (xx.x%) | xx (xx.x%) |
| *Missing from eCRF – n (%)^2^* | *xx (xx.x%)* | *xx (xx.x%)* | *xx (xx.x%)* |
|  |  |  |  |
|  |  |  |  |
| **Employment Status – n (%)^1^** |  |  |  |
| Working full-time (30 hours or more a week) | xx (xx.x%) | xx (xx.x%) | xx (xx.x%) |
| Working part-time (less tan 30 hours) | xx (xx.x%) | xx (xx.x%) | xx (xx.x%) |
| Not currently employed | xx (xx.x%) | xx (xx.x%) | xx (xx.x%) |
| *Missing from eCRF – n (%)^2^* | *xx (xx.x%)* | *xx (xx.x%)* | *xx (xx.x%)* |
|  |  |  |  |
|  |  |  |  |
| MEDICAL HISTORY |  |  |  |
|  |  |  |  |
| **Angina – n (%)^1^** |  |  |  |
| Yes | xx (xx.x%) | xx (xx.x%) | xx (xx.x%) |
| Typical Angina: |  |  |  |
| Yes | xx (xx.x%) | xx (xx.x%) | xx (xx.x%) |
| No | xx (xx.x%) | xx (xx.x%) | xx (xx.x%) |
| CCS Class: |  |  |  |
| No Angina | xx (xx.x%) | xx (xx.x%) | xx (xx.x%) |
| Class I | xx (xx.x%) | xx (xx.x%) | xx (xx.x%) |
| Class II | xx (xx.x%) | xx (xx.x%) | xx (xx.x%) |
| Class III | xx (xx.x%) | xx (xx.x%) | xx (xx.x%) |
| Class IV | xx (xx.x%) | xx (xx.x%) | xx (xx.x%) |
| No | xx (xx.x%) | xx (xx.x%) | xx (xx.x%) |
| *Missing from eCRF – n (%)^2^* | *xx (xx.x%)* | *xx (xx.x%)* | *xx (xx.x%)* |
|  |  |  |  |
|  |  |  |  |
| **Diabetes – n (%)^1^** |  |  |  |
| Yes | xx (xx.x%) | xx (xx.x%) | xx (xx.x%) |
| Type: |  |  |  |
| Type I | xx (xx.x%) | xx (xx.x%) | xx (xx.x%) |
| Type II | xx (xx.x%) | xx (xx.x%) | xx (xx.x%) |
| Type II – Treated with drugs: |  |  |  |
| Yes | xx (xx.x%) | xx (xx.x%) | xx (xx.x%) |
| No | xx (xx.x%) | xx (xx.x%) | xx (xx.x%) |
| Type II – Treated with insulin (+/- drugs): |  |  |  |
| Yes | xx (xx.x%) | xx (xx.x%) | xx (xx.x%) |
| No | xx (xx.x%) | xx (xx.x%) | xx (xx.x%) |
| No | xx (xx.x%) | xx (xx.x%) | xx (xx.x%) |
| *Missing from eCRF – n (%)^2^* | *xx (xx.x%)* | *xx (xx.x%)* | *xx (xx.x%)* |
|  |  |  |  |
|  |  |  |  |
| **Treated hypertension – n (%)^1^** |  |  |  |
| Yes | xx (xx.x%) | xx (xx.x%) | xx (xx.x%) |
| No | xx (xx.x%) | xx (xx.x%) | xx (xx.x%) |
| *Missing from eCRF – n (%)^2^* | *xx (xx.x%)* | *xx (xx.x%)* | *xx (xx.x%)* |
|  |  |  |  |
|  |  |  |  |
| **Treated hyperlipidaemia – n (%)^1^** |  |  |  |
| Yes | xx (xx.x%) | xx (xx.x%) | xx (xx.x%) |
| No | xx (xx.x%) | xx (xx.x%) | xx (xx.x%) |
| *Missing from eCRF – n (%)^2^* | *xx (xx.x%)* | *xx (xx.x%)* | *xx (xx.x%)* |
|  |  |  |  |
|  |  |  |  |
| **History of cardiac arrhythmia – n (%)^1^** |  |  |  |
| Yes | xx (xx.x%) | xx (xx.x%) | xx (xx.x%) |
| Diagnosis: |  |  |  |
| XXXX | xx (xx.x%) | xx (xx.x%) | xx (xx.x%) |
| XXXX | xx (xx.x%) | xx (xx.x%) | xx (xx.x%) |
| … | … | … | … |
| No | xx (xx.x%) | xx (xx.x%) | xx (xx.x%) |
| *Missing from eCRF – n (%)^2^* | *xx (xx.x%)* | *xx (xx.x%)* | *xx (xx.x%)* |
|  |  |  |  |
|  |  |  |  |
| **History of renal impairment – n (%)^1^** |  |  |  |
| Yes | xx (xx.x%) | xx (xx.x%) | xx (xx.x%) |
| Recent creatinine results available: |  |  |  |
| Yes | xx (xx.x%) | xx (xx.x%) | xx (xx.x%) |
| Creatinine result: |  |  |  |
| XXXX | xx (xx.x%) | xx (xx.x%) | xx (xx.x%) |
| XXXX | xx (xx.x%) | xx (xx.x%) | xx (xx.x%) |
| … | … | … | … |
| No | xx (xx.x%) | xx (xx.x%) | xx (xx.x%) |
| No | xx (xx.x%) | xx (xx.x%) | xx (xx.x%) |
| *Missing from eCRF – n (%)^2^* | *xx (xx.x%)* | *xx (xx.x%)* | *xx (xx.x%)* |
|  |  |  |  |
|  |  |  |  |
| **Family history of coronary artery disease – n (%)^1^** |  |  |  |
| Yes | xx (xx.x%) | xx (xx.x%) | xx (xx.x%) |
| No | xx (xx.x%) | xx (xx.x%) | xx (xx.x%) |
| Unknown | xx (xx.x%) | xx (xx.x%) | xx (xx.x%) |
| *Missing from eCRF – n (%)^2^* | *xx (xx.x%)* | *xx (xx.x%)* | *xx (xx.x%)* |
|  |  |  |  |
|  |  |  |  |
| **Previous Myocardial Infarction (ST-elevation Myocardial Infarction (STEMI) or Non-ST-elevation Myocardial Infarction (NSTEMI)) – n (%)^1^** |  |  |  |
| Yes | xx (xx.x%) | xx (xx.x%) | xx (xx.x%) |
| No | xx (xx.x%) | xx (xx.x%) | xx (xx.x%) |
| *Missing from eCRF – n (%)^2^* | *xx (xx.x%)* | *xx (xx.x%)* | *xx (xx.x%)* |
|  |  |  |  |
|  |  |  |  |
| **Tobacco use – n (%)^1^** |  |  |  |
| Former smoker (quit >90 days prior to randomisation) | xx (xx.x%) | xx (xx.x%) | xx (xx.x%) |
| Current smoker (smoking or quit <90 days prior to randomisation) | xx (xx.x%) | xx (xx.x%) | xx (xx.x%) |
| Never smoked | xx (xx.x%) | xx (xx.x%) | xx (xx.x%) |
| *Missing from eCRF – n (%)^2^* | *xx (xx.x%)* | *xx (xx.x%)* | *xx (xx.x%)* |
|  |  |  |  |
|  |  |  |  |
| **Medication use – n (%)^1^** |  |  |  |
| Aspirin | xx (xx.x%) | xx (xx.x%) | xx (xx.x%) |
| Statin | xx (xx.x%) | xx (xx.x%) | xx (xx.x%) |
| Other cholesterol lowering drug | xx (xx.x%) | xx (xx.x%) | xx (xx.x%) |
| Other cholesterol lowering drug name: |  |  |  |
| XXXX | xx (xx.x%) | xx (xx.x%) | xx (xx.x%) |
| XXXX | xx (xx.x%) | xx (xx.x%) | xx (xx.x%) |
| … | … | … | … |
| Clopidogrel | xx (xx.x%) | xx (xx.x%) | xx (xx.x%) |
| Prasugrel | xx (xx.x%) | xx (xx.x%) | xx (xx.x%) |
| Ticagrelor | xx (xx.x%) | xx (xx.x%) | xx (xx.x%) |
| Beta blocker | xx (xx.x%) | xx (xx.x%) | xx (xx.x%) |
| Calcium channel blocker | xx (xx.x%) | xx (xx.x%) | xx (xx.x%) |
| ACE inhibitor | xx (xx.x%) | xx (xx.x%) | xx (xx.x%) |
| Angiotensin receptor blocker | xx (xx.x%) | xx (xx.x%) | xx (xx.x%) |
| Alpha blocker | xx (xx.x%) | xx (xx.x%) | xx (xx.x%) |
| Diuretic | xx (xx.x%) | xx (xx.x%) | xx (xx.x%) |
| Oral nitrate | xx (xx.x%) | xx (xx.x%) | xx (xx.x%) |
| GTN spray | xx (xx.x%) | xx (xx.x%) | xx (xx.x%) |
| Any other cardiac medication | xx (xx.x%) | xx (xx.x%) | xx (xx.x%) |
| Any other cardiac medication name: |  |  |  |
| XXXX | xx (xx.x%) | xx (xx.x%) | xx (xx.x%) |
| XXXX | xx (xx.x%) | xx (xx.x%) | xx (xx.x%) |
| … | … | … | … |
| *Missing from eCRF – n (%)^2^* | *xx (xx.x%)* | *xx (xx.x%)* | *xx (xx.x%)* |
|  |  |  |  |

^1^ Denominator is the number of patients with non-missing information available.

^2^ Denominator is the number of patients

Table 6 – EQ-5D-5L questionnaire

| **Characteristic** | **Reference Group**  **(n=XXX)** | **Test Group**  **(n=XXX)** | **Total**  **(n=XXX)** |
| --- | --- | --- | --- |
|  |  |  |  |
| **Was questionnaire completed? – n (%)^1^** |  |  |  |
| Yes | xx (xx.x%) | xx (xx.x%) | xx (xx.x%) |
| Mean | xx.x | xx.x | xx.x |
| Standard deviation | xx.xx | xx.xx | xx.xx |
| Median | xx | xx | xx |
| IQR | xx to xx | xx to xx | xx to xx |
| Range | xx to xx | xx to xx | xx to xx |
| No | xx (xx.x%) | xx (xx.x%) | xx (xx.x%) |
| *Missing from eCRF – n (%)^2^* | *xx (xx.x%)* | *xx (xx.x%)* | *xx (xx.x%)* |
|  |  |  |  |
| **Mobility – Walking about – n (%)^1^** |  |  |  |
| No problems | xx (xx.x%) | xx (xx.x%) | xx (xx.x%) |
| Slight problems | xx (xx.x%) | xx (xx.x%) | xx (xx.x%) |
| Moderate problems | xx (xx.x%) | xx (xx.x%) | xx (xx.x%) |
| Severe problems | xx (xx.x%) | xx (xx.x%) | xx (xx.x%) |
| Unable to walk about | xx (xx.x%) | xx (xx.x%) | xx (xx.x%) |
| *Missing from eCRF – n (%)^2^* | *xx (xx.x%)* | *xx (xx.x%)* | *xx (xx.x%)* |
|  |  |  |  |
| **Selfcare – washing or dressing oneself – n (%)^1^** |  |  |  |
| No problems | xx (xx.x%) | xx (xx.x%) | xx (xx.x%) |
| Slight problems | xx (xx.x%) | xx (xx.x%) | xx (xx.x%) |
| Moderate problems | xx (xx.x%) | xx (xx.x%) | xx (xx.x%) |
| Severe problems | xx (xx.x%) | xx (xx.x%) | xx (xx.x%) |
| Unable to wash or dress oneself | xx (xx.x%) | xx (xx.x%) | xx (xx.x%) |
| *Missing from eCRF – n (%)^2^* | *xx (xx.x%)* | *xx (xx.x%)* | *xx (xx.x%)* |
|  |  |  |  |
| **Usual activities – work, study, housework, family or leisure activities – n (%)^1^** |  |  |  |
| No problems | xx (xx.x%) | xx (xx.x%) | xx (xx.x%) |
| Slight problems | xx (xx.x%) | xx (xx.x%) | xx (xx.x%) |
| Moderate problems | xx (xx.x%) | xx (xx.x%) | xx (xx.x%) |
| Severe problems | xx (xx.x%) | xx (xx.x%) | xx (xx.x%) |
| Unable to do one’s usual activities | xx (xx.x%) | xx (xx.x%) | xx (xx.x%) |
| *Missing from eCRF – n (%)^2^* | *xx (xx.x%)* | *xx (xx.x%)* | *xx (xx.x%)* |
|  |  |  |  |
| **Pain/Discomfort – n (%)^1^** |  |  |  |
| No pain or discomfort | xx (xx.x%) | xx (xx.x%) | xx (xx.x%) |
| Slight pain or discomfort | xx (xx.x%) | xx (xx.x%) | xx (xx.x%) |
| Moderate pain or discomfort | xx (xx.x%) | xx (xx.x%) | xx (xx.x%) |
| Severe pain or discomfort | xx (xx.x%) | xx (xx.x%) | xx (xx.x%) |
| Extreme pain or discomfort | xx (xx.x%) | xx (xx.x%) | xx (xx.x%) |
| *Missing from eCRF – n (%)^2^* | *xx (xx.x%)* | *xx (xx.x%)* | *xx (xx.x%)* |
|  |  |  |  |
| **Anxiety/Depression – n (%)^1^** |  |  |  |
| No anxiety or depression | xx (xx.x%) | xx (xx.x%) | xx (xx.x%) |
| Slight anxiety or depression | xx (xx.x%) | xx (xx.x%) | xx (xx.x%) |
| Moderate anxiety or depression | xx (xx.x%) | xx (xx.x%) | xx (xx.x%) |
| Severe anxiety or depression | xx (xx.x%) | xx (xx.x%) | xx (xx.x%) |
| Extreme anxiety or depression | xx (xx.x%) | xx (xx.x%) | xx (xx.x%) |
| *Missing from eCRF – n (%)^3^* | *xx (xx.x%)* | *xx (xx.x%)* | *xx (xx.x%)* |
|  |  |  |  |

^1^ Denominator is the number of patients with non-missing information available.

^2^ Denominator is the number of patients.

Table 7 – Health today questionnaire^1^

| **Characteristic** | **Reference Group**  **(n=XXX)** | **Test Group**  **(n=XXX)** | **Total**  **(n=XXX)** |
| --- | --- | --- | --- |
|  |  |  |  |
| **Health today – %^2^** |  |  |  |
| Mean | xx.x | xx.x | xx.x |
| Standard deviation | xx.xx | xx.xx | xx.xx |
| Median | xx | xx | xx |
| IQR | xx to xx | xx to xx | xx to xx |
| Range | xx to xx | xx to xx | xx to xx |
| *Missing from eCRF – n (%)^2^* | *xx (xx.x%)* | *xx (xx.x%)* | *xx (xx.x%)* |
|  |  |  |  |

^1^ 0 reflects to the worst health you can imagine and 100 reflects the best health you can imagine at baseline visit

^2^ Denominator is the number of patients with non-missing information available.

^3^ Denominator is the number of patients.

Table 8 – Seattle Angina – 7

| **Characteristic** | **Reference Group**  **(n=XXX)** | **Test Group**  **(n=XXX)** | **Total**  **(n=XXX)** |
| --- | --- | --- | --- |
|  |  |  |  |
| **Was questionnaire Seatle Angina – 7 completed? – n (%)^1^** |  |  |  |
| Yes | xx (xx.x%) | xx (xx.x%) | xx (xx.x%) |
| No | xx (xx.x%) | xx (xx.x%) | xx (xx.x%) |
| *Missing from eCRF – n (%)^2^* | *xx (xx.x%)* | *xx (xx.x%)* | *xx (xx.x%)* |
|  |  |  |  |
| **Activity a. Walking indoors on level ground – n (%)^1^** |  |  |  |
| Extremely limited | xx (xx.x%) | xx (xx.x%) | xx (xx.x%) |
| Quite a bit limited | xx (xx.x%) | xx (xx.x%) | xx (xx.x%) |
| Moderately limited | xx (xx.x%) | xx (xx.x%) | xx (xx.x%) |
| Slightly limited | xx (xx.x%) | xx (xx.x%) | xx (xx.x%) |
| Not limited | xx (xx.x%) | xx (xx.x%) | xx (xx.x%) |
| Limited for other reasons or did not do the activity | xx (xx.x%) | xx (xx.x%) | xx (xx.x%) |
| *Missing from eCRF – n (%)^2^* | *xx (xx.x%)* | *xx (xx.x%)* | *xx (xx.x%)* |
|  |  |  |  |
| **Activity b. Gardening, vacuuming or carrying groceries – n (%)^1^** |  |  |  |
| Extremely limited | xx (xx.x%) | xx (xx.x%) | xx (xx.x%) |
| Quite a bit limited | xx (xx.x%) | xx (xx.x%) | xx (xx.x%) |
| Moderately limited | xx (xx.x%) | xx (xx.x%) | xx (xx.x%) |
| Slightly limited | xx (xx.x%) | xx (xx.x%) | xx (xx.x%) |
| Not limited | xx (xx.x%) | xx (xx.x%) | xx (xx.x%) |
| Limited for other reasons or did not do the activity | xx (xx.x%) | xx (xx.x%) | xx (xx.x%) |
| *Missing from eCRF – n (%)^2^* | *xx (xx.x%)* | *xx (xx.x%)* | *xx (xx.x%)* |
|  |  |  |  |
| **Activity c. Lifting or moving heavy objects (e.g. furniture, children) – n (%)^1^** |  |  |  |
| Extremely limited | xx (xx.x%) | xx (xx.x%) | xx (xx.x%) |
| Quite a bit limited | xx (xx.x%) | xx (xx.x%) | xx (xx.x%) |
| Moderately limited | xx (xx.x%) | xx (xx.x%) | xx (xx.x%) |
| Slightly limited | xx (xx.x%) | xx (xx.x%) | xx (xx.x%) |
| Not limited | xx (xx.x%) | xx (xx.x%) | xx (xx.x%) |
| Limited for other reasons or did not do the activity | xx (xx.x%) | xx (xx.x%) | xx (xx.x%) |
| *Missing from eCRF – n (%)^2^* | *xx (xx.x%)* | *xx (xx.x%)* | *xx (xx.x%)* |
|  |  |  |  |
| **Over the past 4 weeks, on average, how many times have you had chest pain, chest tightness or angina? – n (%)^1^** |  |  |  |
| 4 or more times per day | xx (xx.x%) | xx (xx.x%) | xx (xx.x%) |
| 1 – 3 times per day | xx (xx.x%) | xx (xx.x%) | xx (xx.x%) |
| 3 or more times per week (not every day) | xx (xx.x%) | xx (xx.x%) | xx (xx.x%) |
| 1 – 2 times per week | xx (xx.x%) | xx (xx.x%) | xx (xx.x%) |
| Less than once per week | xx (xx.x%) | xx (xx.x%) | xx (xx.x%) |
| None over past 4 weeks | xx (xx.x%) | xx (xx.x%) | xx (xx.x%) |
| *Missing from eCRF – n (%)^2^* | *xx (xx.x%)* | *xx (xx.x%)* | *xx (xx.x%)* |
|  |  |  |  |
| **Over the past 4 weeks, on average, how many times have you had to take nitroglycerin (tablets or spray) for your chest pain, chest tightness or angina? – n (%)^1^** |  |  |  |
| 4 or more times per day | xx (xx.x%) | xx (xx.x%) | xx (xx.x%) |
| 1 – 3 times per day | xx (xx.x%) | xx (xx.x%) | xx (xx.x%) |
| 3 or more times per week (not every day) | xx (xx.x%) | xx (xx.x%) | xx (xx.x%) |
| 1 – 2 times per week | xx (xx.x%) | xx (xx.x%) | xx (xx.x%) |
| Less than once per week | xx (xx.x%) | xx (xx.x%) | xx (xx.x%) |
| None over past 4 weeks | xx (xx.x%) | xx (xx.x%) | xx (xx.x%) |
| *Missing from eCRF – n (%)^2^* | *xx (xx.x%)* | *xx (xx.x%)* | *xx (xx.x%)* |
|  |  |  |  |
| **Over the past 4 weeks, how much has your chest pain, chest tightness or angina limited your enjoyment of life? – n (%)^1^** |  |  |  |
| Extremely limited | xx (xx.x%) | xx (xx.x%) | xx (xx.x%) |
| Quite a bit limited | xx (xx.x%) | xx (xx.x%) | xx (xx.x%) |
| Moderately limited | xx (xx.x%) | xx (xx.x%) | xx (xx.x%) |
| Slightly limited | xx (xx.x%) | xx (xx.x%) | xx (xx.x%) |
| Not at all limited | xx (xx.x%) | xx (xx.x%) | xx (xx.x%) |
| *Missing from eCRF – n (%)^2^* | *xx (xx.x%)* | *xx (xx.x%)* | *xx (xx.x%)* |
|  |  |  |  |
| **If you had to spend the rest of your life with your chest pain, chest tightness or angina the way it is now, how would you feel about this? – n (%)^1^** |  |  |  |
| Not satisfied at all | xx (xx.x%) | xx (xx.x%) | xx (xx.x%) |
| Mostly dissatisfied | xx (xx.x%) | xx (xx.x%) | xx (xx.x%) |
| Somewhat satisfied | xx (xx.x%) | xx (xx.x%) | xx (xx.x%) |
| Mostly satisfied | xx (xx.x%) | xx (xx.x%) | xx (xx.x%) |
| Completely satisfied | xx (xx.x%) | xx (xx.x%) | xx (xx.x%) |
| *Missing from eCRF – n (%)^2^* | *xx (xx.x%)* | *xx (xx.x%)* | *xx (xx.x%)* |
|  |  |  |  |

^1^ Denominator is the number of patients with non-missing information available.

^2^ Denominator is the number of patients.

Table 9 – Diamond Forrester Risk Score

| **Characteristic** | **Reference Group**  **(n=XXX)** | **Test Group**  **(n=XXX)** | **Total**  **(n=XXX)** |
| --- | --- | --- | --- |
|  |  |  |  |
| **Diamond Forrester Risk Score available – n (%)^1^** |  |  |  |
| Yes | xx (xx.x%) | xx (xx.x%) | xx (xx.x%) |
| Result - %: |  |  |  |
| Mean | xx.x | xx.x | xx.x |
| Standard deviation | xx.xx | xx.xx | xx.xx |
| Median | xx | xx | xx |
| IQR | xx to xx | xx to xx | xx to xx |
| Range | xx to xx | xx to xx | xx to xx |
| *Missing from eCRF – n (%)^2^* | *xx (xx.x%)* | *xx (xx.x%)* | *xx (xx.x%)* |
| No | xx (xx.x%) | xx (xx.x%) | xx (xx.x%) |
| Alternative risk score available: |  |  |  |
| Yes - name: | xx (xx.x%) | xx (xx.x%) | xx (xx.x%) |
| XXXX | xx (xx.x%) | xx (xx.x%) | xx (xx.x%) |
| XXXX | xx (xx.x%) | xx (xx.x%) | xx (xx.x%) |
| … | … | … | … |
| No | xx (xx.x%) | xx (xx.x%) | xx (xx.x%) |
| *Missing from eCRF – n (%)^2^* | *xx (xx.x%)* | *xx (xx.x%)* | *xx (xx.x%)* |
|  |  |  |  |

^1^ Denominator is the number of patients with non-missing information available.

^2^ Denominator is the number of patients.

Table 10 – First Choice Test

| **Characteristic** | **Reference Group**  **(n=XXX)** | **Test Group**  **(n=XXX)** | **Total**  **(n=XXX)** |
| --- | --- | --- | --- |
|  |  |  |  |
| **First choice test – n (%)^1^** |  |  |  |
| Invasive coronary angiogram | xx (xx.x%) | xx (xx.x%) | xx (xx.x%) |
| Stress echo | xx (xx.x%) | xx (xx.x%) | xx (xx.x%) |
| Nuclear Medicine Perfusion Scan | xx (xx.x%) | xx (xx.x%) | xx (xx.x%) |
| Stress Magnetic Resonance Imaging | xx (xx.x%) | xx (xx.x%) | xx (xx.x%) |
| CT calcium score | xx (xx.x%) | xx (xx.x%) | xx (xx.x%) |
| CT Coronary Angiogram | xx (xx.x%) | xx (xx.x%) | xx (xx.x%) |
| Exercise Tolerance Test | xx (xx.x%) | xx (xx.x%) | xx (xx.x%) |
| Other: | xx (xx.x%) | xx (xx.x%) | xx (xx.x%) |
| XXXX | xx (xx.x%) | xx (xx.x%) | xx (xx.x%) |
| XXXX | xx (xx.x%) | xx (xx.x%) | xx (xx.x%) |
| … | … | … | … |
| *Missing from eCRF – n (%)^2^* | *xx (xx.x%)* | *xx (xx.x%)* | *xx (xx.x%)* |
|  |  |  |  |

^1^ Denominator is the number of patients with non-missing information available.

^2^ Denominator is the number of patients.

Table 11 – Routine Investigations Referral (Reference Group only)

| **Characteristic** | **Reference Group**  **(n=XXX)** |
| --- | --- |
|  |  |
| **Routine Investigations Referral – n (%)^1^** |  |
| Invasive coronary angiogram | xx (xx.x%) |
| Stress echo | xx (xx.x%) |
| Nuclear Medicine Perfusion Scan | xx (xx.x%) |
| Stress Magnetic Resonance Imaging | xx (xx.x%) |
| CT calcium score | xx (xx.x%) |
| CT Coronary Angiogram | xx (xx.x%) |
| Exercise Tolerance Test | xx (xx.x%) |
| Other: | xx (xx.x%) |
| XXXX | xx (xx.x%) |
| XXXX | xx (xx.x%) |
| … | … |
| *Missing from eCRF – n (%)^2^* | *xx (xx.x%)* |
|  |  |
|  |  |
| First Choice test differs to investigation referred for: |  |
| Yes | xx (xx.x%) |
| No | xx (xx.x%) |
| *Missing from eCRF – n (%)^2^* | *xx (xx.x%)* |
|  |  |

^1^ Denominator is the number of patients with non-missing information available.

^2^ Denominator is the number of patients.

## Treatment information

Table 12 – CTCA Data (Test group only)

| **Characteristic** | **Test Group**  **(n=XXX)** |
| --- | --- |
|  |  |
| **Did patient undergo CTCA? – n (%)^1^** |  |
| Yes | xx (xx.x%) |
| No | xx (xx.x%) |
| *Missing from eCRF – n (%)^2^* | *xx (xx.x%)* |
|  |  |
|  |  |
| **Heart rate prior to CTCA – beats/minute^1^** |  |
| Mean | xx.x |
| Standard deviation | xx.xx |
| Median | xx |
| IQR | xx to xx |
| Range | xx to xx |
| *Missing from eCRF – n (%)^2^* | *xx (xx.x%)* |
|  |  |
| **Systolic Blood pressure prior to CTCA – mmHg^1^** |  |
| Mean | xx.x |
| Standard deviation | xx.xx |
| Median | xx |
| IQR | xx to xx |
| Range | xx to xx |
| *Missing from eCRF – n (%)^2^* | *xx (xx.x%)* |
|  |  |
| **Diastolic Blood pressure prior to CTCA – mmHg^1^** |  |
| Mean | xx.x |
| Standard deviation | xx.xx |
| Median | xx |
| IQR | xx to xx |
| Range | xx to xx |
| *Missing from eCRF – n (%)^2^* | *xx (xx.x%)* |
|  |  |
|  |  |
| **Average heart rate during CTCA – beats/minute^1^** |  |
| Mean | xx.x |
| Standard deviation | xx.xx |
| Median | xx |
| IQR | xx to xx |
| Range | xx to xx |
| *Missing from eCRF – n (%)^2^* | *xx (xx.x%)* |
|  |  |
| **Average systolic blood pressure during CTCA – mmHg^1^** |  |
| Mean | xx.x |
| Standard deviation | xx.xx |
| Median | xx |
| IQR | xx to xx |
| Range | xx to xx |
| *Missing from eCRF – n (%)^2^* | *xx (xx.x%)* |
|  |  |
| **Average diastolic blood pressure during CTCA – mmHg^1^** |  |
| Mean | xx.x |
| Standard deviation | xx.xx |
| Median | xx |
| IQR | xx to xx |
| Range | xx to xx |
| *Missing from eCRF – n (%)^2^* | *xx (xx.x%)* |
|  |  |
|  |  |
| **Beta-blockers administered prior to CTCA – n(%)^1^** |  |
| Yes | xx (xx.x%) |
| No | xx (xx.x%) |
| Other bradycardic agent: |  |
| Yes | xx (xx.x%) |
| No | xx (xx.x%) |
| *Missing from eCRF – n (%)^2^* | *xx (xx.x%)* |
|  |  |
|  |  |
| **Nitrates administered prior to CTCA – n(%)^1^** |  |
| Yes | xx (xx.x%) |
| No | xx (xx.x%) |
| *Missing from eCRF – n (%)^2^* | *xx (xx.x%)* |
|  |  |
|  |  |
| **Scan mode – n(%)^1^** |  |
| Prospective | xx (xx.x%) |
| Retrospective | xx (xx.x%) |
| Flash | xx (xx.x%) |
| Unknown | xx (xx.x%) |
| *Missing from eCRF – n (%)^2^* | *xx (xx.x%)* |
|  |  |
|  |  |
| **Padding used – n(%)^1^** |  |
| Yes | xx (xx.x%) |
| No | xx (xx.x%) |
| *Missing from eCRF – n (%)^2^* | *xx (xx.x%)* |
|  |  |
|  |  |
| **Best phase – n(%)^1^** |  |
| Systolic | xx (xx.x%) |
| Diastolic | xx (xx.x%) |
| *Missing from eCRF – n (%)^2^* | *xx (xx.x%)* |
|  |  |
|  |  |
| **Contrast volume – mls^1^** |  |
| Mean | xx.x |
| Standard deviation | xx.xx |
| Median | xx |
| IQR | xx to xx |
| Range | xx to xx |
| *Missing from eCRF – n (%)^2^* | *xx (xx.x%)* |
|  |  |
|  |  |
| **Radiation exposure – DLP in mGy.cm^2 1^** |  |
| Mean | xx.x |
| Standard deviation | xx.xx |
| Median | xx |
| IQR | xx to xx |
| Range | xx to xx |
| *Missing from eCRF – n (%)^2^* | *xx (xx.x%)* |
|  |  |

^1^ Denominator is the number of patients with non-missing information available.

^2^ Denominator is the number of patients.

Table 13 – CTCA Results (Test group only)

| **Characteristic** | **Test Group**  **(n=XXX)** |
| --- | --- |
|  |  |
| **Did patient undergo CTCA? – n (%)^1^** |  |
| Yes | xx (xx.x%) |
| No | xx (xx.x%) |
| *Missing from eCRF – n (%)^2^* | *xx (xx.x%)* |
|  |  |
|  |  |
| **Calcium score performed – n (%)^1^** |  |
| Yes | xx (xx.x%) |
| Agatston Units: |  |
| Mean | xx.x |
| Standard deviation | xx.xx |
| Median | xx |
| IQR | xx to xx |
| Range | xx to xx |
| *Missing from eCRF – n (%)^2^* | *xx (xx.x%)* |
| No | xx (xx.x%) |
|  |  |
|  |  |
| CORONARY ANATOMY |  |
|  |  |
| **RCA - proximal – n (%)^1^** |  |
| 0% | xx (xx.x%) |
| >0% & <40% | xx (xx.x%) |
| ≥40% & ≤50% | xx (xx.x%) |
| >50% & ≤70% | xx (xx.x%) |
| >70% & ≤90% | xx (xx.x%) |
| >90% | xx (xx.x%) |
| Occluded | xx (xx.x%) |
| Vessel too small to evaluate | xx (xx.x%) |
| Vessel absent | xx (xx.x%) |
| Non-evaluable (image quality) | xx (xx.x%) |
| *Missing from eCRF – n (%)^2^* | *xx (xx.x%)* |
|  |  |
| **RCA - mid – n (%)^1^** |  |
| 0% | xx (xx.x%) |
| >0% & <40% | xx (xx.x%) |
| ≥40% & ≤50% | xx (xx.x%) |
| >50% & ≤70% | xx (xx.x%) |
| >70% & ≤90% | xx (xx.x%) |
| >90% | xx (xx.x%) |
| Occluded | xx (xx.x%) |
| Vessel too small to evaluate | xx (xx.x%) |
| Vessel absent | xx (xx.x%) |
| Non-evaluable (image quality) | xx (xx.x%) |
| *Missing from eCRF – n (%)^2^* | *xx (xx.x%)* |
|  |  |
| **RCA - distal – n (%)^1^** |  |
| 0% | xx (xx.x%) |
| >0% & <40% | xx (xx.x%) |
| ≥40% & ≤50% | xx (xx.x%) |
| >50% & ≤70% | xx (xx.x%) |
| >70% & ≤90% | xx (xx.x%) |
| >90% | xx (xx.x%) |
| Occluded | xx (xx.x%) |
| Vessel too small to evaluate | xx (xx.x%) |
| Vessel absent | xx (xx.x%) |
| Non-evaluable (image quality) | xx (xx.x%) |
| *Missing from eCRF – n (%)^2^* | *xx (xx.x%)* |
|  |  |
| **RCA – posterior descending artery – n (%)^1^** |  |
| 0% | xx (xx.x%) |
| >0% & <40% | xx (xx.x%) |
| ≥40% & ≤50% | xx (xx.x%) |
| >50% & ≤70% | xx (xx.x%) |
| >70% & ≤90% | xx (xx.x%) |
| >90% | xx (xx.x%) |
| Occluded | xx (xx.x%) |
| Vessel too small to evaluate | xx (xx.x%) |
| Vessel absent | xx (xx.x%) |
| Non-evaluable (image quality) | xx (xx.x%) |
| *Missing from eCRF – n (%)^2^* | *xx (xx.x%)* |
|  |  |
| **Left main stem – n (%)^1^** |  |
| 0% | xx (xx.x%) |
| >0% & <40% | xx (xx.x%) |
| ≥40% & ≤50% | xx (xx.x%) |
| >50% & ≤70% | xx (xx.x%) |
| >70% & ≤90% | xx (xx.x%) |
| >90% | xx (xx.x%) |
| Occluded | xx (xx.x%) |
| Vessel too small to evaluate | xx (xx.x%) |
| Vessel absent | xx (xx.x%) |
| Non-evaluable (image quality) | xx (xx.x%) |
| *Missing from eCRF – n (%)^2^* | *xx (xx.x%)* |
|  |  |
| **LAD - proximal – n (%)^1^** |  |
| 0% | xx (xx.x%) |
| >0% & <40% | xx (xx.x%) |
| ≥40% & ≤50% | xx (xx.x%) |
| >50% & ≤70% | xx (xx.x%) |
| >70% & ≤90% | xx (xx.x%) |
| >90% | xx (xx.x%) |
| Occluded | xx (xx.x%) |
| Vessel too small to evaluate | xx (xx.x%) |
| Vessel absent | xx (xx.x%) |
| Non-evaluable (image quality) | xx (xx.x%) |
| *Missing from eCRF – n (%)^2^* | *xx (xx.x%)* |
|  |  |
| **LAD - mid – n (%)^1^** |  |
| 0% | xx (xx.x%) |
| >0% & <40% | xx (xx.x%) |
| ≥40% & ≤50% | xx (xx.x%) |
| >50% & ≤70% | xx (xx.x%) |
| >70% & ≤90% | xx (xx.x%) |
| >90% | xx (xx.x%) |
| Occluded | xx (xx.x%) |
| Vessel too small to evaluate | xx (xx.x%) |
| Vessel absent | xx (xx.x%) |
| Non-evaluable (image quality) | xx (xx.x%) |
| *Missing from eCRF – n (%)^2^* | *xx (xx.x%)* |
|  |  |
| **LAD - distal – n (%)^1^** |  |
| 0% | xx (xx.x%) |
| >0% & <40% | xx (xx.x%) |
| ≥40% & ≤50% | xx (xx.x%) |
| >50% & ≤70% | xx (xx.x%) |
| >70% & ≤90% | xx (xx.x%) |
| >90% | xx (xx.x%) |
| Occluded | xx (xx.x%) |
| Vessel too small to evaluate | xx (xx.x%) |
| Vessel absent | xx (xx.x%) |
| Non-evaluable (image quality) | xx (xx.x%) |
| *Missing from eCRF – n (%)^2^* | *xx (xx.x%)* |
|  |  |
| **1^st^ diagonal – n (%)^1^** |  |
| 0% | xx (xx.x%) |
| >0% & <40% | xx (xx.x%) |
| ≥40% & ≤50% | xx (xx.x%) |
| >50% & ≤70% | xx (xx.x%) |
| >70% & ≤90% | xx (xx.x%) |
| >90% | xx (xx.x%) |
| Occluded | xx (xx.x%) |
| Vessel too small to evaluate | xx (xx.x%) |
| Vessel absent | xx (xx.x%) |
| Non-evaluable (image quality) | xx (xx.x%) |
| *Missing from eCRF – n (%)^2^* | *xx (xx.x%)* |
|  |  |
| **Additional 1^st^ diagonal – n (%)^1^** |  |
| 0% | xx (xx.x%) |
| >0% & <40% | xx (xx.x%) |
| ≥40% & ≤50% | xx (xx.x%) |
| >50% & ≤70% | xx (xx.x%) |
| >70% & ≤90% | xx (xx.x%) |
| >90% | xx (xx.x%) |
| Occluded | xx (xx.x%) |
| Vessel too small to evaluate | xx (xx.x%) |
| Vessel absent | xx (xx.x%) |
| Non-evaluable (image quality) | xx (xx.x%) |
| *Missing from eCRF – n (%)^2^* | *xx (xx.x%)* |
|  |  |
| **2^nd^ diagonal – n (%)^1^** |  |
| 0% | xx (xx.x%) |
| >0% & <40% | xx (xx.x%) |
| ≥40% & ≤50% | xx (xx.x%) |
| >50% & ≤70% | xx (xx.x%) |
| >70% & ≤90% | xx (xx.x%) |
| >90% | xx (xx.x%) |
| Occluded | xx (xx.x%) |
| Vessel too small to evaluate | xx (xx.x%) |
| Vessel absent | xx (xx.x%) |
| Non-evaluable (image quality) | xx (xx.x%) |
| *Missing from eCRF – n (%)^2^* | *xx (xx.x%)* |
|  |  |
| **Additional 2^nd^ diagonal – n (%)^1^** |  |
| 0% | xx (xx.x%) |
| >0% & <40% | xx (xx.x%) |
| ≥40% & ≤50% | xx (xx.x%) |
| >50% & ≤70% | xx (xx.x%) |
| >70% & ≤90% | xx (xx.x%) |
| >90% | xx (xx.x%) |
| Occluded | xx (xx.x%) |
| Vessel too small to evaluate | xx (xx.x%) |
| Vessel absent | xx (xx.x%) |
| Non-evaluable (image quality) | xx (xx.x%) |
| *Missing from eCRF – n (%)^2^* | *xx (xx.x%)* |
|  |  |
| **Prox circumflex – n (%)^1^** |  |
| 0% | xx (xx.x%) |
| >0% & <40% | xx (xx.x%) |
| ≥40% & ≤50% | xx (xx.x%) |
| >50% & ≤70% | xx (xx.x%) |
| >70% & ≤90% | xx (xx.x%) |
| >90% | xx (xx.x%) |
| Occluded | xx (xx.x%) |
| Vessel too small to evaluate | xx (xx.x%) |
| Vessel absent | xx (xx.x%) |
| Non-evaluable (image quality) | xx (xx.x%) |
| *Missing from eCRF – n (%)^2^* | *xx (xx.x%)* |
|  |  |
| **Intermediate or Ramus Intermedius – n (%)^1^** |  |
| 0% | xx (xx.x%) |
| >0% & <40% | xx (xx.x%) |
| ≥40% & ≤50% | xx (xx.x%) |
| >50% & ≤70% | xx (xx.x%) |
| >70% & ≤90% | xx (xx.x%) |
| >90% | xx (xx.x%) |
| Occluded | xx (xx.x%) |
| Vessel too small to evaluate | xx (xx.x%) |
| Vessel absent | xx (xx.x%) |
| Non-evaluable (image quality) | xx (xx.x%) |
| *Missing from eCRF – n (%)^2^* | *xx (xx.x%)* |
|  |  |
| **Obtuse marginal – n (%)^1^** |  |
| 0% | xx (xx.x%) |
| >0% & <40% | xx (xx.x%) |
| ≥40% & ≤50% | xx (xx.x%) |
| >50% & ≤70% | xx (xx.x%) |
| >70% & ≤90% | xx (xx.x%) |
| >90% | xx (xx.x%) |
| Occluded | xx (xx.x%) |
| Vessel too small to evaluate | xx (xx.x%) |
| Vessel absent | xx (xx.x%) |
| Non-evaluable (image quality) | xx (xx.x%) |
| *Missing from eCRF – n (%)^2^* | *xx (xx.x%)* |
|  |  |
| **Obtuse marginal (b) – n (%)^1^** |  |
| 0% | xx (xx.x%) |
| >0% & <40% | xx (xx.x%) |
| ≥40% & ≤50% | xx (xx.x%) |
| >50% & ≤70% | xx (xx.x%) |
| >70% & ≤90% | xx (xx.x%) |
| >90% | xx (xx.x%) |
| Occluded | xx (xx.x%) |
| Vessel too small to evaluate | xx (xx.x%) |
| Vessel absent | xx (xx.x%) |
| Non-evaluable (image quality) | xx (xx.x%) |
| *Missing from eCRF – n (%)^2^* | *xx (xx.x%)* |
|  |  |
| **Distal circumflex artery – n (%)^1^** |  |
| 0% | xx (xx.x%) |
| >0% & <40% | xx (xx.x%) |
| ≥40% & ≤50% | xx (xx.x%) |
| >50% & ≤70% | xx (xx.x%) |
| >70% & ≤90% | xx (xx.x%) |
| >90% | xx (xx.x%) |
| Occluded | xx (xx.x%) |
| Vessel too small to evaluate | xx (xx.x%) |
| Vessel absent | xx (xx.x%) |
| Non-evaluable (image quality) | xx (xx.x%) |
| *Missing from eCRF – n (%)^2^* | *xx (xx.x%)* |
|  |  |
| **Left posterolateral – n (%)^1^** |  |
| 0% | xx (xx.x%) |
| >0% & <40% | xx (xx.x%) |
| ≥40% & ≤50% | xx (xx.x%) |
| >50% & ≤70% | xx (xx.x%) |
| >70% & ≤90% | xx (xx.x%) |
| >90% | xx (xx.x%) |
| Occluded | xx (xx.x%) |
| Vessel too small to evaluate | xx (xx.x%) |
| Vessel absent | xx (xx.x%) |
| Non-evaluable (image quality) | xx (xx.x%) |
| *Missing from eCRF – n (%)^2^* | *xx (xx.x%)* |
|  |  |
| **Posterior descending – n (%)^1^** |  |
| 0% | xx (xx.x%) |
| >0% & <40% | xx (xx.x%) |
| ≥40% & ≤50% | xx (xx.x%) |
| >50% & ≤70% | xx (xx.x%) |
| >70% & ≤90% | xx (xx.x%) |
| >90% | xx (xx.x%) |
| Occluded | xx (xx.x%) |
| Vessel too small to evaluate | xx (xx.x%) |
| Vessel absent | xx (xx.x%) |
| Non-evaluable (image quality) | xx (xx.x%) |
| *Missing from eCRF – n (%)^2^* | *xx (xx.x%)* |
|  |  |
| **Posterolateral branch from RCA – n (%)^1^** |  |
| 0% | xx (xx.x%) |
| >0% & <40% | xx (xx.x%) |
| ≥40% & ≤50% | xx (xx.x%) |
| >50% & ≤70% | xx (xx.x%) |
| >70% & ≤90% | xx (xx.x%) |
| >90% | xx (xx.x%) |
| Occluded | xx (xx.x%) |
| Vessel too small to evaluate | xx (xx.x%) |
| Vessel absent | xx (xx.x%) |
| Non-evaluable (image quality) | xx (xx.x%) |
| *Missing from eCRF – n (%)^2^* | *xx (xx.x%)* |
|  |  |
| **1^st^ Posterolateral branch from segment 16 (a) – n (%)^1^** |  |
| 0% | xx (xx.x%) |
| >0% & <40% | xx (xx.x%) |
| ≥40% & ≤50% | xx (xx.x%) |
| >50% & ≤70% | xx (xx.x%) |
| >70% & ≤90% | xx (xx.x%) |
| >90% | xx (xx.x%) |
| Occluded | xx (xx.x%) |
| Vessel too small to evaluate | xx (xx.x%) |
| Vessel absent | xx (xx.x%) |
| Non-evaluable (image quality) | xx (xx.x%) |
| *Missing from eCRF – n (%)^2^* | *xx (xx.x%)* |
|  |  |
| **1^st^ Posterolateral branch from segment 16 (b) – n (%)^1^** |  |
| 0% | xx (xx.x%) |
| >0% & <40% | xx (xx.x%) |
| ≥40% & ≤50% | xx (xx.x%) |
| >50% & ≤70% | xx (xx.x%) |
| >70% & ≤90% | xx (xx.x%) |
| >90% | xx (xx.x%) |
| Occluded | xx (xx.x%) |
| Vessel too small to evaluate | xx (xx.x%) |
| Vessel absent | xx (xx.x%) |
| Non-evaluable (image quality) | xx (xx.x%) |
| *Missing from eCRF – n (%)^2^* | *xx (xx.x%)* |
|  |  |
| **1^st^ Posterolateral branch from segment 16 (c) – n (%)^1^** |  |
| 0% | xx (xx.x%) |
| >0% & <40% | xx (xx.x%) |
| ≥40% & ≤50% | xx (xx.x%) |
| >50% & ≤70% | xx (xx.x%) |
| >70% & ≤90% | xx (xx.x%) |
| >90% | xx (xx.x%) |
| Occluded | xx (xx.x%) |
| Vessel too small to evaluate | xx (xx.x%) |
| Vessel absent | xx (xx.x%) |
| Non-evaluable (image quality) | xx (xx.x%) |
| *Missing from eCRF – n (%)^2^* | *xx (xx.x%)* |
|  |  |
|  |  |
| **Summary results of CTCA – n (%)^1^** |  |
| Any atheroma > 40% | xx (xx.x%) |
| Unobstructed coronaries | xx (xx.x%) |
| Indeterminate | xx (xx.x%) |
| *Missing from eCRF – n (%)^2^* | *xx (xx.x%)* |
|  |  |
| **CTCA results signed by Radiologist– n (%)^1^** |  |
| Yes | xx (xx.x%) |
| No | xx (xx.x%) |
| *Missing from eCRF – n (%)^2^* | *xx (xx.x%)* |
|  |  |

^1^ Denominator is the number of patients with non-missing information available.

^2^ Denominator is the number of patients

Table 14 – FFR_CT_ Results (Test group only)

| **Characteristic** | **Test Group**  **(n=XXX)** |
| --- | --- |
|  |  |
| **Was FFR_CT_ performed? – n (%)^1^** |  |
| Yes | xx (xx.x%) |
| No | xx (xx.x%) |
| Reason: |  |
| No lesion equal or greater than 40% | xx (xx.x%) |
| HeartFlow unable to analyse | xx (xx.x%) |
| *Missing from eCRF – n (%)^2^* | *xx (xx.x%)* |
|  |  |
|  |  |
| **Ischaemia present for any stentable or graftable vessels? (i.e. FFR value < 0.8) – n (%)^1^** |  |
| Yes | xx (xx.x%) |
| No | xx (xx.x%) |
| *Missing from eCRF – n (%)^2^* | *xx (xx.x%)* |
|  |  |
| **Ischaemic vessels with FFR result – n (%)^1^** |  |
| Distal 3^rd^ of RCA | xx (xx.x%) |
| PDA right | xx (xx.x%) |
| LV branch right | xx (xx.x%) |
| Distal 3^rd^ of LAD | xx (xx.x%) |
| Distal 3^rd^ of circumflex | xx (xx.x%) |
| OM1 | xx (xx.x%) |
| OM2 | xx (xx.x%) |
| OM3 | xx (xx.x%) |
| PDA left | xx (xx.x%) |
| 1^st^ diagonal | xx (xx.x%) |
| 2^nd^ diagonal | xx (xx.x%) |
| Intermediate | xx (xx.x%) |
| Other | xx (xx.x%) |
| XXXX | xx (xx.x%) |
| XXXX | xx (xx.x%) |
| … | xx (xx.x%) |
| *Missing from eCRF – n (%)^2^* | *xx (xx.x%)* |
|  |  |
|  |  |
| **FFR_CT_ result –^1^** |  |
| Mean | xx.x |
| Standard deviation | xx.xx |
| Median | xx |
| IQR | xx to xx |
| Range | xx to xx |
| *Missing from eCRF – n (%)^2^* | *xx (xx.x%)* |
|  |  |
| **FFR_CT_ considered when making treatment plan – n (%)^1^** |  |
| Yes | xx (xx.x%) |
| No | xx (xx.x%) |
| Reason: |  |
| XXXX | xx (xx.x%) |
| XXXX | xx (xx.x%) |
| … | … |
| *Missing from eCRF – n (%)^2^* | *xx (xx.x%)* |
|  |  |
| **After FFR_CT_ results, ICA requested – n (%)^1^** |  |
| Yes | xx (xx.x%) |
| No | xx (xx.x%) |
| *Missing from eCRF – n (%)^2^* | *xx (xx.x%)* |
|  |  |
| **After FFR_CT_ results, any non-invasive tests requested – n (%)^1^** |  |
| Yes | xx (xx.x%) |
| Stress Echo | xx (xx.x%) |
| Nuclear Medicine Perfusion Scan | xx (xx.x%) |
| Stress Magnetic Resonance Imaging | xx (xx.x%) |
| Exercise Tolerance test | xx (xx.x%) |
| Other | xx (xx.x%) |
| XXXX | xx (xx.x%) |
| XXXX | xx (xx.x%) |
| … | … |
| No | xx (xx.x%) |
| *Missing from eCRF – n (%)^2^* | *xx (xx.x%)* |
|  |  |

^1^ Denominator is the number of patients with non-missing information available.

^2^ Denominator is the number of patients

Table 15 – Final Treatment Plan

| **Characteristic** | **Test Group**  **(n=XXX)** |
| --- | --- |
|  |  |
| **Treatment plan – n (%)^1^** |  |
| Optimal Medical Therapy (OMT) alone | xx (xx.x%) |
| Percutaneous Coronary Intervention (PCI) | xx (xx.x%) |
| Coronary Artery Bypass Graft (CABG) | xx (xx.x%) |
| Vessels: |  |
| RCA | xx (xx.x%) |
| PDA right | xx (xx.x%) |
| LV branch right | xx (xx.x%) |
| Left main stem | xx (xx.x%) |
| LAD | xx (xx.x%) |
| 1^st^ Diagonal | xx (xx.x%) |
| 2^nd^ Diagonal | xx (xx.x%) |
| Circumflex | xx (xx.x%) |
| OM1 | xx (xx.x%) |
| OM2 | xx (xx.x%) |
| OM3 | xx (xx.x%) |
| PDA left | xx (xx.x%) |
| Intermediate | xx (xx.x%) |
| *Missing from eCRF – n (%)^2^* | *xx (xx.x%)* |
|  |  |

^1^ Denominator is the number of patients with non-missing information available.

^2^ Denominator is the number of patients

## Primary outcome analyses

Table 16 – Resource use over nine months, by group

| **Resource** | **Reference Group**  **(n=XXX)** | **Test Group**  **(n=XXX)** | **Total**  **(n=XXX)** |
| --- | --- | --- | --- |
|  |  |  |  |
| **Non-Invasive Tests – n^1^ (n^2^)** |  |  |  |
| CTCA | xx (xx) | xx (xx) | xx (xx) |
| FFR_CT_ | xx (xx) | xx (xx) | xx (xx) |
| Stress Echo | xx (xx) | xx (xx) | xx (xx) |
| Perfusion Scan | xx (xx) | xx (xx) | xx (xx) |
| Stress MRI | xx (xx) | xx (xx) | xx (xx) |
| Exercise ECG | xx (xx) | xx (xx) | xx (xx) |
|  |  |  |  |
|  |  |  |  |
| **Invasive Procedures – n^1^ (n^2^)** |  |  |  |
| Coronary Angiogram | xx (xx) | xx (xx) | xx (xx) |
| FFR (Invasive) | xx (xx) | xx (xx) | xx (xx) |
| PCI | xx (xx) | xx (xx) | xx (xx) |
| CABG | xx (xx) | xx (xx) | xx (xx) |
|  |  |  |  |
|  |  |  |  |
| Hospitalisations – n^1^ (n^2^) |  |  |  |
| Myocardial Infarction | xx (xx) | xx (xx) | xx (xx) |
| Acute coronary syndrome | xx (xx) | xx (xx) | xx (xx) |
| Chest Pain | xx (xx) | xx (xx) | xx (xx) |
| Stroke | xx (xx) | xx (xx) | xx (xx) |
| Transient Ischemic Attack | xx (xx) | xx (xx) | xx (xx) |
| Other | xx (xx) | xx (xx) | xx (xx) |
|  |  |  |  |
|  |  |  |  |
| **Emergency Department Visits – n^1^ (n^2^)** | xx (xx) | xx (xx) | xx (xx) |
|  |  |  |  |
|  |  |  |  |
| **Cardiac Outpatient Visits – n^1^ (n^2^)** | xx (xx) | xx (xx) | xx (xx) |
|  |  |  |  |
|  |  |  |  |
| **Medications – n^1^ (n^2^)** |  |  |  |
| Statin | xx (xx) | xx (xx) | xx (xx) |
| Aspirin | xx (xx) | xx (xx) | xx (xx) |
| Antiplatelet^3^ | xx (xx) | xx (xx) | xx (xx) |
| Beta-Blocker | xx (xx) | xx (xx) | xx (xx) |
| Calcium Blocker | xx (xx) | xx (xx) | xx (xx) |
| Oral Nitrate | xx (xx) | xx (xx) | xx (xx) |
| ACE Inhibitor | xx (xx) | xx (xx) | xx (xx) |
| ARB | xx (xx) | xx (xx) | xx (xx) |
| Alpha Blocker | xx (xx) | xx (xx) | xx (xx) |
|  |  |  |  |

^1^ Number of resources used

^2^ Number of patients with any use

^3^ Clopidogrel, Ticagrelor, or Prasugrel

Table 17 – Total medical costs over nine months, by group

| **Resource** | **Reference Group**  **(n=XXX)** | **Test Group**  **(n=XXX)** | **P** |
| --- | --- | --- | --- |
|  |  |  |  |
| **All patients** |  |  |  |
| Initial testing |  |  |  |
| Mean | xx.x | xx.x | N/A |
| Median | xx | xx |  |
| *Missing from eCRF – n (%)^2^* | *xx (xx.x%)* | *xx (xx.x%)* |  |
| Follow up costs |  |  |  |
| Mean | xx.x | xx.x | x.xxx |
| Median | xx | xx |  |
| *Missing from eCRF – n (%)^2^* | *xx (xx.x%)* | *xx (xx.x%)* |  |
| Total costs |  |  |  |
| Mean | xx.x | xx.x | x.xxx |
| Median | xx | xx |  |
| *Missing from eCRF – n (%)^2^* | *xx (xx.x%)* | *xx (xx.x%)* |  |
|  |  |  |  |
|  |  |  |  |
| **Non-Invasive Stratum** |  |  |  |
| Initial testing |  |  |  |
| Mean | xx.x | xx.x | N/A |
| Median | xx | xx |  |
| *Missing from eCRF – n (%)^2^* | *xx (xx.x%)* | *xx (xx.x%)* |  |
| Follow up costs |  |  |  |
| Mean | xx.x | xx.x | x.xxx |
| Median | xx | xx |  |
| *Missing from eCRF – n (%)^2^* | *xx (xx.x%)* | *xx (xx.x%)* |  |
| Total costs |  |  |  |
| Mean | xx.x | xx.x | x.xxx |
| Median | xx | xx |  |
| *Missing from eCRF – n (%)^2^* | *xx (xx.x%)* | *xx (xx.x%)* |  |
|  |  |  |  |
|  |  |  |  |
| **Invasive Stratum** |  |  |  |
| Initial testing |  |  |  |
| Mean | xx.x | xx.x | N/A |
| Median | xx | xx |  |
| *Missing from eCRF – n (%)^2^* | *xx (xx.x%)* | *xx (xx.x%)* |  |
| Follow up costs |  |  |  |
| Mean | xx.x | xx.x | x.xxx |
| Median | xx | xx |  |
| *Missing from eCRF – n (%)^2^* | *xx (xx.x%)* | *xx (xx.x%)* |  |
| Total costs |  |  |  |
| Mean | xx.x | xx.x | x.xxx |
| Median | xx | xx |  |
| *Missing from eCRF – n (%)^2^* | *xx (xx.x%)* | *xx (xx.x%)* |  |
|  |  |  |  |

Figure 2 – Box and whisker plot for total medical costs over nine months, by Group


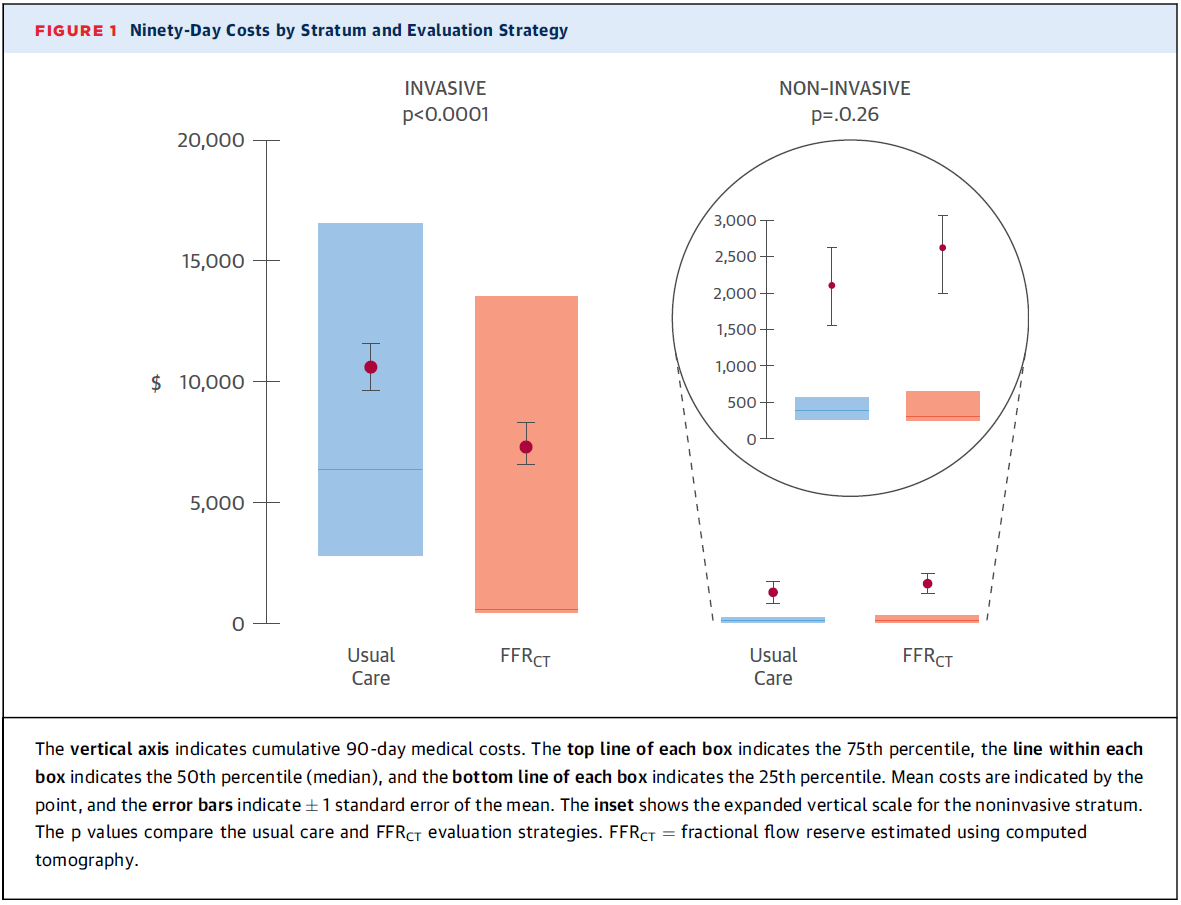


EXAMPLE FIGURE

(Figure taken from Hlatky et al, 2015 paper for the PLATFORM trial analyses)

Table 18 – Factors affecting total costs, in a log linear model

| **Resource** | **Coefficient** | **% Effect** | **P** |
| --- | --- | --- | --- |
|  |  |  |  |
| Random Assignment to FFR_CT_ | x.xxx | xx.x | x.xxx |
| First Test Preference | x.xxx | xx.x | x.xxx |
| Age | x.xxx | xx.x | x.xxx |
| Sex | x.xxx | xx.x | x.xxx |
| Typical Angina | x.xxx | xx.x | x.xxx |
| Diabetes | x.xxx | xx.x | x.xxx |
| Diamond Forrester Risk Score | x.xxx | xx.x | x.xxx |
| Invasive stratum * Random Assignment | x.xxx | xx.x | x.xxx |
|  |  |  |  |

## Secondary outcome analyses – secondary outcome 1 (clinical outcomes)

Table 19 – Major adverse cardiac events at 9 months summary

| **Characteristic** | **Reference Group**  **(n=XXX)** | **Test Group**  **(n=XXX)** | **Total**  **(n=XXX)** | **P** |
| --- | --- | --- | --- | --- |
|  |  |  |  |  |
| **Number of patients experiencing at least one major adverse cardiac event – n (%)^1^** | xx (xx.x%) | xx (xx.x%) | xx (xx.x%) | x.xxx |
| Number of patients who died from any cause – n (%)^1^ | xx (xx.x%) | xx (xx.x%) | xx (xx.x%) | x.xxx |
| Number of patients who have been hospitalised at least once for a cardiac event – n (%)^1^ | xx (xx.x%) | xx (xx.x%) | xx (xx.x%) | x.xxx |
| Number of patients experiencing at least one non-fatal MI – n (%)^1^ | xx (xx.x%) | xx (xx.x%) | xx (xx.x%) | x.xxx |
| Number of patients experiencing at least one non-fatal CVA – n (%)^1^ | xx (xx.x%) | xx (xx.x%) | xx (xx.x%) | x.xxx |
|  |  |  |  |  |
| **Number of patients experiencing more than one major adverse cardiac event – n (%)^1^** | xx (xx.x%) | xx (xx.x%) | xx (xx.x%) | N/A |
| Number of major adverse cardiac event per patient: |  |  |  |  |
| Mean | xx.x | xx.x | xx.x |  |
| Standard deviation | xx.xx | xx.xx | xx.xx |  |
| Median | xx | xx | xx |  |
| IQR | xx to xx | xx to xx | xx to xx |  |
| Range | xx to xx | xx to xx | xx to xx |  |
|  |  |  |  |  |
| Number of patients who have been hospitalised more than one for a cardiac event – n (%)^1^ | xx (xx.x%) | xx (xx.x%) | xx (xx.x%) | N/A |
| Number of cardiac hospitalisations per patient: |  |  |  |  |
| Mean | xx.x | xx.x | xx.x |  |
| Standard deviation | xx.xx | xx.xx | xx.xx |  |
| Median | xx | xx | xx |  |
| IQR | xx to xx | xx to xx | xx to xx |  |
| Range | xx to xx | xx to xx | xx to xx |  |
|  |  |  |  |  |
| Number of patients experiencing more than one non-fatal MI – n (%)^1^ | xx (xx.x%) | xx (xx.x%) | xx (xx.x%) | N/A |
| Number of non-fatal MI events per patient: |  |  |  |  |
| Mean | xx.x | xx.x | xx.x |  |
| Standard deviation | xx.xx | xx.xx | xx.xx |  |
| Median | xx | xx | xx |  |
| IQR | xx to xx | xx to xx | xx to xx |  |
| Range | xx to xx | xx to xx | xx to xx |  |
|  |  |  |  |  |
| Number of patients experiencing more than one non-fatal CVA – n (%)^1^ | xx (xx.x%) | xx (xx.x%) | xx (xx.x%) | N/A |
| Number of non-fatal CVA events per patient: |  |  |  |  |
| Mean | xx.x | xx.x | xx.x |  |
| Standard deviation | xx.xx | xx.xx | xx.xx |  |
| Median | xx | xx | xx |  |
| IQR | xx to xx | xx to xx | xx to xx |  |
| Range | xx to xx | xx to xx | xx to xx |  |
|  |  |  |  |  |

^1^ Denominator is the number of patients

Figure 3 – Kaplan Meier Plot for time to first major adverse cardiac event


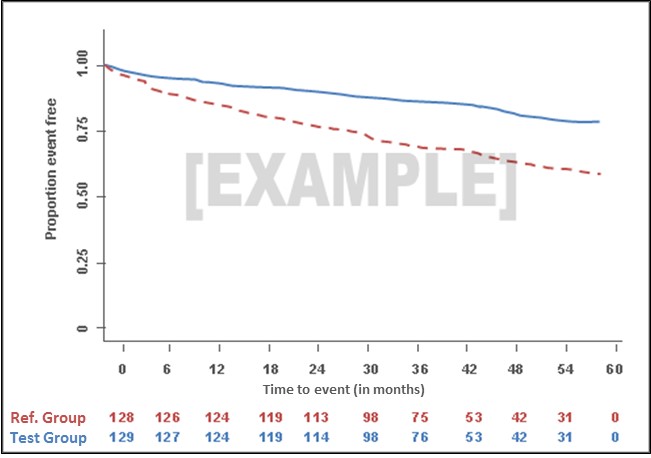


Table 20 – Cox regression model results for time to first major adverse cardiac events

| **Factor** | **Hazard ratio^1^** | **95% CI** | **P** |
| --- | --- | --- | --- |
|  |  |  |  |
| *Unadjusted* |  |  |  |
| Reference Group vs. Test Group | xx.xx | (x.xx to x.xx) | x.xxx |
|  |  |  |  |
| **Factor** | **Hazard ratio^1^** | **95% CI** | **P** |
|  |  |  |  |
| *Adjusted* |  |  |  |
| Reference Group vs. Test Group | xx.xx | (x.xx to x.xx) | x.xxx |
|  |  |  |  |
| Adjusted factor 1 | xx.xx | (x.xx to x.xx) | x.xxx |
| Adjusted factor 2 | xx.xx | (x.xx to x.xx) | x.xxx |
| … | … | … | … |
|  |  |  |  |

CI = Confidence Interval.

^1^ Hazard ratios above 1 represent a favourable outcome for the first category in the comparison.

Table 21 – Requirement for non-invasive cardiac investigations summary

| **Characteristic** | **Reference Group**  **(n=XXX)** | **Test Group**  **(n=XXX)** | **Total**  **(n=XXX)** | **P** |
| --- | --- | --- | --- | --- |
|  |  |  |  |  |
| **Number of patients experiencing at least one non-invasive cardiac investigation – n (%)^1^** | xx (xx.x%) | xx (xx.x%) | xx (xx.x%) | x.xxx |
| **Number of patients experiencing more than one non-invasive cardiac investigation – n (%)^1^** | xx (xx.x%) | xx (xx.x%) | xx (xx.x%) | N/A |
| Number of non-invasive cardiac investigations per patient: |  |  |  |  |
| Mean | xx.x | xx.x | xx.x |  |
| Standard deviation | xx.xx | xx.xx | xx.xx |  |
| Median | xx | xx | xx |  |
| IQR | xx to xx | xx to xx | xx to xx |  |
| Range | xx to xx | xx to xx | xx to xx |  |
|  |  |  |  |  |

^1^ Denominator is the number of patients

Figure 4 – Kaplan Meier Plot for time to first requirement for non-invasive cardiac investigations event

*[As above for* Figure 3 *but for requirement for non-invasive cardiac investigations]*

Table 22 – Cox regression model results for time to first requirement for non-invasive cardiac investigations

*[As above for Table 20 but for requirement for non-invasive cardiac investigations]*

Table 23 – Invasive coronary angiography summary

*[As above for Table 21 but for invasive coronary angiography at 9 months]*

Figure 5 – Kaplan Meier Plot for time to first invasive coronary angiography event

*[As above for* Figure 3 *but for invasive coronary angiography]*

Table 24 – Cox regression model results for time to first invasive coronary angiography

*[As above for Table 20 but for invasive coronary angiography]*

Table 25 – Revascularisation summary

*[As above for Table 21 but for requirement for revascularisations at 9 months]*

Figure 6 – Kaplan Meier Plot for time to first revascularisation event

*[As above for* Figure 3 *but for revascularisation]*

Table 26 – Cox regression model results for time to first revascularisations

*[As above for Table 20 but for revascularisations]*

Table 27 – Procedural complications summary

*[As above for Table 21 but for procedural complications at 9 months]*

Figure 7 – Kaplan Meier Plot for time to first procedural complication event

*[As above for* Figure 3 *but for procedural complications]*

Table 28 – Cox regression model results for time to first procedural complications

*[As above for Table 20 but for procedural complications]*

## Secondary outcome analyses – secondary outcome 2 (general wellbeing)

Table 29 – Change in EQ-5D-5L scores from baseline to 9 months

| **Characteristic** | **Reference Group**  **(n=XXX)** | **Test Group**  **(n=XXX)** | **P** |
| --- | --- | --- | --- |
|  |  |  |  |
| **Baseline** |  |  |  |
| Mean | xx.x | xx.x | N/A |
| Standard deviation | xx.xx | xx.xx |  |
| Median | xx | xx |  |
| IQR | xx to xx | xx to xx |  |
| Range | xx to xx | xx to xx |  |
|  |  |  |  |
| **Month 9** |  |  |  |
| Mean | xx.x | xx.x | N/A |
| Standard deviation | xx.xx | xx.xx |  |
| Median | xx | xx |  |
| IQR | xx to xx | xx to xx |  |
| Range | xx to xx | xx to xx |  |
|  |  |  |  |
| **Change (Month 9 – Baseline)** |  |  |  |
| Mean | xx.x | xx.x | x.xxx |
| Standard deviation | xx.xx | xx.xx |  |
| Median | xx | xx |  |
| IQR | xx to xx | xx to xx |  |
| Range | xx to xx | xx to xx |  |
|  |  |  |  |
|  |  |  |  |
| **Improvement in EQ-5D-5L observed from baseline to Month 9– n(%)^1^** |  |  |  |
| Yes | xx (xx.x%) | xx (xx.x%) | N/A |
| No | xx (xx.x%) | xx (xx.x%) |  |
| *Missing from eCRF – n (%)^2^* | *xx (xx.x%)* | *xx (xx.x%)* |  |
|  |  |  |  |
|  |  |  |  |
| **No improvement in EQ-5D-5L observed from baseline to Month 9 – n(%)^1^** |  |  |  |
| Yes | xx (xx.x%) | xx (xx.x%) | N/A |
| No | xx (xx.x%) | xx (xx.x%) |  |
| *Missing from eCRF – n (%)^2^* | *xx (xx.x%)* | *xx (xx.x%)* |  |
|  |  |  |  |
| **Deterioration in EQ-5D-5L observed from baseline to Month 9 – n(%)^1^** |  |  |  |
| Yes | xx (xx.x%) | xx (xx.x%) | N/A |
| No | xx (xx.x%) | xx (xx.x%) |  |
| *Missing from eCRF – n (%)^2^* | *xx (xx.x%)* | *xx (xx.x%)* |  |
|  |  |  |  |

^1^ Denominator is the number of patients with non-missing information available.

^2^ Denominator is the number of patients

Table 30 – Change in patient satisfaction from baseline to 9 months

*[As above for Table 29 but for patient satisfaction]*

Table 31 – Change in Seattle Angina from baseline to 9 months

*[As above for Table 29 but for Seattle Angina]*

Figure 8 - Change in QoL scores from baseline to 9 months summary


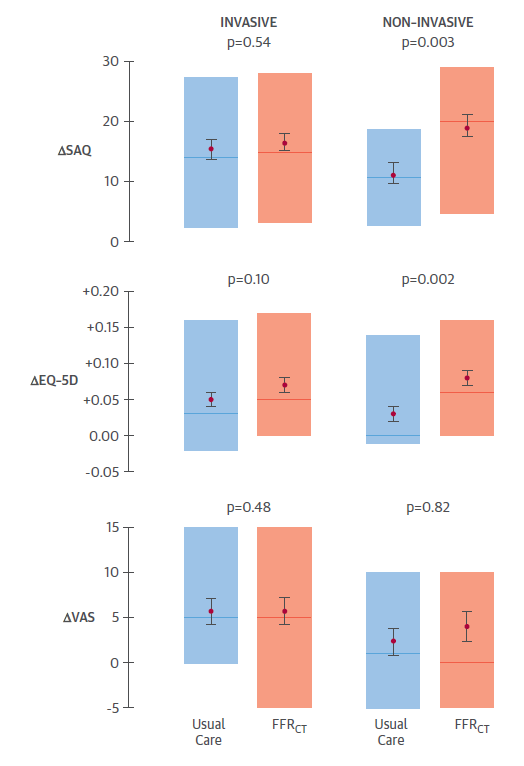


EXAMPLE FIGURE

(Figure taken from Hlatky et al, 2015 paper for the PLATFORM trial analyses)

Figure 9 – Box and whisker plot for time to definitive management plan, by group


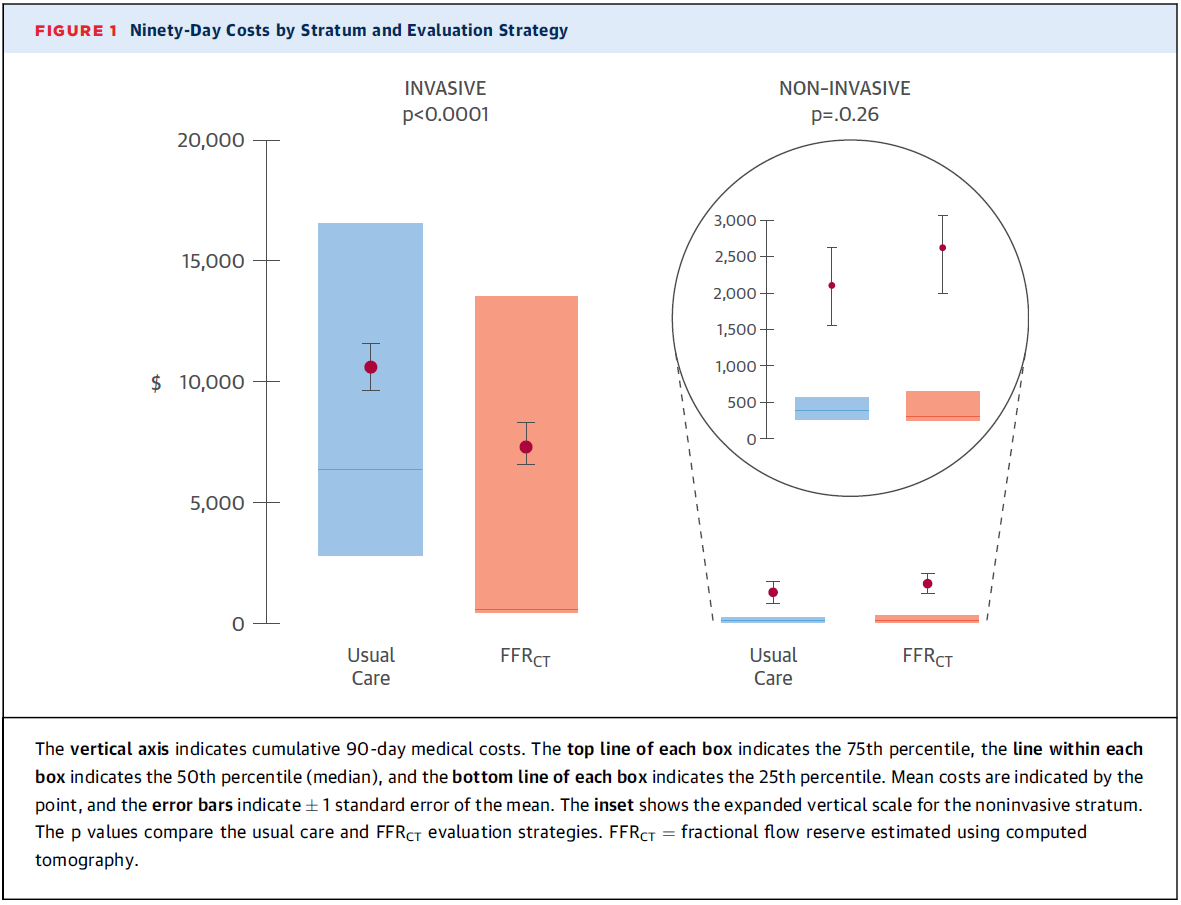


EXAMPLE FIGURE

(Figure taken from Hlatky et al, 2015 paper for the PLATFORM trial analyses)

Table 32 –Time to definitive management plan summary

| **Characteristic** | **Reference Group**  **(n=XXX)** | **Test Group**  **(n=XXX)** | **Total**  **(n=XXX)** | **P** |
| --- | --- | --- | --- | --- |
|  |  |  |  |  |
| **Time to definitive management plan -** |  |  |  |  |
| Mean | xx.x | xx.x | xx.x | x.xxx |
| Standard deviation | xx.xx | xx.xx | xx.xx |  |
| Median | xx | xx | xx |  |
| IQR | xx to xx | xx to xx | xx to xx |  |
| Range | xx to xx | xx to xx | xx to xx |  |
|  |  |  |  |  |

Figure 10 – Box and whisker plot for time to completion of initial management, by group

*[As above for Figure 9 but for time to completion of initial management]*

Table 33 – Time to completion of initial management summary

*[As above for Table 32 but for time to completion of initial management]*

Table 34 – Number of hospital attendances summary

| **Characteristic** | **Reference Group**  **(n=XXX)** | **Test Group**  **(n=XXX)** | **Total**  **(n=XXX)** |
| --- | --- | --- | --- |
|  |  |  |  |
| **Number of hospital attendances -** |  |  |  |
| Mean | xx.x | xx.x | xx.x |
| Standard deviation | xx.xx | xx.xx | xx.xx |
| Median | xx | xx | xx |
| IQR | xx to xx | xx to xx | xx to xx |
| Range | xx to xx | xx to xx | xx to xx |
|  |  |  |  |

Table 35 – Number of hospital attendances ANCOVA

| **Characteristic** | **Statistic** | | | |
| --- | --- | --- | --- | --- |
| **Reference Group vs. Test Group** | | | | |
| **Least Squares Means** | **Estimate** | **Difference** | **95% CI of LS Mean** | **Two-sided**  **p-value^1^** |
| **Reference Group** | **xxx.xx** | **xx.xx** | **(xxx.xx, xxx.xx)** | **x.xxx** |
| **Test Group** | **xxx.xx** |  |  |  |
|  |  |  |  |  |
| **Model Coefficients** | **Estimate** | **95% CI** | | **p-value** |
| Reference Group | xxx.xx | (xxx.xx, xxx.xx) | | x.xxx |
| Test Group | xxx.xx | (xxx.xx, xxx.xx) | | x.xxx |
|  |  |  | |  |
| Intercept | xxx.xx | (xxx.xx, xxx.xx) | | x.xxx |
|  |  |  | |  |
| XXXX | xxx.xx | (xxx.xx, xxx.xx) | | x.xxx |
| XXXX | xxx.xx | (xxx.xx, xxx.xx) | | x.xxx |
| … | … | … | | … |
|  |  |  | |  |

CI = Confidence interval; ANCOVA = Analysis of covariance.

Table 36 – Number of working days lost summary (for those who are employed at baseline only)

*[As above for* Table 36 *but for working days lost]*

Table 37 – Number of working days lost ANCOVA (for those who are employed at baseline only)

*[As above for Table 37 but for working days lost]*

## Safety reporting

Table 38 – CTCA Related Serious Adverse Events (Test group only)

| **Characteristic** | **Test Group**  **(n=XXX)** |
| --- | --- |
|  |  |
| **Did patient undergo CTCA? – n (%)^1^** |  |
| Yes | xx (xx.x%) |
| No | xx (xx.x%) |
| *Missing from eCRF – n (%)^2^* | *xx (xx.x%)* |
|  |  |
|  |  |
| **CTCA related Serious Adverse Event experienced– n(%)^1^** |  |
| Anaphylaxis | xx (xx.x%) |
| Other contrast allergy | xx (xx.x%) |
| Acute renal failure | xx (xx.x%) |
| Bradycardia requiring treatment | xx (xx.x%) |
| Any incidental CTCA findings noted: |  |
| Yes | xx (xx.x%) |
| Any incidental CTCA findings investigated: |  |
| Yes | xx (xx.x%) |
| Incidental findings and any further investigations: |  |
| XXXX | xx (xx.x%) |
| XXXX | xx (xx.x%) |
| … | … |
| No | xx (xx.x%) |
| No | xx (xx.x%) |
|  |  |

^1^ Denominator is the number of patients with non-missing information available.

^2^ Denominator is the number of patients

Table 39 – Mortality information

| **Characteristic** | **Reference Group**  **(n=XXX)** | **Test Group**  **(n=XXX)** | **Total**  **(n=XXX)** |
| --- | --- | --- | --- |
|  |  |  |  |
| **Patient Died – n (%)^1^** | xx (xx.x%) | xx (xx.x%) | xx (xx.x%) |
| Cause of death: |  |  |  |
| XXXX | xx (xx.x%) | xx (xx.x%) | xx (xx.x%) |
| XXXX | xx (xx.x%) | xx (xx.x%) | xx (xx.x%) |
| … | … | … | … |
|  |  |  |  |
|  |  |  |  |
| **Post mortem performed – n (%)^1^** |  |  |  |
| Yes | xx (xx.x%) | xx (xx.x%) | xx (xx.x%) |
| Report available | xx (xx.x%) | xx (xx.x%) | xx (xx.x%) |
| Report not available | xx (xx.x%) | xx (xx.x%) | xx (xx.x%) |
| No | xx (xx.x%) | xx (xx.x%) | xx (xx.x%) |
|  |  |  |  |

^1^ Denominator is the number of patients with non-missing information available.

^2^ Denominator is the number of patients

Table 40 – SAE Listings

*[Example table not shown]*

# References

*Please see FORECAST protocol v3 for a full list of clinical references.*

# SAP revision history

| **Version number** | **Revision history** | **Author** | **Date** |
| --- | --- | --- | --- |
| 0.1 | First draft created following meeting with Mark Hlatky on 25-Apr-2019 | Tom Maishman | 02-May-2019 |
| 0.2 | Notes added following meeting with Mark Hlatky on 9-May-2019 | Tom Maishman | 23-May-2019 |
| 0.3 | Updates made following meeting with Mark Hlatky on 9-May-2019 | Tom Maishman | 28-May-2019 |
| 0.4 | Updates made following meeting with Nick Curzen and Mark Hlatky on 20-Jun-2019, and following the TSC and corresponding comments on 27-Jun-2019 | Kayleigh Hill | 08-Jul-2019 |
| 0.5 | Updates made following comments from Nick Curzen | Kayleigh Hill | 20-Aug-2019 |
| 1 | Finalised version created using v0.5 | Kayleigh Hill (and Tom Maishman) | 27-Aug-2019 |
