# Supplementary material for: Fractional flow reserve derived from computed tomography coronary angiography in the assessment and management of stable chest pain: the FORECAST randomized trial
Source: Eur Heart J. 2021 Jul 16;42(37):3844–52. doi: 10.1093/eurheartj/ehab444 (PMC8648068; doi:10.1093/eurheartj/ehab444)
Supplement: ehab444_Supplementary_Data [file ehab444_supplementary_data.zip › ehab444-suppl_data/FORECAST APPENDIX A.docx]

| 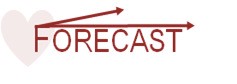The **FORECAST** Trial | |
| --- | --- |
| **F**ractional Fl**o**w **Re**serve Derived from Computed Tomography **C**oronary **A**ngiography in the Assessment and Management of **St**able Chest Pain | |
| **Version 3.0 20-Mar-2019** | |
|  | |
| **SPONSOR:** University Hospital Southampton | |
| **COORDINATING CENTRE:**  Southampton Clinical Trials Unit | |
|  | |
|  |  |
| Ethics reference number: | 17/SC/0490 |
| Sponsor reference number: | RHM CAR0524 |
| NCT Number | NCT03187639 |
| Funder reference number: | Heartflow |
|  |  |
| **IRAS project ID number: 231037** | |
|  | |

**
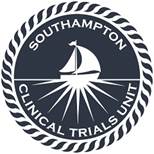
APPENDIX A**


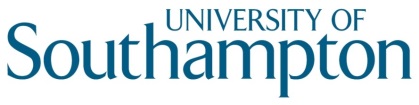

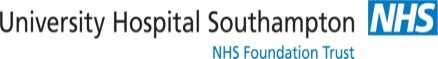


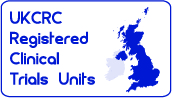


|  |  |  |  |
| --- | --- | --- | --- |

| **Protocol authorised by:** | | | |
| --- | --- | --- | --- |
|  |  |  |  |
| **Name:** | Prof. Nick Curzen | **Role:** | Chief Investigator |
|  |  |  |  |
| **Signature:** |  | **Date:** |  |
|  |  |  |  |
| **Name:** | Prof. Gareth Griffiths | **Role:** | Director of SCTU |
|  |  |  |  |
| **Signature:** |  | **Date:** |  |
|  |  |  |  |
| **Name:** | Dr Mikayala King | **Role:** | On behalf of Sponsor |
|  |  |  |  |
| **Signature:** |  | **Date:** |  |
|  |  |  |  |

| **MAIN Study ConTact** | | | | | | | | |
| --- | --- | --- | --- | --- | --- | --- | --- | --- |
| Chief Investigator and Medical Expert: | | | Prof. Nick Curzen | | | | | |
|  | E Level North Wing University  Hospital Southampton NHS FT  Southampton  SO16 6YD | | | | Tel:  Email: | | 02381 204972  Nick.Curzen@uhs.nhs.uk | |
|  |  |  |  |  |  |  |  |  |
| **Study Coordination Centre** | | | | | | | | |
| For general study and clinical queries e.g. participant queries, study supplies, data collection, please contact in the first instance: | | | | | | | | |
| FORECAST Trial Manager | | | | Tel: | | 023 8120 5538 | | |
|  | | | | Email: [forecast@soton.ac.uk](mailto:forecast@soton.ac.uk)  FORECAST Mobile : **07717 715757** | | | | |
| Address: | | Southampton Clinical Trials Unit  Southampton General Hospital  Tremona Road  SOUTHAMPTON  SO16 6YD | | Tel:  Fax:  Email:  Web: | | 023 8120 5154  0844 774 0621  [ctu@soton.ac.uk](mailto:ctu@soton.ac.uk)  [www.southampton.ac.uk/ctu](http://www.southampton.ac.uk/ctu) | | |
| **Sponsor** | | | | | | | | |
| University Hospital Southampton NHS Foundation Trust is the research sponsor for this study. For further information regarding sponsorship conditions, please contact the Director of Research and Development at: | | | | | | | | |
| Address: | | R&D Department | | Tel:  Fax:  Web: | | | | 023 8120 4989  023 8120 8678  www.uhs.nhs.uk |
| University Hospital Southampton NHS Foundation Trust  SGH, Level E, Laboratory & Pathology Block, SCBR, MP 138  Tremona Road  SOUTHAMPTON  SO16 6YD | | | | | | | |  |
| **CO-INVESTIGATOR(S)** | | | | | | | | |
| Co-Investigators can be contacted via the Trial Coordination Centre.  Colin Berry BSc MBBS PhD FRCP  Mark Hlatky BS MD | | | | | | | | |
| **FUNDER**  This study is funded by HeartFlow Inc ® as an unrestricted investigator led study. | | | | | | | | |
| **Protocol Information**  This protocol describes the FORECAST Trial study and provides information about procedures for entering participants. The protocol should not be used as a guide for the treatment of other non- study participants; every care was taken in its drafting, but corrections or amendments may be necessary. These will be circulated to investigators in the study, but sites entering participants for the first time are advised to contact Southampton Clinical Trials Unit to confirm they have the most recent version.  **Compliance**  This study will adhere to the principles of Good Clinical Practice (GCP). It will be conducted in compliance with the protocol, the current Data Protection Regulations and all other regulatory requirements, as appropriate. | | | | | | | | |

**Table of Contents**

LIST OF ABBREVIATIONS 5

KEYWORDS 5

STUDY SYNOPSIS 6

[SCHEDULE OF OBSERVATIONS AND PROCEDURES 8](#_Toc490738791)

[1 INTRODUCTION 9](#_Toc490738792)

[1.1 Background 9](#_Toc490738793)

[1.2 Rationale and Risk Benefits For Current study 13](#_Toc490738794)

[2 STUDY OBJECTIVES AND ENDPOINTS 14](#_Toc490738795)

[3 STUDY DESIGN 14](#_Toc490738796)

[3.1 Definition of End of Study 14](#_Toc490738797)

[4 SITE INITIATION 15](#_Toc490738798)

[5 SELECTION AND ENROLMENT OF PARTICIPANTS 15](#_Toc490738799)

[5.1 eLGIBILITY SCREENING 15](#_Toc490738800)

[5.2 Consent 15](#_Toc490738801)

[5.3 Inclusion Criteria 16](#_Toc490738802)

[5.4 Exclusion Criteria 16](#_Toc490738803)

[5.5 Screening failures 16](#_Toc490738804)

[5.6 Randomisation Procedures 17](#_Toc490738805)

[6 STUDY OBSERVATIONS AND PROCEDURES 17](#_Toc490738806)

[6.1 data entry 17](#_Toc490738807)

[6.2 Study Procedures 17](#_Toc490738808)

[6.3 Follow up 19](#_Toc490738809)

[6.4 AssessmEnt window 19](#_Toc490738810)

[6.5 Deviations and serious Breaches 19](#_Toc490738811)

[6.6 Withdrawal 20](#_Toc490738812)

[7 SAFETY 20](#_Toc490738813)

[7.1 Definitions 20](#_Toc490738814)

[7.2 Seriousness 21](#_Toc490738815)

[7.3 Causality 22](#_Toc490738816)

[7.4 Expectedness 22](#_Toc490738817)

[7.5 Reporting Procedures 23](#_Toc490738818)

[7.6 SCTU Responsibilities For Safety Reporting to rec 24](#_Toc490738819)

[8 STATISTICS AND DATA ANALYSES 24](#_Toc490738820)

[8.1 Method of randomisation 24](#_Toc490738821)

[8.2 Sample size 24](#_Toc490738822)

[8.3 Interim Analysis 25](#_Toc490738823)

[8.4 Statistical Analysis Plan (SAP) 25](#_Toc490738824)

[9 REGULATORY 26](#_Toc490738825)

[9.1 Clinical Trial Authorisation 26](#_Toc490738826)

[10 ETHICAL CONSIDERATIONS 26](#_Toc490738827)

[10.1 specific ethical considerations 26](#_Toc490738828)

[10.2 ETHICAL APPROVAL 27](#_Toc490738829)

[10.3 INFORMED CONSENT PROCESS 27](#_Toc490738830)

[10.4 CONFIDENTIALITY 27](#_Toc490738831)

[11 SPONSOR 27](#_Toc490738832)

[11.1 INDEMNITY 27](#_Toc490738833)

[11.2 FUNDING 27](#_Toc490738834)

[11.3 AUDITS AND INSPECTIONS 28](#_Toc490738835)

[12 STUDY OVERSIGHT GROUPS 28](#_Toc490738836)

[12.1 Trial Management Group (TMG) 28](#_Toc490738837)

[12.2 trial steering committee (tsc) 28](#_Toc490738838)

[13 DATA MANAGEMENT 29](#_Toc490738839)

[14 MONITORING 29](#_Toc490738840)

[14.1 Central Monitoring 29](#_Toc490738841)

[14.2 Clinical Site Monitoring 30](#_Toc490738842)

[14.3 Source Data 30](#_Toc490738843)

[15 RECORD RETENTION AND ARCHIVING 30](#_Toc490738844)

[16 PUBLICATION POLICY 30](#_Toc490738845)

[17 REFERENCES 32](#_Toc490738846)

**List of Abbreviations**

| AE | Adverse Event |
| --- | --- |
| CRF | Case Report Form |
| CTCAE | Common Terminology Criteria for Adverse Events |
| DMEC | Data Monitoring and Ethics Committee |
| GCP | Good Clinical Practice |
| IDMC | Independent Data Monitoring Committee |
| ISF | Investigator Site File |
| MHRA | Medicines and Healthcare Products Regulatory Agency |
| REC | Research Ethics Committee |
| SAE | Serious Adverse Event |
| SCTU | Southampton Clinical Trials Unit |
| TMF | Trial Master File |
| TMG | Trial Management Group |
| TSC | Trial Steering Committee |
| CP | Chest Pain |
| CAD | Coronary Artery Disease |
| RMI | Reversible Myocardial Ischaemia |
| MI | Myocardial Infarction |
| OMT | Optimal Medical Therapy |
| IC | Invasive Coronary Angiography |
| CTCA | CT Coronary Angiography |
| FFR | Fractional Flow Reserve |
| FFRCT | Non-invasive technique using CT to determine Fractional Flow Reserve |
| QOL | Quality of Life |
| RACPC | Rapid Access Chest Pain Clinic |
| SAQ-7 | Seattle Angina Questionnaire – Short Form |
| EQ-5D-5L | Quality of life questionnaire |
| PCI | Percutaneous Coronary Intervention |
| CABG | Coronary Artery Bypass Graft |
| MRI | Magnetic Resonance Imaging |

**Keywords**

Stable chest pain, CT coronary angiography, fractional flow reserve, coronary artery disease, reversible myocardial ischaemia, myocardial infarction

**STUDY SYNOPSIS**

| **Short title/Acronym:** | The FORECAST Trial |
| --- | --- |
| **Full title:** | **F**ractional Fl**O**w **Re**serve Derived from Computed Tomography **C**oronary **A**ngiography in the Assessment and Management of **St**able Chest Pain |
|  |  |
| **Study Phase:** | **IV** |
| **Population:** | Patients presenting in secondary care to Rapid Access Chest Pain Clinic with recent onset stable chest pain |
| **Primary Objective:** | To determine whether, in a population of patients presenting to RACPC, routine FFR_CT_ as a default test is superior, in terms of resource utilisation at 9 months, when compared to routine clinical pathway algorithms recommended by NICE CG95. |
| **Secondary Objective:** | 1. To compare clinical outcomes between the two groups at 9 months 2. To compare the effect on general wellbeing between the two groups at 9 months |
| **Rationale:** | Current algorithms for assessment of stable new onset chest pain are heterogeneous and variably focus on either anatomical or physiological parameters. FFR_CT_ offers simultaneous assessment of both anatomy and ischaemia and therefore may have advantage in terms of resource utilisation. |
| **Study Design:** | Randomised Controlled Trial |
| **Sample size :** | 1400 |
| **Treatment/Intervention:** | Routine CT coronary angiography and FFR_CT_ for assessment of new onset chest pain in the setting of Rapid Access Chest Pain Clinics |
|  |  |
| **URL for Database:** | <https://login.imedidata.com/login> |
| **URL for randomisation:** | https://prod.tenalea.net/stn/dm |
|  |  |
| **Primary Study Endpoint:** | Resource utilisation derived from:   - Requirement for non-invasive cardiac investigations - Invasive angiography - Revascularisation (including PCI & CABG) - Hospitalisation for cardiac event (including MI/unstable angina/revascularisation/heart failure/arrhythmia) - Cardiac medications |
| **Secondary Study Endpoints:** | Major adverse cardiac and cerebrovascular events (MACCE) including: all-cause mortality/non-fatal MI/non-fatal stroke/   - Composite clinical endpoint to include death, MI, stroke, unplanned revascularisation, hospitalisation for cardiac cause - Complications of invasive procedures - QoL - Patient satisfaction - Time to definitive management plan - Time to completion of intended management - Number of hospital attendances - Working days lost |
| **Total Number of Sites:** | 10-15 Secondary Care |

**STUDY SCHEMA**

Patients present at Rapid Access Chest Pain Clinic (RACPC)

who require a cardiac test

Consent & Randomisation (1:1)

Routine Assessment

(Reference Group)

FFR_CT_

Assessment

(Test Group)

CT Angiography Assessment

FFR_CT_

Treat as per supervising physician preference

Coronary stenosis ≥40% in at least 1 major epicardial vessel of stentable/graftable diameter?

Yes

No

Management strategy based on FFR_CT_ results.

Management strategy based on NICE Chest Pain of Recent Onset Guidance

(CG95)

All Patients Follow Up – 3 Months

All Patients Follow Up –9 Months

#

## SCHEDULE OF OBSERVATIONS AND PROCEDURES

| Visit: | Screening/  Baseline | Diagnostic Testing | 3 Month FU^3^ | 9 Month FU^3^ |
| --- | --- | --- | --- | --- |
| Inclusion/Exclusion | X |  |  |  |
| Informed Consent | X |  |  |  |
| General Medical History and Cardiovascular Risk Factors | X |  |  |  |
| Demographics, Height and Weight | X |  |  |  |
| Randomisation | X |  |  |  |
| CTA |  | X |  |  |
| FFR_CT_ Analysis^1^ |  | X |  |  |
| Resource Utilisation^2^ |  |  | X | X |
| Concurrent medication | X |  | X | X |
| Seattle Angina Questionnaire score | X |  | X | X |
| EQ-5D – 5L | X |  | X | X |
| Treatment Satisfaction Questionnaire | X |  | X | X |
| Clinical Events |  |  | X | X |
| MACCE ( All-cause mortality/, non-fatal MI, non-fatal stroke but not revascularisation/ |  |  |  | X |
| Trial related SAEs/ Safety Endpoints |  | X |  |  |

^1^For patients in test group with qualifying CTA results. CTA given where appropriate in ‘Routine Assessment’ group following NICE guidelines.

^2^To include all cardiac-related medications, tests, hospital visits, time off work.

^3^ Follow up will take place at 3 months and 9 months following randomisation, within a timeframe of ±15 days from the due date.

**NB:** The Participant is free to withdraw consent at any time without providing a reason. When withdrawn, the participant will continue to receive standard clinical care. Follow up data will continue to be collected (unless the participant has specifically stated that they do not want this to happen).

# INTRODUCTION

## Background

The optimal algorithm with which to assess and then manage patients who present with stable chest pain (CP) that might represent angina (i.e. ischaemia secondary to coronary artery disease (CAD)) is not clear. In such patients, there may be both symptomatic and prognostic benefit to knowing whether they have either (a) significant coronary artery atheroma (i.e. *the anatomy* of their coronary arteries) or (b) reversible myocardial ischaemia (RMI) (i.e. assessing *the physiology* of the artery(ies) as a surrogate) or (c) both. Evidence demonstrates that it is useful to know about the presence of atheroma because the medium and long-term natural history of this disease process can be altered by the administration of disease-modifying medical therapy.^[[1]](#endnote-1)^ However, it is now accepted, based upon both observational data and randomised trials, that it is the presence and extent of RMI that most closely correlates with the near term risk of acute myocardial infarction (MI) and that mechanical intervention by coronary revascularisation is effective both for symptoms and prognosis in the population with RMI both at patient and lesion-level.^[[2]](#endnote-2)^ ^[[3]](#endnote-3)^ ^[[4]](#endnote-4)^ Thus, RMI is generally considered to represent the principal target for revascularisation strategies whereas optimal medical therapy (OMT) can improve prognosis regardless of the existence of RMI in some patients.^[[5]](#endnote-5)^ ^[[6]](#endnote-6)^ Based on this, it is clear that both coronary anatomy and physiological assessment (as a surrogate of RMI) are of value in the diagnosis and management of patients presenting with stable cardiac-sounding CP.

Current tests used in patients presenting with stable CP assess either coronary anatomy or physiology: non-invasive stress tests (echo/nuclear perfusion/CMR) assess for RMI.^[[7]](#endnote-7)^ By contrast, invasive coronary angiography (IC) and CT coronary angiography (CTCA) are tests of anatomy. The advent of the invasive pressure wire has provided an accurate tool for the direct assessment of coronary physiology at the time of IC and until recently was therefore unique in allowing for assessment of both anatomy and RMI at the same time. However, the wide range of choice offered by this menu of non-invasive and invasive tests cannot hide the fact that there are important limitations of them all. Specifically, the non-invasive stress tests are highly specialised, expensive, not freely available to patients in all hospitals and associated with significant waiting time in the UK. Invasive angiography carries small but definite risk, is expensive and again not freely available. Furthermore, the “yield” in terms of finding significant CAD when IC is used as the front line screening tool for patients with new onset stable CP is relatively low. For example, in 398,978 patients in one large US registry undergoing diagnostic coronary angiography, only 37.6% had obstructive CAD (defined as 50% or more of the diameter of the left main coronary artery or 70% or more of the diameter of a major epicardial coronary artery).^[[8]](#endnote-8)^ Even in patients in whom CAD is identified, there is a well described discrepancy between the anatomical appearance and physiological evidence of RMI in a variety of clinical studies and this effectively calls into question whether an anatomy-based test is an appropriate tool with which to define the management of such patients. For example, in RIPCORD, the availability of FFR for all vessels of stentable size altered the assessment of the number and location of “significant” stenosis in 32% with a resultant change in the management plan in 26% of the 200 patients undergoing diagnostic angiography for stable chest pain. ^[[9]](#endnote-9)^ A similar mismatch between the anatomical and functional assessments of significance of coronary lesions is demonstrated in a wide variety of clinical studies, some of which are shown here.^[[10]](#endnote-10)^ ^[[11]](#endnote-11)^


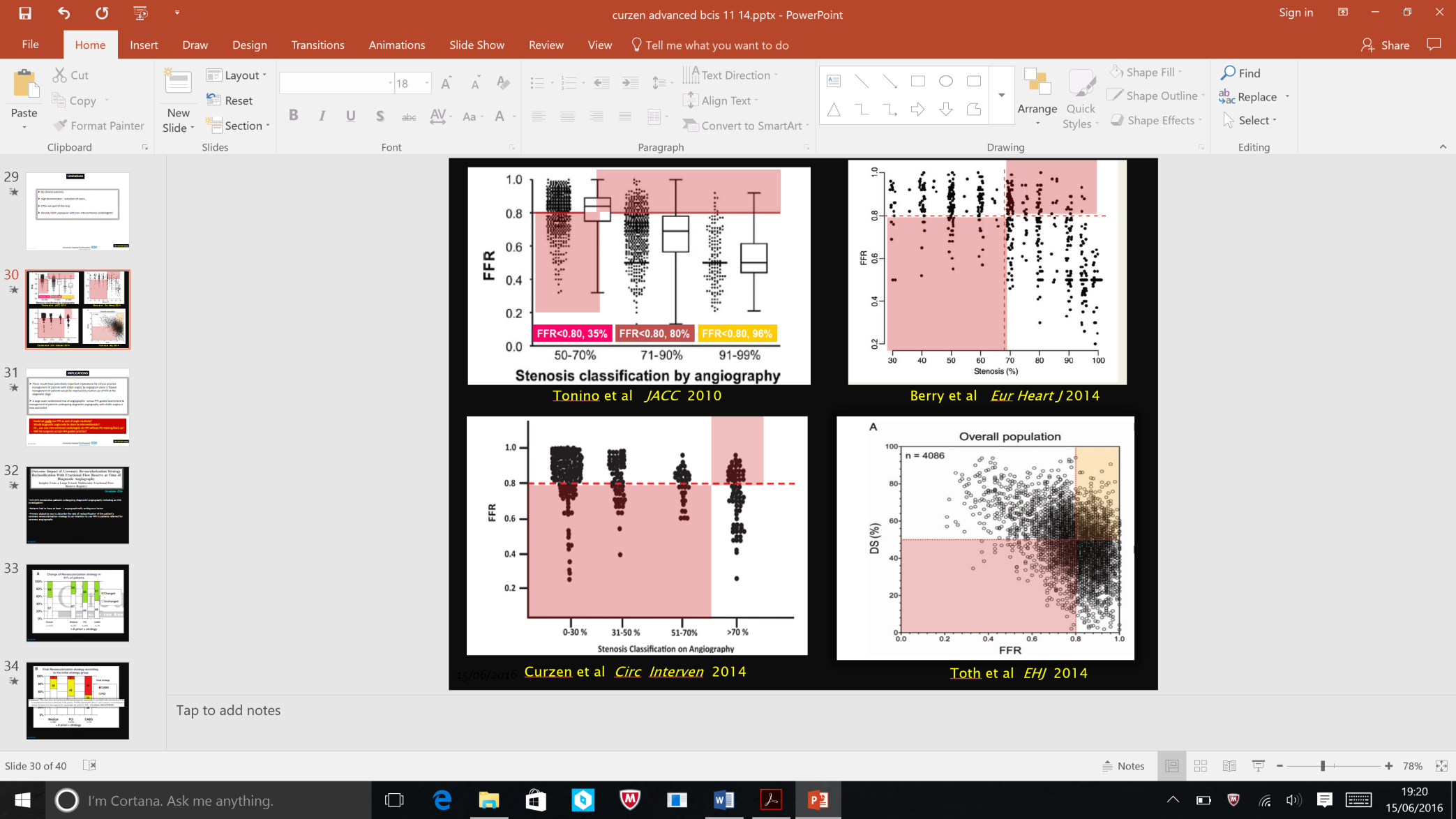


The inherent limitations of both non-invasive and invasive tests for the accurate diagnosis and management of each patient presenting in this context is reflected in the heterogeneity of the management in the real world. This is well demonstrated by the 2010 NICE Guideline for Chest Pain of Recent Onset^[[12]](#endnote-12)^ in which the recommended algorithms for assessment and management of this cohort are complex and disparate (see below). Furthermore, one consequence of a true negative stress test is that we do not establish if the patient has coronary atheroma which may represent an indication for disease-modifying therapy. By contrast, a true positive stress test inevitably then results in referral to the waiting list for IC, thus delaying the time from assessment to definitive diagnosis and management plan significantly. Conversely, a referral directly for IC in the absence of objective evidence of RMI subjects the patient to a stressful procedure that carries potential for serious complication merely to establish in many cases that there is no substrate for the CP presentation.

The ideal screening test for patients with stable CP with which to determine the coronary anatomy and physiology would be non-invasive, quick, simple, widely accessible, accurate (using a combination of IC + FFR as the gold standard reference) and as cheap as possible. However, we can refine the desirable features of such a screening test even more precisely. Firstly, our ideal test would avoid the expense, inconvenience and risk of invasive investigation in patients who turn out to have no important atheroma, whilst being able, conversely, to identify even non-flow limiting atheroma in patients who may therefore benefit from disease-modifying medical therapy. Secondly, an ideal screening test would incorporate the value, in terms of patient outcome, of physiological assessment that has been demonstrated in the clinical trial data accumulated using the coronary pressure wire. Specifically, 2 basic principles of management can be applied at the diagnostic stage if it includes a physiological assessment. Firstly, that stenting an anatomical lesion deemed angiographically significant which is FFR negative is associated with a worse outcome than medical therapy alone, as demonstrated by both DEFER^[[13]](#endnote-13)^ and FAME.^[[14]](#endnote-14)^ Secondly, that treating pressure wire positive lesions with OMT alone is associated with a worse clinical outcome than PCI plus OMT is just as important, as demonstrated in FAME 2.^[[15]](#endnote-15)^ ^[[16]](#endnote-16)^ In summary, the ideal screening test for patients with stable chest pain needs to be able to deliver information about RMI (or a physiological surrogate of it), as well as anatomy.

FFR_CT_ represents a plausible candidate for a screening test in this population. FFR_CT_ is a novel method for computer-derived estimation of coronary physiology from CT angiography data, and early validation studies with regard to its diagnostic performance are promising (see below). The original development and availability of computerized tomographic coronary angiography (CTA) has introduced this non-invasive assessment of the presence and extent of CAD as a useful additional option for the investigation of patients with suspected stable angina.^[[17]](#endnote-17)^ ^[[18]](#endnote-18)^ Recent data from the SCOT-HEART randomised trial, for example, highlights the diagnostic value of this technique in such a population.^[[19]](#endnote-19)^ However, in the PROMISE trial, which randomised 10,003 patients with symptoms suggestive of CAD to stress testing or CTA, there was almost 50% more ICA performed in the CTA group without any difference in clinical outcome.^[[20]](#endnote-20)^ Importantly, most revascularisations performed in the CTA group had no objective evidence of myocardial ischaemia. This highlights the potential limitation of anatomy-based screening in patients with symptoms thought to be due to myocardial ischaemia.

Recently, using sophisticated image assessment, computational fluid dynamics, and computer modelling, it has become possible to model FFR from the data obtained from CTA, thus known as FFR_CT_.^[[21]](#endnote-21)^ A series of validation studies have assessed the diagnostic accuracy of this technique,^[[22]](#endnote-22)^ ^[[23]](#endnote-23)^ including most recently the NXT trial, which demonstrated superior accuracy of CTA plus FFR_CT_ versus CTA alone, using an invasive FFR of <0.80 as the reference. Specifically, the area under the receiver-operating characteristic curve was 0.9 for FFR_CT_ versus 0.81 for CTA alone (p=0.008), using invasive FFR < 0.8 as the reference.

Recent data describe the clinical utility of the FFR_CT_ model in patients with chest pain. The PLATFORM study recruited 584 patients with new onset chest pain who were assigned to either conventional assessment or CTA/FFR_CT._^[[24]](#endnote-24)^ The primary endpoint was the percentage of those undergoing ICA in whom there was no significant CAD as defined by no more than 50% stenosis or FFR less than 0.8. Among those with intended ICA, no obstructive CAD was found at ICA in 12% of those in the FFR_CT_ arm and in 73% of the usual care arm (p<0.0001). Furthermore, ICA was cancelled in 61% of patients after FFR_CT_ results were available. The conclusion was that FFR_CT_ was a feasible and safe alternative to ICA that was associated with a significantly lower rate of ICA showing obstructive CAD.

Further, PLATFORM included a pre-specified economic and quality of life analysis. In the planned invasive stratum, mean costs were 32% lower among the FFR_CT_ patients than among the usual care patients ($7,343 vs. $10,734 p<0.0001). In the non-invasive stratum, mean costs were not significantly different between the FFR_CT_ patients and the usual care patients ($2,679 vs. $2,137, p=0.26).^[[25]](#endnote-25)^ Each QOL score improved in the overall study population (p<0.0001). In the non-invasive stratum, QOL scores improved more in FFR_CT_ patients than in usual care patients: SAQ 19.5 vs. 11.4, p=0.003; EQ-5D 0.08 vs. 0.03, p=0.002; and VAS 4.1 vs 2.3, p=0.82. In the invasive cohort, the improvements in QOL were similar in the FFR_CT_ and usual care patients. The conclusions were as follows. An evaluation strategy based on FFR_CT_ was associated with less resource use and lower costs within 90 days than evaluation with invasive coronary angiography. Evaluation with FFR_CT_ was associated with greater improvement in quality of life than evaluation with usual non-invasive testing.

These results are encouraging for the candidacy of FFR_CT_ as a primary screening test for patients with stable chest pain. However, although promising, the observational PLATFORM study does not provide an adequate level of evidence to justify a shift towards routine FFR_CT_ as a default algorithm in clinical practice. *Specifically, PLATFORM does not provide the level of evidence from a suitably powered randomised controlled trial that is routinely required in order to trigger a change in clinical guidelines.*

## Rationale and Risk Benefits For Current study

This trial aims to test the hypothesis that FFR_CT_, used as the default screening tool for patients presenting with recent onset stable chest pain, would be associated with (i) shorter rapid time period between initial consultation and definitive management plan; (ii) better patient experience; (iii) lower overall use of resources.

The UK is well suited to test this hypothesis because of its well-established system of Rapid Access Chest Pain Clinics (RACPC). The majority of patients presenting with stable CP that is of suspected cardiac origin are referred to such clinics, with a mandated access time within 2 weeks. The majority of such clinics work to the algorithm recommended in the NICE guidelines for Chest Pain of Recent Onset (March 2010). Within this guideline, patients are stratified according to their risk profile and pre-test likelihood of CAD to outcomes that include discharge, stress test, CTCA, Ct coronary calcium score and IC. (See Appendix 1)

Given the relative streamlining of this initial assessment of such patients throughout the country, it facilitates a comparison of strategies in FORECAST.

The 2 strategies for the FORECAST trial are:

[A] TEST: all patients undergo CTCA± FFR_CT_ as the default test, assuming they have no pre-specified contraindications to CT angiography. The result of the CTCA will determine whether the FFRCT will be performed. Results of the FFR_CT_ will be conveyed to the supervising physician within 24 hours and will be used to determine the subsequent management plan.

[B] REFERENCE: all patients will be assessed and managed exactly as they are usually treated by that centre and that RACP using the local algorithms interpreted from the NICE Chest Pain of Recent Onset Guidance.

# STUDY OBJECTIVES AND ENDPOINTS

|  | Objective | Endpoint used to evaluate |
| --- | --- | --- |
| Primary | To determine whether, in a population of patients presenting to RACPC, routine FFR_CT_ as a default test is superior, in terms of resource utilisation, when compared to routine clinical pathway algorithms recommended by NICE. | Resource utilisation at 9 months :  a) Requirement for non-invasive cardiac investigations  b) Invasive angiography  c) Revascularisation (including PCI & CABG)  d) Hospitalisation for cardiac event (including MI/unstable angina/revascularisation/heart failure/arrhythmia)  e) Cardiac medications |
| Secondary | To compare clinical outcomes between the two groups at 9 months. | 1. MACCE (All-cause mortality, non-fatal MI, non-fatal stroke. 2. Composite clinical endpoint to include death, MI, stroke, unplanned revascularisation, hospitalisation for cardiac cause   (c) Requirement for non-invasive cardiac investigations  (d) Requirement for invasive coronary angiography  (e) Procedural complications |
|  | To compare the effect on general wellbeing between the two groups at 9 months. | QoL questionnaire  Patient satisfaction questionnaire  Angina status  Time to definitive management plan  Time to completion of initial management  Number of hospital attendances  Working days lost |

# STUDY DESIGN

FORECAST is a randomised controlled trial comparing 1400 patients with new onset chest pain who are prospectively assigned to either routine assessment or FFR_CT_ assessment. Test interpretation and care decisions are to be made by the clinical care team.

## Definition of End of Study

End of study is defined as when the last patient has had their last data collected, cleaned and verified.

# SITE INITIATION

Each investigational site will complete a site initiation process to ensure the site’s data meets CT quality standards prior to enrolling subjects in the study. The initiation process will include peer-to-peer training and discussions on quantitative CT coronary angiography. Investigational sites new to HeartFlow studies shall provide 10 consecutive blinded and anonymized cCTA cases to HeartFlow to be assessed for image quality. Sites with a > 20% rejection rate will have one additional opportunity to provide 10 additional cases to HeartFlow. If the site is still unable to meet the quality guidelines, participation in the study may be terminated.

# SELECTION AND ENROLMENT OF PARTICIPANTS

## eLIGIBILITY SCREENING

**Prospective recruitment route:**

- All patients referred to the hospital RACPC may be screened for study eligibility. Information available from their medical records, recent diagnostic tests or from the patient's primary care physician may be obtained to find potentially eligible subjects. This can be done by the patient’s own clinical care team and referral made to the research team or directly by the research team with the consent of the clinical care team. The research team may directly screen for eligibility, and potential participants will then be invited to discuss the study with the research team.

We are sensitive to the need for potential participants to be given adequate time to consider the study yet there may also be a need for a decision to be made within a short period of time.

**Letter of invitation:**

- Where the opportunity arises, the clinical or research team can send a letter of invitation, at the same time as confirming the patients RACPC appointment, with the PIS included.

**Leaflets and posters displayed in clinic**

- Posters and leaflets will be provided to clinics to display within the RAPC

## Consent

Signed participant consent must be received. The right of the participant to refuse to participate without giving reasons must be respected. After the participant has entered the study the clinician remains free to give alternative treatment to that specified in the protocol at any stage if he/she feels it is in the participant’s best interest, but the reasons for doing so should be recorded. In these cases the participants remain within the study for the purposes of follow-up and data analysis. All participants are free to withdraw at any time from the protocol without giving reasons and without prejudicing further treatment.

Upon completion of the informed consent form, the original will be stored in the local Investigator Site File, a copy will be given to the patient, a copy will be stored in the patient’s medical notes and a copy will be sent to the SCTU. The SCTU copy should be emailed to uhs.[sctu@nhs.net](mailto:sctu@nhs.net) using a secure nhs.net email address to allow for central monitoring.

## Inclusion Criteria

- Aged over 18
- Primary symptom of chest pain who are deemed to require a test at initial RACPC assessment
- Willing and able to provide written informed consent

## Exclusion Criteria

- Patient does not require cardiac tests after initial RACPC assessment
- Unstable angina or evidence of acute coronary syndrome
- Atrial fibrillation of new onset or when rate control has been difficult
- Known bigeminy/trigeminy
- Prior coronary revascularisation (either stent or surgery)
- Contraindication to CTA, including:
  - Presence of permanent pacemaker or internal defibrillator
  - Known allergy to iodinated contrast
  - Pregnancy
  - Contraindication to intravenous beta blockade
  - Contraindication to acute sublingual nitrate administration
  - Prosthetic heart valve
- Advanced renal impairment (creatinine >200umol/L)
- Significant valve disease (severe aortic stenosis or regurgitation; severe mitral regurgitation)
- Life expectancy <12 months
- Complex congenital heart disease
- Inclusion in other study with ongoing follow up
- Intention to request FFR_CT_ for clinical reasons outside of this randomised group

## Screening failures

The research team at each site will complete a screening log detailing each time a potential participant is approached and document their decision to participate in the FORECAST Trial. Patients who are screen failures will have their initials, month and year of birth, and reason for failure recorded on the screening log. Screening logs will be sent to the trial manager every 2 months.

## Randomisation Procedures

Once eligibility for the trial is confirmed and informed consent received, patients will be enrolled in the study and randomised to a management group via an independent, web-based system (TENALEA). This online system allows for instant assignment to either the routine assessment or FFR_CT_ assessment, 24 hours per day.

This service will be provided by the Southampton Clinical Trials Unit, with telephone back up during office hours.

# STUDY OBSERVATIONS AND PROCEDURES

## data entry

A bespoke, web-based electronic case report form (CRF) has been developed to capture the study specific data. Data will be entered by local trial personnel. Computer records will be held on a secure server, managed and provided by Medidata and will be protected by password access. Checks for data consistency and validity will be performed at the point of data entry.

## Study Procedures

**Baseline**

- Inclusion/exclusion criteria including diagnostic testing
- Informed consent
- General medical history; demographics; height and weight and cardiac risk factors
- Concomitant medication
- Questionnaires (see below)
- Randomisation

After all inclusion and exclusion criteria are met and the informed consent document is signed, subjects will complete the baseline Seattle Angina Questionnaire – Short Form (SAQ-7), EQ-5D-5L quality of life questionnaire and Treatment satisfaction questionnaire, whilst or prior to proceeding to randomisation and diagnostic testing.

At the point of randomisation, the research team will establish to which routine investigation the participant would be allocated.

Patients will then be randomised to one of the two groups.

**Routine Assessment (Reference Group)**

All patients in the ‘routine assessment’ group will be assessed according to the clinical algorithm used by the local Trust, which will be based on NICE guidelines for Chest Pain of Recent Onset (either the 2010 or 2016 version of CG 95, depending upon which version the local practice is following at the time of patient recruitment).

Anticipated possible management outcomes include: ETT, stress echo, stress MR, nuclear perfusion, CTA, CT calcium score, invasive CA. All of these are in accordance with the local application of the NICE guideline for chest pain of recent onset.

NB: It is anticipated that, on occasion, the CTA will lead to a report of incidental findings in the lung, such as masses or abnormal nodes. Such findings should be dealt with in accordance to local clinical practice pathways. Any additional investigations arising from such incidental findings will be included in the resource utilisation endpoint.

**FFR_CT_ (Test Group)**

In the FFR_CT_ group, all patients who are eligible for CTA will undergo CTA as their default test. In all cases the CTA will be performed within the time frame that represents the standard waiting time for this test in that Trust. The results of the CTA will determine whether the FFR_CT_ will be performed.

Those patients with a coronary stenosis of >40% in at least one major epicardial vessel of stentable/graftable diameter will be referred for FFR_CT_. (*NB Lesions in distal vessels beyond the reach of stents or grafts or vessels of a diameter not suitable for stenting/grafting will not qualify for FFR_CT_ if there are no other more significant lesions*).

FFR_CT_ will be performed by HeartFlow, independently of clinical assessment. A secure web transfer portal will be established with each site allowing transfer of the raw CTA data to HeartFlow where analysis according to their published FFR_CT_ protocol will be undertaken. The FFR_CT_ output will be returned to the investigating site within 1 working day.

In patients in whom FFR_CT_ analysis is performed, FFR will be derived for all vessels. The data derived from this test will determine their management strategy. The patients in this arm will *not follow* the CG95 NICE guideline algorithm.

- Patients receiving a negative FFRct from HeartFlow

Investigators are strongly discouraged from referring these patients for an invasive coronary angiogram - only in exceptional circumstances. Justification will be requested via the eCRF.

- Patients receiving a positive FFRct fromHeartFlow

Investigators are strongly discouraged from performing an invasive pressure wire assessment at the time of invasive coronary angiography – only in exceptional circumstances. Justification will be requested via the eCRF

Those patients randomised to FFR_CT_ who are **not** referred to FFR_CT_ due to having no coronary stenosis of >40% in at least one major epicardial vessel of stentable/graftable diameter will be treated as per discretion of their supervising physician.

NB: It is anticipated that, on occasion, the CTA will lead to a report of incidental findings in the lung, such as masses or abnormal nodes. Such findings should be dealt with in accordance to local clinical practice pathways. Any additional investigations arising from such incidental findings will be included in the resource utilisation endpoint.

## Follow up

All participants will be included in the study from randomisation (time point 0) until 9 months post randomisation. In addition, all patients will be asked to consent for longer term follow up of tracked clinical events & mortality from 2-10 years.

Follow up will take place at 3 months and 9 months following randomisation, within a timeframe of ±15 days from the due date.

Data will be collected according to detailed specialised methodology for tracking:

(i) **Resource Utilisation** - including requirement for non-invasive cardiac investigations,

Invasive angiography, Revascularisation (including PCI & CABG), Hospitalisation for cardiac event (including MI/unstable angina/revascularisation/heart failure/arrhythmia), Cardiac medications. Any additional investigations arising from the observation of incidental abnormal findings on CTA in either group will also be included.

(ii) **QOL** assessed using the EQ-5D-5L quality of life questionnaire,

(iii) **Angina status** using the Seattle Angina Questionnaire – Short Form (SAQ-7)

(iv) **Treatment satisfaction** questionnaire.

(v) **Clinical Events** including: all cause death, non-fatal MI, all coronary revascularisation (PCI + CABG), any cardiac-related hospital attendance/admission.

Questionnaires will be used to collect quality of life and patient satisfaction endpoints (EQ-5D-5L SAQ-7, and treatment satisfaction questionnaires). In order to maximise the collection of the data obtained, participants will consent to be contacted by telephone for follow-up information at 3 and 9 months. Sites will have an option of posting or emailing questionnaires where this is preferable for the patients/sites.

Patients who have given their consent will be subject to longer-term follow-ups, and may have certain clinical events collected via access to data warehouses such as HES data and ONS mortality. Participants can be included in the main study if they decline the long term follow-up.

## AssessmEnt window

Follow-up contact for questionnaire data should be completed within the following time windows from the date of randomisation

- ± 15 days from the due date

## Deviations and serious Breaches

Any study protocol deviations and breaches of Good Clinical Practice occurring at sites should be reported to the SCTU and the local R&D Office immediately. SCTU will then advise of and/or undertake any corrective and preventative actions as required.

All serious protocol deviations and serious breaches of Good Clinical Practice and/or the study protocol will immediately be reported to the regulatory authorities and other organisations, as required in the Medicines for Human Use (Clinical Trials) Regulations 2004, as amended.

## Withdrawal

The participant is free to withdraw consent from the study at any time without providing a reason.

If a participant initially consents but subsequently withdraws from the trial, a clear distinction must be made as to what aspect of the trial the participant is withdrawing from.

These aspects could be:

1. Withdrawal from trial intervention.
2. Withdrawal from further study follow-up.
3. Withdrawal from entire study and does not want data to be used.

Investigators should explain to participants the value of remaining in study follow-up and allowing this data to be used for trial purposes. If patients additionally withdraw consent for this, they should continue with clinical care as deemed appropriate by the responsible clinician. It would remain useful for the study team to continue to collect standard follow-up data and unless the patient explicitly states otherwise, follow-up data will continue to be collected.

Details of study discontinuation (date, reason if known) should be recorded in the eCRF and medical record.

# SAFETY

## Definitions

**Adverse Event (AE):** any untoward medical occurrence in a participant or clinical study participant which does not necessarily have a causal relationship with study treatment or participation.

An AE can therefore be any unfavourable and unintended sign (including an abnormal laboratory finding), symptom, or disease temporally associated with the study treatment or participation (regardless of causality assessments).

**Serious Adverse Event** **(SAE)** is any untoward medical occurrence or effect that is associated with the test investigation (FFRct) and:

- **Results in death**
- **Is life-threatening** – *refers to an event in which the participant was at risk of death at the time of the event; it does not refer to an event which hypothetically might have caused death if it were more severe*
- **Requires hospitalisation, or prolongation of existing hospitalisation***
- **Results in persistent or significant disability or incapacity**
- **Is a congenital anomaly or birth defect**
- **Other important medical events****

*Hospitalisation is defined as an inpatient admission, regardless of length of stay, even if the hospitalisation is a precautionary measure for continued observation. Hospitalisations for a pre-existing condition, including elective procedures that have not worsened, do not constitute an SAE.

**Other important medical events may also be considered serious if they jeopardise the participant or require an intervention to prevent one of the above consequences.

**Note:** It is the responsibility of the PI or delegate to grade an event as ‘not serious’ (AE) or ‘serious’ (SAE).

## Seriousness

A complete assessment of the seriousness must always be assessed by a medically qualified doctor who is registered on the delegation of responsibility log; this is usually the investigator.

All adverse events that fulfil the criteria definition of ‘serious’ in protocol section 7.1 and are considered related to the Test group – CTCA ± FFRct, must be reported to SCTU using the Trial Related Serious Adverse Event Report Form – Non-CTIMP.

All Trial related SAEs must be reported immediately by the PI at the participating site to the SCTU.

7.2.1 SAFETY ENDPOINTS

The trial will collect specific data on the following study endpoints. These should be reported on the associated follow up eCRF.

- All cause mortality
- Non-fatal myocardial infarction
- All coronary revascularisation (PCI & CABG)
- Invasive coronary angiography
- Complications arising from invasive coronary procedures
- Hospitalisation for cardiac event
- Cerebrovascular accident/transient ischaemia attack
- Procedure related bleeding requiring transfusion or prolonging hospital stay

## Causality

A complete assessment of the causality must always be assessed by a medically qualified doctor who is registered on the delegation of responsibility log; this is usually the investigator.

If any doubt about the causality exists the local investigator should inform the SCTU who will notify the Chief Investigator. Other clinicians may be asked for advice in these cases.

| Relationship | Description | Event Status | |
| --- | --- | --- | --- |
| **Unrelated** | There is no evidence of any causal relationship | | Not related to treatment |
| **Unlikely** | There is little evidence to suggest there is a causal relationship (e.g. the event did not occur within a reasonable time after administration of the study treatment). There is another reasonable explanation for the event (e.g. the participant’s clinical condition, other concomitant treatment). | | Not related to treatment |
| **Possibly** | There is some evidence to suggest a causal relationship (e.g. because the event occurs within a reasonable time after administration of the study treatment). However, the influence of other factors may have contributed to the event (e.g. the participant’s clinical condition, other concomitant treatments). | | Related and expected SAE/  Related and unexpected SAE |
| **Probably** | There is evidence to suggest a causal relationship and the influence of other factors is unlikely. | | Related and expected SAE/ Related and unexpected SAE |
| **Definitely** | There is clear evidence to suggest a causal relationship and other possible contributing factors can be ruled out. | | Related and expected SAE/ Related and unexpected SAE |

In the case of discrepant views on causality between the Investigator and others, SCTU will classify the event as per the worst case classification and, where applicable, the Ethics Committee will be informed of both opinions within the required timelines.

## Expectedness

Expectedness assessments are made against the list of expected events below:

Expected Adverse Events**:**

| **CTA-related**  Anaphylaxis  Other Contrast allergy  Acute renal failure  Bradycardia requiring treatment |
| --- |

The nature or severity of the event should be considered when making the assessment of expectedness. If these factors are not consistent with the current information available then the AE should be recorded as ’unexpected’.

## Reporting Procedures

No Adverse events will be collected.

The reporting requirement for SAEs affecting participants applies to the investigation arm undergoing the FFR_CT_ (CT Coronary Angiography with FFR)

All trial related SAE’s and protocol listed endpoints should be reported. Depending on the nature of the event, the appropriate reporting procedures below should be followed. A flowchart will be provided to aid in the reporting procedures.

### REPORTING DETAILS

A SAE for Non-CTIMPs Form should be completed for all trial related SAEs and faxed to SCTU within 24 hours of site becoming aware of the event.

Complete the trial related SAE form and fax or email a scanned copy of the form with as many details as possible to the SCTU together with anonymised relevant treatment forms and investigation reports.

**SAE REPORTING CONTACT DETAILS**

***Please email or fax a copy of the SAE form to***

***SCTU within 24 hours of becoming aware of the event***

**Fax: 0844 774 0621 or Email: ctu@soton.ac.uk**

FAO: Quality and Regulatory Team

***For further assistance: Tel: 023 8120 4138 (Mon to Fri 09:00 – 17:00)***

Additional information should be provided as soon as possible if the event has not resolved at the time of reporting.

### 7.5.2 FOLLOW UP SAEs

All unresolved serious adverse events should be followed by the investigator until resolved, the participant is lost to follow-up, or the adverse event is otherwise explained.

**7.5.3 Trial related SERIOUS ADVERSE EVENTS**

All SAEs should be reported within 24 hours of the local site becoming aware of the event. The trial related SAE Non-CTIMP Form asks for nature of event, date of onset, severity, corrective therapies given, outcome, causality (i.e. unrelated, unlikely, possible, probably, definitely) and expectedness. The responsible investigator should assign the causality and expectedness of the event with reference to the events listed in Section 7.4. The event term should be in accordance with the latest version of MedDRA Additional information should be provided as soon as possible if the event has not resolved at the time of reporting.

## SCTU Responsibilities For Safety Reporting to rec

SCTU will notify the necessary competent authorities of all **Related and Unexpected** SAEs occurring during the study within 15 days.

SCTU submit all safety information to the REC in annual progress report.

# STATISTICS AND DATA ANALYSES

## Method of randomisation

Patients will be randomised to either Routine Assessment group (Reference Group) or FFR_CT_ group (Test Group) on a 1:1 ratio using varying block size randomisation.

## Sample size

The recently published PLATFORM economic analysis compared detailed (non-randomised) costs of an FFR_CT_ strategy compared with usual care, stratified by the initial intention to perform either conventional non-invasive evaluation or a conventional invasive evaluation.^25^ This economic analysis, taking into consideration the UK healthcare costing environment, provided the basis for the following sample size calculation.

In order to ensure plausible output for the UK health system, data from the University Hospital Southampton NHS Foundation Trust RACP for the calendar year 2015 were introduced into the economic model (see Appendix 2).

The PLATFORM study observed relatively large differences in per-patient costs within the invasive stratum (32% change), and smaller differences in the per-patient cost within the non-invasive stratum (25% change). Usual care in FORECAST will differ somewhat from that used in PLATFORM, as non-invasive evaluation in the Rapid Chest Pain Units rely upon stress imaging, which is more costly than the exercise ECG studies used frequently in PLATFORM. Cost differences of 20% based on use of FFR_CT_ in FORECAST would be both plausible and important to detect.

Since cost distributions are skewed, the analysis of cost data in FORECAST will use non-parametric methods. A log transformation of costs will reduce the skew, and the following calculations are based on log transformation of the cost data. The standard deviations of the ln(cost) data from PLATFORM provide estimates of the variability we might observe. In the non-invasive cohort of PLATFORM, the SD (ln(cost)) was in the range of 1.2 to 1.5. The sample sizes necessary to detect a 20% difference between groups in cost (a mean difference of 0.223 between the log of the costs), at different levels of power, based upon plausible ranges of the variability of costs (i.e., standard deviation of ln(cost) between 1.2 and 1.5) are summarized in the table:

| **20% cost difference between groups, in Ln(cost) units** | **Standard deviation of Ln(cost)** | **N patients per group for 90% Power** | **N patients per group for 85% Power** | **N patients per group for 80% Power** |
| --- | --- | --- | --- | --- |
| 0.223 | 1.2 | 609 | 520 | 455 |
| 0.223 | 1.3 | 712 | 609 | 532 |
| 0.223 | 1.4 | 826 | 706 | 617 |
| 0.223 | 1.5 | 948 | 810 | 708 |

A sample size of 700 patients per group would provide approximately 90% power to detect a 20% difference in costs between groups, assuming no loss to follow-up and moderate cost variability (a standard deviation of Ln(cost) of 1.3). A loss to follow-up of as much as 12% (616 patients followed up) would still provide 85% power to detect a 20% cost difference in the setting of moderate cost variability (SD 1.3), and provide 80% power if there is higher cost variability (SD 1.4). Thus, a sample size of 700 patients per group, 1,400 in total, should provide sufficient power to detect meaningful, 20% differences in cost under a range of plausible degrees of variations in cost, even with larger (12%) losses to follow-up than are anticipated in the trial.

## Interim Analysis

There will be no interim analysis

## Statistical Analysis Plan (SAP)

The analysis of the primary outcome will use an intention-to-treat approach, and compare 9-month costs per patient between individuals who were randomly assigned to FFR_CT_ and individuals who were randomly assigned to conventional care. Each patient’s 9-month total cost of medical care for cardiac disease will be tabulated, and compared using non-parametric tests (e.g., the Wilcoxon rank-sum test) or a 2 sample t-test based on means of ln(cost) in the two groups, because of the expected skew in 9-month cost data.

We will also perform a stratified analysis based on whether patients were designated prior to randomisation to receive non-invasive testing or invasive testing if assigned to the conventional strategy. We will test for differences in 9-month costs in each stratum, and test for statistical interaction between stratum and treatment assignment, to determine whether the effect on 9-month cost of assignment to FFR_CT_ varies depending on whether conventional testing approach would have been invasive or non-invasive.

We do not expect important imbalances in baseline characteristics or length of follow-up between randomly assigned groups, but if any are present, we can use a linear regression model, with ln(cost) as the dependent variable, to adjust for any such differences. This regression model will also be used to examine the effects of factors other than random treatment assignment on 9-month cost, including factors such as age, sex, and the intended initial diagnostic test under the conventional strategy that was declared prior to randomisation.

The statistical analysis of the trial will be conducted according to the International Conference on Harmonisation E9 guidelines and reported using the ‘Consolidation Standard of Reporting Trials’ (CONSORT) guidelines. A full and detailed statistical analysis plan will be developed prior to the final analysis of the trial. The main features of the statistical analysis plan are included below:

Baseline characteristics will be presented but no comparisons will be undertaken, rather the clinical importance of any imbalance will be noted. Kaplan-Meier survival curves and Cox-regression modelling will be used for time to event outcomes. Continuous data will be presented as means and standard deviations and analysed using ANCOVA (if data is skewed, medians and ranges will be presented and analysis will be by Mann Whitney U tests). Binary data will be reported in terms of odds ratios and analysed using logistic regression modelling. A two-sided p-value of 0.05 or less will be used to declare statistical significance for all analyses and results will be presented with 95% confidence intervals.

The analysis of the binary clinical outcomes will be based on frequency of the events and conducted using logistic regression, within an intention-to-treat framework. The patient reported outcomes at 9 months, such as Seattle Angina Questionnaire and EQ-5D, will be compared between randomly assigned patients using non-parametric tests (e.g., the Wilcoxon rank-sum test)

# REGULATORY

## Clinical Trial Authorisation

This study is not considered to be a clinical trial of a medicinal product, so clinical trial authorisation from the UK Competent Authority the Medicines and Healthcare products Regulatory Agency (MHRA) is not applicable.

# ETHICAL CONSIDERATIONS

The study will be conducted in accordance with the recommendations for physicians involved in research on human participants adopted by the 18th World Medical Assembly, Helsinki 1964 as revised and recognised by governing laws and EU Directives. Each participant’s consent to participate in the study should be obtained after a full explanation has been given of the investigation options, including the conventional and generally accepted methods of investigations. The right of the participant to refuse to participate in the study without giving reasons must be respected.

After the participant has entered the study, the clinician may give alternative investigations to that specified in the protocol, at any stage, if they feel it to be in the best interest of the participant. However, reasons for doing so should be recorded and the participant will remain within the study for the purpose of follow-up and data analysis according to the treatment option to which they have been allocated. Similarly, the participant remains free to withdraw at any time from protocol treatment and study follow-up without giving reasons and without prejudicing their further care.

## specific ethical considerations

The SCTU uses the electronic data capture tool called RAVE, which will be used in the FORECAST trial for sites to input anonymised trial data. The servers that this database will be held on are based in the USA and therefore being stored outside of the UK and EEA. The Patient Information Sheet and Informed Consent Form shall highlight to patients where the data shall be held.

CT scan data shall be exported out of the UK to the USA. All data that is transferred to the USA will hold no personal data and will remain anonymous. All appropriate contracts and sending requirements shall be met and patients shall be informed through the use of the Patient Information Sheet and Informed Consent Form.

## ETHICAL APPROVAL

The study protocol has received the favourable opinion of a UK based Research Ethics Committee

## INFORMED CONSENT PROCESS

Informed consent is a process that is initiated prior to an individual agreeing to participate in a study and continues throughout the individual’s participation. In obtaining and documenting informed consent, the investigator should comply with applicable regulatory requirements and should adhere to the principles of GCP.

Discussion of objectives, risks and inconveniences of the study and the conditions under which it is to be conducted are to be provided to the participant by appropriately delegated staff with knowledge in obtaining informed consent with reference to the patient information leaflet. This information will emphasise that participation in the trial is voluntary and that the participant may withdraw from the trial at any time and for any reason. The participant will be given the opportunity to ask any questions that may arise and provided with the opportunity to discuss the study with family members, friend or an independent healthcare professional outside of the research team and time to consider the information prior to agreeing to participate.

## CONFIDENTIALITY

SCTU will preserve the confidentiality of participants taking part in the study. The investigator must ensure that participant’s anonymity will be maintained and that their identities are protected from unauthorised parties. On CRFs participants will not be identified by their names, but by an identification code.

# SPONSOR

SCTU, Chief Investigator and other appropriate organisations have been delegated specific duties by the Sponsor and this is documented in the trial task allocation matrix.

The duties assigned to the study sites (NHS Trusts or others taking part in this study) are detailed in the Non-Commercial Agreement.

## INDEMNITY

For NHS sponsored research HSG (96) 48 reference no.2 applies. If there is negligent harm during the clinical study when the NHS body owes a duty of care to the person harmed, NHS Indemnity covers NHS staff, medical academic staff with honorary contracts, and those conducting the study. NHS Indemnity does not offer no-fault compensation and is unable to agree in advance to pay compensation for non-negligent harm. Ex-gratia payments may be considered in the case of a claim.

## FUNDING

HeartFlow Inc are funding this study.

### SITE PAYMENTS

The payments assigned to the study sites (NHS Trusts or others taking part in this study) are detailed in the Non-Commercial Agreement.

This study has been adopted onto the NIHR portfolio. This enables Trusts to apply to their comprehensive local research network for service support costs, if required.

**11.2.2 PARTICIPANT PAYMENTS**

Participants will not be paid for participation in this study.

## AUDITS AND INSPECTIONS

The study may be participant to inspection and audit by University Hospital Southampton NHS Foundation Trust (under their remit as Sponsor), SCTU (as the Sponsor’s delegate) and other regulatory bodies to ensure adherence to the principles of GCP, Research Governance Framework for Health and Social Care, applicable contracts/agreements and national regulations.

# STUDY OVERSIGHT GROUPS

The day-to-day management of the study will be co-ordinated through the SCTU and oversight will be maintained by the Trial Management Group, the Trial Steering Committee and the Data Monitoring and Ethics Committee.

## Trial Management Group (TMG)

The TMG is responsible for overseeing progress of the study, including both the clinical and practical aspects. The Chair of the TMG will be the Chief Investigator of the study.

The FORECAST TMG charter defines the membership, terms of reference, roles, responsibilities, authority, decision-making and relationships of the TMG*,* including the timing of meetings, frequency and format of meetings and relationships with other trial committees.

## trial steering committee (tsc)

The TSC act as the oversight body on behalf of the Sponsor and Funder. The TSC will meet at least yearly. The majority of members of the TSC, including the Chair, should be independent of the study.

The FORECAST TSC charter defines the membership, terms of reference, roles, responsibilities, authority, decision-making and relationships of the TSC*,* including the timing of meetings, frequency and format of meetings and relationships with other trial committees.

# DATA MANAGEMENT

Participant data will be entered remotely at site and retained in accordance with the current data protection regulations. The local PI is responsible for ensuring the accuracy, completeness, and timeliness of the data entered.

The participant data is pseudo anonymised by assigning each participant a participant identifier code which is used to identify the participant during the study and for any participant- specific clarification between SCTU and site. The site retains a participant identification code list which is only available to site staff.

The Informed Consent Form will specify the participant data to be collected and how it will be managed or might be shared; including handling of all Patient Identifiable Data (PID) and sensitive PID adhering to relevant data protection law.

Trained personnel with specific roles assigned will be granted access to the electronic case report forms (eCRF). eCRF completion guidelines will be provided to the investigator sites to aid data entry of participant information. Only the Investigator and personnel authorised by them should enter or change data in the eCRFs.

A Data Management Plan (DMP) providing full details of the study specific data management strategy for the trial will be available and a Trial Schedule with planned and actual milestones, CRF tracking and central monitoring for active trial management created.

Data queries will either be automatically generated within the eCRF, or manually raised by the study team, if required. All alterations made to the eCRF will be visible via an audit trail which provides the identity of the person who made the change, plus the date and time.

At the end of the study after all queries have been resolved and the database frozen, the PI will confirm the data integrity by electronically signing all the eCRFs. The eCRFs will be archived according to SCTU policy and a PDF copy including all clinical and meta data returned to the PI for each participant.

Data may be requested from the Data Access Committee at SCTU. Requests will be considered on a monthly basis.

# MONITORING

## Central Monitoring

Central monitoring of informed consent and patient flagging forms (if consented to) will be carried out at the SCTU. All forms will be kept in locked, secure cabinets and only authorised staff will have access.

Data stored at SCTU will be checked for missing or unusual values (range checks) and checked for consistency within participants over time. Any suspect data will be returned to the site in the form of data queries. Data query forms will be produced at SCTU from the trial database and sent electronically to a named individual (as listed on the site delegation log). Sites will respond to the queries providing an explanation/resolution to the discrepancies. There are a number of monitoring features in place at SCTU to ensure reliability and validity of the trial data, which are detailed in the trial monitoring plan.

## Clinical Site Monitoring

There will be a monitoring visit to each site in accordance with the trial monitoring plan.

### SOURCE DATA VERIFICATION

On receipt of a written request from SCTU, the PI will allow the SCTU direct access to relevant source documentation for verification of data entered onto the eCRF (taking into account current data protection regulations). Access should also be given to study staff and departments (e.g. Radiology).

The participants’ medical records and other relevant data may also be reviewed by appropriate qualified personnel independent from the SCTU appointed to audit the study. Details will remain confidential and participants’ names will not be recorded outside the study site.

## Source Data

Source documents are where data are first recorded, and from which participants’ CRF data are obtained. These include, but are not limited to, hospital records (from which medical history and previous and concurrent medication may be summarised), clinical and office charts, laboratory and pharmacy records, diaries, microfiches, radiographs, and correspondence.

# RECORD RETENTION AND ARCHIVING

Study documents will be retained in a secure location during and after the trial has finished.

The PI or delegate must maintain adequate and accurate records to enable the conduct of the study to be fully documented and the study data to be subsequently verified. After study closure the PI will maintain all source documents and study related documents. All source documents will be retained for a period of 10 years following the end of the study.

Sites are responsible for archiving the ISF and participants’ medical records.

The Sponsor is responsible for archiving the TMF and other relevant documentation.

# PUBLICATION POLICY

Data from all sites will be analysed together and published as soon as possible.

Individual investigators may not publish data concerning their patients that are directly relevant to questions posed by the trial until the Trial Management Group (TMG) has published its report. The TMG will form the basis of the Writing Committee and advise on the nature of publications. All publications shall include a list of investigators, and if there are named authors, these should include the Chief Investigator, Co-Investigators, Trial Manager, and Statistician(s) involved in the trial. Named authors will be agreed by the CI and Director of SCTU. If there are no named authors then a ‘writing committee’ will be identified.

SUMMARY OF SIGNIFICANT CHANGES TO THE PROTOCOL

e.g. [Populate as per CTU/FORM/5143]

| **Protocol date and version** | **Summary of significant changes** |
| --- | --- |
| Version 2  Dated 28^th^ September 2017 | Minor typographical errors corrected:  REC ref number updated.  IRAS project ID included  Total number of sites amended from 10 to 10-15  Schedule of observations and procedures – redundant row deleted  Inclusion criteria – informed consent duplicated – one deleted |
| Version 3  Dated  20^th^ March 2019 | Clarification to definition of MACCE;  Change to timescale for Follow ups to ±15 days from due date;  Clarification in section on informed consent to comply with SIV slides in relation to emailing consent forms to the CTU;  GDPR updates as per new regulation in May 2018  Updates to section 14.2 on Monitoring of sites |

# REFERENCES

1. Corbett S, Fox K, Curzen N. Optimal medical therapy in percutaneous coronary intervention patients: statins and ACE inhibitors as disease-modifying agents. Chapter 26 in Oxford Textbook of Interventional Cardiology. Oxford University Press, 2010. [↑](#endnote-ref-1)
2. Shaw LJ, Berman DS, Maron DJ et al. Optimal medical therapy with or without percutaneous coronary intervention to reduce ischemic burden: results from the Clinical Outcomes Utilizing Revascularization and Aggressive Drug Evaluation (COURAGE) trial nuclear substudy. *Circulation* 2008; 117: 1283-1291. [↑](#endnote-ref-2)
3. Hachamovitch R, Hayes SW, Friedman JD, Cohen I, Berman DS. Comparison of the short-term survival benefit associated with revascularization compared with medical therapy in patients with no prior coronary artery disease undergoing stress myocardial perfusion single photon emission computed tomography. *Circulation*. 2003; 107 : 2900-2907 [↑](#endnote-ref-3)
4. Farzaneh-ar A, Phillips H, Shaw L et al. Ischemia change in stable coronary artery disease is an independent predictor of death and myocardial infarction. *J Am Coll Cardiol Img* 2012; 5:715-24. [↑](#endnote-ref-4)
5. Longman K, Curzen N. Should ischaemia be the main target in selecting a percutaneous coronary intervention strategy? *Expert Rev Cardiovasc Ther* 2013;11:1051-9 [↑](#endnote-ref-5)
6. Curzen N. Is there evidence for prognostic benefit following PCI in stable patients? *Heart* 2010; 96:103-105. [↑](#endnote-ref-6)
7. Fihn S, Gardin J, Abrahms J et al. 2012 ACCF/AHA/ACP/AATS/PCNA/SCAI/STS Guidelines for the diagnosis and management of patients with stable ischemic heart disease. *J Am Coll Cardiol* 2012; 60:e44-164. [↑](#endnote-ref-7)
8. Patel M, Peterson S, Dai D, Brennan J, Redberg R, Anderson V, Brindis R, Douglas P. Low diagnostic yield of elective coronary angiography. *New England Journal of Medicine* 2010; 362:886-895. [↑](#endnote-ref-8)
9. Curzen N, Rana O, Nicholas Z, et al. Does routine pressure wire assessment influence management strategy at coronary angiography for diagnosis of chest pain? The RIPCORD Study. *Circulation: Cardiovascular Interventions* 2014; 7:248-55. [↑](#endnote-ref-9)
10. . Layland J, Oldroyd K, Curzen N, Sood A, Balachandran K, Das R, Junejo S, Ahmed N, Lee M, Shuakat A, et al. Fractional flow reserve vs. angiography in guiding management to optimise outcomes in non-ST segment elevation myocardial infraction: British Heart Foundation FAMOUS- NSTEMI randomised trial. *European Heart Journal* 2015; 36:100-11. [↑](#endnote-ref-10)
11. Toth G, Hamilos M, Pyxaras S et al. Evolving concepts of angiogram: fractional flow reserve discordances in 4000 coronary stenoses. *European Heart Journal* 2014; 35:2831-35. [↑](#endnote-ref-11)
12. Cooper A, Calvert N, Skinner J et al. Chest pain of recent onset: assessment and diagnosis of recent onset chest pain or discomfort of suspected cardiac origin. 2010 London: National Clinical Guideline Centre for Acute & Chronic Conditions. [↑](#endnote-ref-12)
13. Pijls NHJ, van Schaardenburgh P, Manoharan G et al. Percutaneous Coronary Intervention of functionally Non-significant Stenosis 5-Year Follow-Up of the DEFER Study*. J Am Coll Cardiol* 2007; 49: 2105-11 [↑](#endnote-ref-13)
14. Pijls NH, Fearon WF, Tonino PA et al. Fractional flow reserve versus angiography for guiding percutaneous coronary intervention in patients with multivessel coronary artery disease: 2-year follow-up of the FAME (Fractional Flow Reserve Versus Angiography for Multivessel Evaluation) study. *J Am Coll Cardiol*. 2010; 56(3): 177-84 [↑](#endnote-ref-14)
15. B De Bruyne, N Pijls, B Kalesan, et al. for the Fractional Flow Reserve versus Angiography for Multivessel Evaluation 2 (FAME 2) Trial investigators. Fractional Flow Reserve-Guided PCI versus Medical Therapy in Stable Coronary Disease. *New England Journal of Medicine* 2012; 367:991-1001. [↑](#endnote-ref-15)
16. De Bruyne B, Fearon W, Pijls N, Barbato E, Tonino P, Piroth Z, Jagic N, Mobius-Winkler S, Riouffol G, Witt N, Kala P, MacCarthy P, Engstrom T, Oldroyd K, Mavromatis K, Manoharon G, Verlee P, Frobert O, Curzen N, Johnson J, Limacher A, Nuesch E, Juni P. Fractional flow reserve-guided PCI for stable coronary artery disease. *New England Journal of Medicine* 2014; 371:1208-17. [↑](#endnote-ref-16)
17. Miller J, Rochitte C, Dewey M et al. Diagnostic performance of coronary angiography by 64-row CT. *New England Journal of Medicine* 2008;359;2324-36 [↑](#endnote-ref-17)
18. Neglia D, Rovai D, Chiara C et al. Detection of significant coronary artery disease by noninvasive anatomical and functional imaging. *Circulation: cardiovascular imaging* 2015;8:e002179 [↑](#endnote-ref-18)
19. The SCOT-HEART Investigators. CT coronary angiography in patients with suspected angina due to coronary heart disease (SCOT-HEART): an open label, parallel group, multicentre trial. *Lancet* 2015:385:2283-91. [↑](#endnote-ref-19)
20. Douglas P, Hoffman U, Patel M, et al. Outcomes of anatomical versus functional testing for coronary artery disease. *New Engl J Med* 2015:372:1291-1300. [↑](#endnote-ref-20)
21. Taylor C, Fonte T, Min J. Computational fluid dynamics applied to cardiac computed tomography for noninvasive quantification of fractional flow reserve: scientific basis. *J Am Coll Cardiol* 2013;61;2233-41. [↑](#endnote-ref-21)
22. Koo B, Erglis A, Doh J et al. Diagnosis of ischaemia-causing coronary stenosis by noninvasive fractional flow reserve computed from coronary computed tomographic angiograms. Results from the prospective multicentre DISCOVER-FLOW study. *J Am Coll Cardiol* 2011; 58:1989-97. [↑](#endnote-ref-22)
23. Norgaard N, Leipsic J, Gaur S et al. Diagnostic performance of non-invasive fractional flow reserve derived from coronary computer tomography angiography in suspected coronary artery disease. *J Am Coll Cardiol* 2014; 63:1145-55. [↑](#endnote-ref-23)
24. Douglas PS, Pontone G, Hlatky MA, Patel MR, Norgaard BL, Byrne RA, Curzen N, Purcell I, Gutberlet M, Rioufol G, Hink U, Schuchlenz HW, Feuchtner G, Gilard M, Andreini D, Jensen JM, Hadamitzky M, Chiswell K, Cyr D, Wilk A, Wang F, Rogers C, De Bruyne B; PLATFORM Investigators. [Clinical outcomes of fractional flow reserve by computed tomographic angiography-guided diagnostic strategies vs. usual care in patients with suspected coronary artery disease: the prospective longitudinal trial of FFRct: outcome and resource impacts study.](http://www.ncbi.nlm.nih.gov/pubmed/26330417) *European Heart Journal* 2015; 36:3359-67. [↑](#endnote-ref-24)
25. Hlatky M, de Bruyne B, Pontone G, Patel M, Norgaard B, Byrne R, Curzen N, Purcell I, Gutberlet B, Riouful G, Hink H, Schuchlenz H, feurchner G, Gilard M, Andreini G, Jensen J, Hadamitzsky J, Wilk M, Wang F, Rogers C, Douglas P. [Quality-of-Life and Economic Outcomes of Assessing Fractional Flow Reserve With Computed Tomography Angiography: PLATFORM.](http://www.ncbi.nlm.nih.gov/pubmed/26475205) *J Am Coll Cardiol*. 2015 Dec 1;66(21):2315-23

    # APPENDICES

    Appendix 1 - Algorithms for assessment of stable chest pain of recent onset in 2010 NICE guidelines

    Appendix 2 - Output from UH Southampton RACP for calendar year 2015

    APPENDIX 1

    Algorithms for assessment of stable chest pain of recent onset in 2010 NICE Guideline

    Appendix 2 Output from UH Southampton RACP for calendar year 2015

    Total patients seen: 1232

    Discharged without tests: 419

    Therefore. 1232-419 = 813 patients in whom tests were requested  (this is the specific cohort who will be randomised in FORECAST)

    **Of the 813 who had a test**…**the first test was**

    **Non –invasive tests total:  683 (84%)**

    541 (67%) had stress echo

    62  (8%) had MPS

    57  (7%) had stress CMR

    23 (3%) had CTCA

    **Direct invasive angio**

    69 (9%) invasive angio

    **After the non-invasive test as the first test.**

    124 (i.e. 18% of those referred for a non-invasive test, but 15% of the total who had any test- see yellow box in spreadsheet) were then referred for invasive angio [↑](#endnote-ref-25)
